# Ceftiofur treatment of sows results in long-term alterations in the nasal microbiota of the offspring that can be ameliorated by inoculation of nasal colonizers

**DOI:** 10.1186/s42523-023-00275-3

**Published:** 2023-10-20

**Authors:** Miguel Blanco-Fuertes, Marina Sibila, Giovanni Franzo, Pau Obregon-Gutierrez, Francesc Illas, Florencia Correa-Fiz, Virginia Aragón

**Affiliations:** 1 Centre de Recerca en Sanitat Animal (CReSA), Unitat Mixta d’Investigació IRTA-UAB en Sanitat Animal, Campus de la Universitat Autònoma de Barcelona (UAB), 08193 Bellaterra, Barcelona Spain; 2 IRTA, Programa de Sanitat Animal, Centre de Recerca en Sanitat Animal (CReSA), Campus de la Universitat Autònoma de Barcelona (UAB), 08193 Bellaterra, Barcelona Spain; 3 WOAH Collaborating Centre for the Research and Control of Emerging and Re-Emerging Swine Diseases in Europe (IRTA-CReSA), 08193 Bellaterra, Barcelona Spain; 4 Present Address: Ciber in Epidemiology and Public Health, Instituto de Salud Carlos III, Madrid, Spain; 5 Department of Animal Medicine, Production and Health (MAPS), University of Padua, 35020 Legnaro, PD Italy; 6 Selección Batallé, Avinguda dels Segadors, 17421 Riudarenes, Spain

## Abstract

**Background:**

The nasal microbiota of the piglet is a reservoir for opportunistic pathogens that can cause polyserositis, such as *Glaesserella parasuis*, *Mycoplasma hyorhinis* or *Streptococcus suis*. Antibiotic treatment is a strategy to control these diseases, but it has a detrimental effect on the microbiota. We followed the piglets of 60 sows from birth to 8 weeks of age, to study the effect of ceftiofur on the nasal microbiota and the colonization by pathogens when the treatment was administered to sows or their litters. We also aimed to revert the effect of the antibiotic on the nasal microbiota by the inoculation at birth of nasal colonizers selected from healthy piglets. Nasal swabs were collected at birth, and at 7, 15, 21 and 49 days of age, and were used for pathogen detection by PCR and bacterial culture, 16S rRNA amplicon sequencing and whole shotgun metagenomics. Weights, clinical signs and production parameters were also recorded during the study.

**Results:**

The composition of the nasal microbiota of piglets changed over time, with a clear increment of *Clostridiales* at the end of nursery. The administration of ceftiofur induced an unexpected temporary increase in alpha diversity at day 7 mainly due to colonization by environmental taxa. Ceftiofur had a longer impact on the nasal microbiota of piglets when administered to their sows before farrowing than directly to them. This effect was partially reverted by the inoculation of nasal colonizers to newborn piglets and was accompanied by a reduction in the number of animals showing clinical signs (mainly lameness). Both interventions altered the colonization pattern of different strains of the above pathogens. In addition, the prevalence of resistance genes increased over time in all the groups but was significantly higher at weaning when the antibiotic was administered to the sows. Also, ceftiofur treatment induced the selection of more beta-lactams resistance genes when it was administered directly to the piglets.

**Conclusions:**

This study shed light on the effect of the ceftiofur treatment on the piglet nasal microbiota over time and demonstrated for the first time the possibility of modifying the piglets’ nasal microbiota by inoculating natural colonizers of the upper respiratory tract.

**Supplementary Information:**

The online version contains supplementary material available at 10.1186/s42523-023-00275-3.

## Background

The relationship between the host and the bacterial communities in the swine microbiota from different tissues has been an issue exponentially assessed during the last decade [1–3]. As in other studies and animal species, bacterial communities are niche-specific [4]. The majority of the studies on the pig microbiome focus on the gut, but in recent years more studies are investigating other niches such as the skin, oropharyngeal and nasal cavities [2, 5, 6].

Recent microbiota studies focused on the specific factors that contribute to microbiota shifts, such as breed, feed system, environment or antimicrobial treatment among others [1, 7, 8]. Usually, these factors have a deeper impact when the microbiota is unstable or immature and, therefore, more susceptible to external changes [9–11]. Colonization of the respiratory tract starts at birth when most of the early colonizers are transferred from the dam and/or from environment [8]. Several studies have shown the importance of the microbiota population structure in the development of posterior gut and respiratory diseases [3, 4, 12]. For that reason, cross-sectional studies are key to expand the knowledge about the dynamics of the host-microbiome interactions.

In swine industry, the prevention of diseases during the postweaning period has a fundamental impact on the subsequent production phases [3, 13]. Weaning is a crucial moment in piglet’s life due to the implications in the maturation of the immune system and the intestine [14, 15]. In addition, immune protection acquired from the mothers starts to decline during this stage and, together with the stress and changes associated with weaning (separation from the sows, mixing of litters and social challenge, nutritional changes…), some pathologies caused by bacteria present in the normal microbiota (pathobionts) can be triggered [16, 17]. For instance, polyserositis is frequently observed in nursery pigs when members of the nasal microbiota, such as *Glaesserella parasuis*, *Streptococcus suis*, or *Mycoplasma hyorhinis*, spread systemically [16, 18].

Due to the lack of effective vaccines, the main strategy to control these diseases in piglets continues to be the use of antimicrobials [16, 19]. The use of antibiotics in sows is thought to reduce the transfer of pathogens to their offspring and control the pathogen load globally in the farm [20]. However, antimicrobials will also affect the composition of the beneficial microbiota [21]. Moreover, the indiscriminate use of antibiotics to control bacterial diseases (mostly as metaphylaxis) has been questioned due to the emergence of antimicrobial resistant bacteria. One of the alternative strategies to promote the health of piglets, and therefore reduce antibiotic usage in swine production, could be the use of microorganisms intended to provide benefits to the host (probiotics), and therefore pathogen exclusion. Interventions with probiotics may reduce or delay the colonization of the niche by pathogens. Probiotics are frequently used in humans but less commonly in pigs, where all of them target the gut microbiota [22, 23]. However, the use of probiotics in the respiratory tract has only been investigated in a few studies where they proved their variable action against respiratory-associated infections [24–27].

Here, we present the results obtained in a longitudinal study in a swine farm with respiratory problems, where ceftiofur was applied to pregnant sows or piglets. The findings of this study revealed a more prolonged effect on the piglets’ nasal microbiota when ceftiofur was administered to the sows than directly to the newborn piglets. We also demonstrated for the first time the ability to modify the nasal microbiota of the piglets by inoculating nasal colonizers at birth.

## Methods

### Study design

Animal experimentation was performed following proper veterinary practices, under European (Directive 2010/63/EU) and Spanish (Real Decreto 53/2013) regulation and with the approval of the Ethics Commission in Animal Experimentation of the Generalitat de Catalunya (Protocol number 9211).

A field study was conducted in a 2-site pig commercial farm with recurrent respiratory problems (site 1: gestation and lactation; site 2: nursery) located 15 km apart. The breeding herd (300 sows) was managed as a 1 week batch farrowing system and the duration of lactation was 3 weeks. After weaning, piglets were transferred to the nursery facility, where they stayed until nine weeks of age. Four days before farrowing (D-4), 23 sows were intramuscularly treated (treated sows) with 10 mL of crystalline ceftiofur, a third-generation cephalosporin (Naxcel®; 100 mg/mL), while 31 sows remained untreated (non-treated sows). With this product, maximum concentration of ceftiofur in plasma is reached within the following 22 h of intramuscular administration, and 75% of the drug is excreted within 10 days after administration. Ceftiofur is not expected to be found in milk. Sows were distributed in five different rooms in the lactation facilities (one group per room). Piglets were divided according to the treatment received by the sows, where 284 piglets born to treated sows and 407 born to non-treated sows were included in the study. To assess the effect of the antibiotic on the nasal microbiota of piglets when the treatment was applied either directly to them or to their sows, 3 groups were established (Table 1). At birth (D0), 129 piglets born to treated sows remained nontreated (TS group), while 115 piglets born to non-treated sows were intramuscularly treated with 1 mg of crystalline ceftiofur (Treated Piglet group = TP). As control, 118 piglets born to non-treated sows remained non-treated (control group).

**Table 1:** Number of sows and piglets per study group according to the sow and/or piglet treatment

Sow treatment (at 1 week pre-farrowing)	Piglet treatment (at birth)	Group*
Non-treated (NTS)	Non-Treated (n = 118 piglets from 10 sows)	Control
	Treated (n = 115 piglets from 9 sows)	TP
	Inoculated (n = 175 piglets from 12)	IP
Treated (TS)	Non-Treated (n = 129 piglets from 11 sows)	TS
	Inoculated (n = 154 piglets from 12 sows)	TS-IP

^*^For clarity, no acronym is explicitly mentioned when no treatment was applied

On the other hand, to study the effect of early colonization with selected colonizers on the piglets’ nasal microbiota, piglets were inoculated at D0 with a cocktail of 5 selected bacterial strains at 10^4^–10^5^ CFU/mL (dose of 200 µL) using a nasal spray (Table 1). These strains belonged to five different species: *Vagococcus lutrae*, *Streptococcus pluranimalium*, *Moraxella pluranimalium*, *Rothia nasimurium* and *Glaesserella parasuis*. The inoculated pigs were non-treated and originated from two different groups: 154 piglets were born to ceftiofur-treated sows (Treated sow − Inoculated piglet = TS − IP) and 175 were born to non-treated sows (IP group).

The number of born and weaned piglets per litter and group was registered. All piglets (n = 691) were followed during the first three weeks of age (lactation facilities) and after weaning a subset of them (n = 490) was followed until 8 weeks of age (nursery facilities), collecting data on the general conditions and health status of the animals (Table 2). During the nursery period, all the animals that showed any clinical signs were treated with antibiotics (1 mL / 10 kg of Gentamox®; 150 mg of amoxicillin and 40 mg of gentamicin per mL) and were removed from the study. Body weight was recorded at birth (D0) and at weaning (D21) and average daily weight gain (ADWG) was calculated through this period and compared among groups using a mixed effect linear model using lme4 R package [28]. The number of animals showing lameness or other clinical signs compatible with systemic infection and the associated mortality among groups were compared using Fisher test with Bonferroni correction [29]. The number of born and weaned piglets per group was analyzed by a Chi-squared test.

**Table 2:** Study design and actions performed to followed piglets born to treated or non-treated sows

Production phase	Day (D)	Action performed	Analysis	Non-treated sow			Treated-sow	
				Control (N = 10)	TP (N = 9)	IP (N = 12)	TS (N = 11)	TS-IP (N = 12)
Lactation	D0	Piglet treatment		0	115	0	0	0
		Piglet inoculation		0	0	175	0	154
		Weight		118	115	175	129	154
		Nasal sampling	*M. hyorhinis, G. parasuis* and *S. suis* PCRs^a^	0	10	0	10	0
			Microbiota 16S sequencing	0	5	0	5	0
	D7	Nasal sampling	*G. parasuis* and *S. suis* culture^b^	10	9	12	11	12
			*M. hyorhinis*, *G. parasuis* and *S. suis* PCRs^c^	50	45	60	55	60
			Microbiota 16S sequencing	5	5	5	5	5
	D15	Nasal sampling	*G. parasuis* and *S. suis* culture^b^	10	9	12	11	12
			*M. hyorhinis*, *G. parasuis* and *S. suis* PCRs^c^	50	45	60	55	60
Nursery	D21	Weight		101	85	118	89	97
		Nasal sampling	*G. parasuis* and *S. suis* culture^*b*^	10	9	12	11	12
			*M. hyorhinis*, *G. parasuis* and *S. suis* PCRs^c^	50	45	60	55	60
			Microbiota 16S sequencing	5	5	5	5	5
			WGS metagenomics	5	5	5	5	5
	D49	Nasal sampling	*G. parasuis and S. suis culture*^*b*^	10	9	12	11	12
			*M. hyorhinis, G. parasuis and S. suis* PCRs^*c*^	25	20	25	25	25
			Microbiota 16S sequencing	5	5	5	5	5
			WGS metagenomics	5	5	5	5	5

^a^5 animals were randomly selected among the 10 samples for PCR testing

^b^Corresponded to one piglet per litter

^c^Corresponded to five piglet per litter

**Control**, non-treated piglets born to non-treated sows; **TP**, piglets treated with ceftiofur born to non-treated sows; **IP**, inoculated piglets born to non-treated sows; **TS**, piglets born to treated sows; **TS-IP**, inoculated piglets born to treated sows

### Sample collection and processing

Nasal swabs were taken from piglets belonging to each group at different timepoints (D0, D7, D15, D21, D49) for bacterial culture, PCR or microbiota analysis, as described in Table 2.

Nasal swabs were resuspended in 500 µL of PBS and kept refrigerated until arrival at research facilities where they were vortexed and stored at − 20 °C. A total of 200 µL of the suspensions was processed using the Nucleospin Blood kit (Macherey–Nagel, Düren, Germany) according to the manufacturer’s instructions. Total extracted DNA was quantified using BioDrop DUO (BioDrop Ltd., Cambridge, UK) and stored at − 20 °C for further processing.

### PCR detection of *Glaesserella parasuis*, *Streptococcus suis*, and *Mycoplasma hyorhinis*

Swabs from 5 animals per litter from each group were used for pathogen detection by PCR. These five piglets were initially chosen randomly, and the same animals were tested by PCR at the different timepoints. If one of the piglets was not available at any timepoint, a littermate was chosen.

Detection of the pathogens present in the nasal cavity was done individually using specific PCRs on the 2 µl from the total extracted DNA. For *G. parasuis,* a specific *vtaA* leader sequence PCR [30] was used for the detection and differentiation of virulent or non-virulent strains. Specific PCRs for *S. suis* detection were performed, as well as the specific PCRs for serotype 2 and serotype 9 as described before [31, 32]. In the case of *M. hyorhinis*, qPCRs were performed as previously described [33]. Samples with a Ct value < 37 were considered positive.

PCR or qPCR results were expressed as percentage of positive samples per group and were compared through contingency tables with Chi-square test.

### Genotyping of *Glaesserella parasuis* and *Streptococcus suis* isolates

Swabs from 1 animal per litter from each group were used for bacterial culture. As above described for PCRs, the same animals were used at the different timepoints.

Swabs were plated on chocolate agar and up to 4 colonies morphologically compatible with *G. parasuis* and 4 colonies compatible with *S. suis* were selected for identification and characterization.

In order to discriminate different strains, *G. parasuis* and *S. suis* isolates were genotyped by enterobacterial repetitive intergenic PCR (ERIC-PCR) with primers ERIC-1F (ATGTAAGCTCCTGGGGATTCAC) and ERIC-2R (AAGTAAGTGACTG GGGTGAGCG) [34]. The PCR reaction mixture consisted of 3 mM of MgCl_2_, 1.2 μM of each primer, 0.23 mM of dNTPs, 0.75 U of GoTaq® polymerase (Promega, Madison Wisconsin, USA) and 100 nanograms of DNA sample. Amplification was carried out with an initial denaturation of 94 °C for 2 min followed by 30 cycles of 30 s at 94 °C, 1 min at 50 °C and 2.5 min at 72 °C, and finally an extension of 20 min at 72 °C.

### 16S amplicon sequencing and in silico analysis

Five animals per group were selected for longitudinal analysis (D7, D15, D21 and D49) of the nasal microbiota by 16S sequencing. At farrowing (D0), 5 piglets from non-treated and 5 piglets from treated sows were selected.

The region targeted for the Illumina 16S amplicon sequencing was the V3-V4. This region was amplified using primers 341F (5′- 516 CCTAYGGGRBGCASCAG-3′) and 806R (5′-GGACTACNNGGGTATCTAAT-3′) [35]. Read length was 2 × 250 bp and Illumina MiSeq technology was used [35]. The bioinformatic downstream analysis was done using Quantitative insights into microbial ecology (Qiime2) software toolkit [36]. Denoising and quality-control step was done using *q2-dada2* plugin [37]. The following step was to remove all the reads with no match with at least an 80% of identity against Greengenes (v13.8) rRNA database [38] and 50% of length. Diversity analyses were done using *core-metrics* plugin with a rarefied sample depth of 12,124. One sample was removed from the analysis due to not reach the query sample depth threshold. Alpha diversity was done using the Shannon diversity index [39], and Chao index [40]. Beta diversity distance matrices were calculated based on the weighted Unifrac index [41]. A permutation-based analysis of variance (PERMANOVA) [42] was done to test if the centroid of two or more groups were significantly different. The percentage of the variance explained by the study groups was calculated through the Adonis function from the Vegan package [43] in R. Taxonomic assignment of each amplicon sequence variant was done using the Qiime2 classifier (*q2-feature-classifier* plugin) trained with the V3-V4 region from 16S gene and the Greengenes database (v13.8) [38]. Analysis of composition of microbiomes with bias correction (ANCOM-BC) [44] function was done at each timepoint among all the groups to perform differentially abundant analysis at both amplicon sequence variant (ASV) level and collapsed at different taxonomic levels. All data was processed for tables, plots and figures using Rstudio [45] and qiime2R [46], ggplot2 [47] and tidyverse [48] packages.

### WGS metagenomic sequencing and in silico analysis

Extracted DNAs from the same swabs used for 16S sequencing at weaning (D21) and at the end of nursery (D49) were used for whole genome shotgun (WGS) metagenomics.

WGS metagenomic sequencing of the samples was done using Illumina Novaseq 6000 (2 × 150 bp) technology. The throughput required per sample was at least 15 Gigabases to acquire enough sequencing depth. Genomic data were analyzed under the biobakery3 [49] metagenomic profiling workflow. Raw reads were QC processed and trimmed using Kneaddata pipeline [49], which uses Trimmomatic [50] and Bowtie2 [51]. In addition, trimmed reads were aligned to the *Sus scrofa* reference genome v11.1 [52], to remove any read matching the host. Taxonomic profiling of each sample was assessed using Metaphlan4 [49, 53] software, on cleaned read level. Functional profiling of all the samples was done through HUMAnN [49] which quantifies gene families, EC enzyme modules, and pathways, using the UniRef [54] and MetaCyc databases [55]. Differences among groups were estimated through a multivariate association analysis with linear models using MaAsLin2 R package [56], embedded in the Biobakery3 toolkit. Abundances were passed through a basic filter requiring each pathway and taxa to have at least 0.01% abundance in at least 3% of all samples. Assembly of the clean reads was done using MetaSpades [57]. Taxonomic profiling of the metagenome-assembled genomes (MAGs) was done using Kraken2 [58].

Analysis of the antimicrobial resistance genes (AMR) was done using Abricate software [59] over the MAGs with the NCBI AMRFinderPlus database [60].

## Results

### Production parameters and health status

The mean number of liveborn piglets per litter was not significantly different among the groups (Table 3). No stillborns or mummies were recorded during farrowing.

**Table 3:** Number of liveborn and weaned piglets per litter, body weight and average daily weight gain per each treatment group

Groups	Farrow			Weaning			Farrowing to Weaning	
	Liveborn piglets		Body weight (Kg)	Weaned piglets		Body weight (Kg)	ADWG^#^ (gr)	
	Total	Mean per litter ± SD	Mean ± SD	Total	Mean per litter ± SD	Mean ± SD	n*	Mean ± SD
Control	118	13.10 ± 2.76	1.71 ± 0.35	108	10.80 ± 1.46	5.59 ± 1.08	101	0.18 ± 0.05
TS	129	13.08 ± 2.70	1.51 ± 0.35	122	11.08 ± 1.38	5.10 ± 0.95	89	0.17 ± 0.04
TP	115	12.58 ± 2.68	1.38 ± 0.34	97	10.77 ± 1.25	5.60 ± 1.34	85	0.19 ± 0.06
IP	175	13.39 ± 2.64	1.53 ± 0.36	149	12.41 ± 1.30	5.60 ± 1.35	118	0.17 ± 0.05
TS-IP	154	13.25 ± 2.71	1.58 ± 0.30	143	11.91 ± 1.67	5.51 ± 0.97	97	0.18 ± 0.04

^*^ The number of the animals weighed at weaning was lower than the number of weaned animals due to the fact that the rooms in the nursery facilities were smaller and not all the weaned animals included in the study could be allocated in them

^#^Only animals present at both timepoints were included

**Control, **non-treated piglets born to non-treated sows; **TS**, piglets born to treated sows; **TP,** piglets treated with ceftiofur born to non-treated sows; **IP**, inoculated piglets born to non-treated sows ; **TS-IP**, inoculated piglets born to treated sows

From the individual weights obtained at farrowing and at weaning (D21), the ADWG of the piglets was calculated and computed in a linear mixed effect model where the individual variation was computed as a random effect. No statistically significant differences between the mean body weight between groups at D0 (birth) and at D21 (weaning) were detected (Table 3).

During the lactation period (first three weeks of age), sporadic episodes of diarrhea were observed and, consequently, three animals from the control group were treated (gentamicin-amoxicillin). Throughout the nursery period, the main clinical signs observed were lameness and diarrhea, and piglets showing lameness were treated with gentamicin-amoxicillin (Table 4). To avoid any bias due to the effect of this antibiotic treatment on the microbiota, the gentamicin-amoxicillin treated animals were excluded from the study. During nursery, no significant differences were found in mortality rates among the groups. Notably, the TS-IP group showed a lower prevalence of lameness than the control group (*P* < 0.05, Chi-squared test; Table 4).

**Table 4:** Proportion (and percentages) of animals showing lameness and mortality rate compatible with respiratory or systemic disease at nurseries

Group	Lameness (Affected/total)	Mortality rate	
		Dead/total	Dead/affected
Control	48/101 (41%)	8/ 101 (7.90%)	8/ 48 (16.6%)
TS	36/100 (28%)	9/100 (9%)	9/36 (25%)
TP	NA	NA	NA
IP	47/118(28%)	11/118 (9.32%)	11/47 (23.4%)
TS-IP	24/96 (15%)*	1/96 (1.04%)	1/24 (4.16%)

*NA* Not available

^*^Significantly different when compared to control group (*P* < 0.05)

**Control**, non-treated piglets born to non-treated sows; **TS**, piglets born to treated sows; **TP**, piglets treated with ceftiofur born to non-treated sows; **IP**, inoculated piglets born to non-treated sows ; **TS-IP**, inoculated piglets born to treated sows

### Sow treatment induced long term changes in the nasal microbiota of the offspring

Globally, a total of 14,655,905 sequences were obtained by 16S amplicon (V3-V4) sequencing, with 12,084 different ASVs. Diversity analysis was calculated at a 12,124 read depth per sample, which required the elimination of one sample from the dataset due to low sequencing throughput (6,832 reads in a sample from the IP group at D7).

**Fig. 1:**
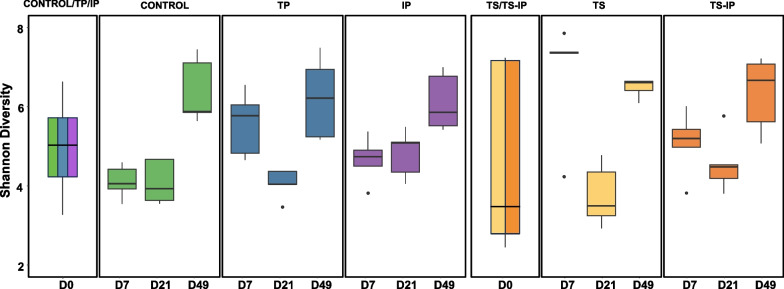
Alpha diversity (Shannon index) of nasal microbiota from piglet at different ages (D = days of age) and groups. Pregnant sows were treated with ceftiofur or remained untreated. A group of piglets born to treated sows remained nontreated (TS group), while piglets born to non-treated sows were treated with ceftiofur (TP group). A group of piglets were inoculated with selected colonizers of the upper respiratory tract either born to ceftiofur-treated sows (TS-IP) and to non-treated sows (IP group). As control, piglets born to non-treated sows remained non-treated

**Fig. 2:**
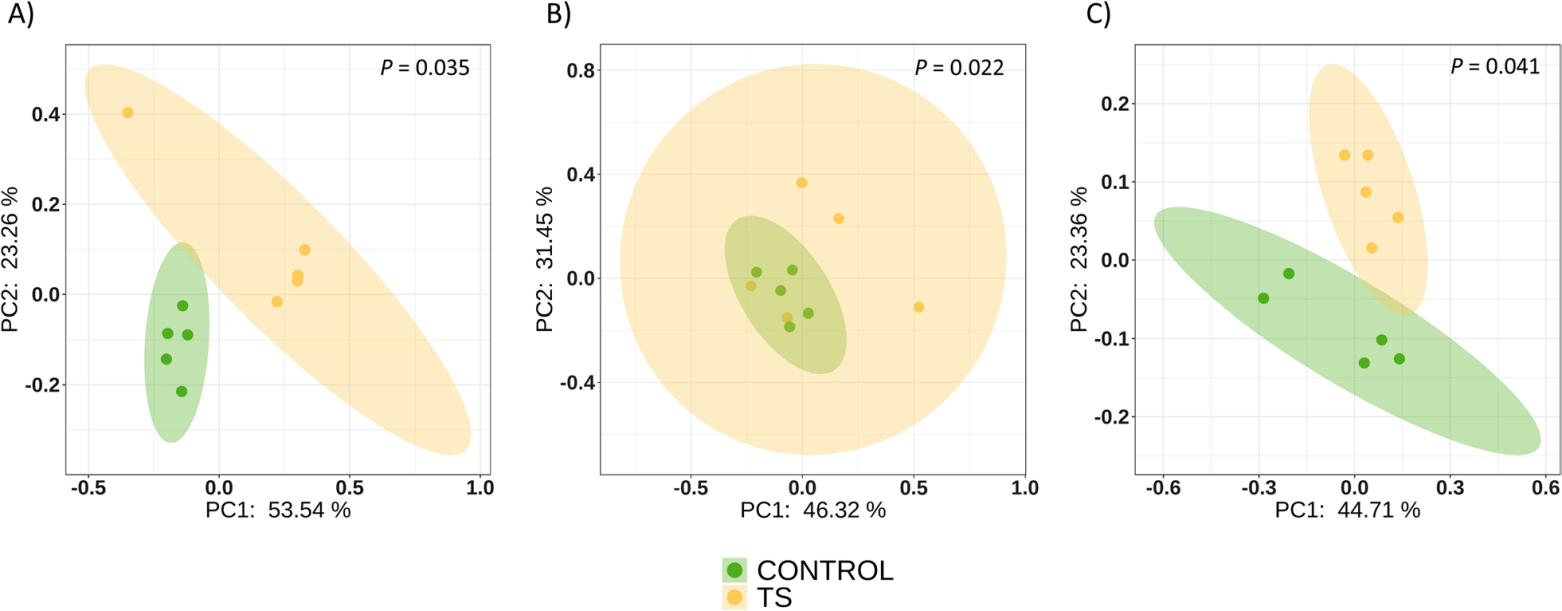
Beta diversity on weighted Unifrac distance matrices. PCoA was performed at D7 (**A**), D21 (**B**) and D49 (**C**). Orange dots represent samples from piglets born to ceftiofur treated sows (TS group) and green dots represent non-treated piglets born to non-treated sows (Control)

The effect induced by ceftiofur treatment in piglets’ nasal microbiota was evaluated when administered to sows before farrowing. Alpha diversity was estimated longitudinally (Chao and Shannon indexes), from birth to fattening at four time points. Initially (D0), alpha diversity was not different in piglets born to treated or non-treated sows, but piglets born to treated sows showed a higher inter-individual variability (Fig. 1). The treatment on sows induced a temporal increase in the alpha diversity of the nasal microbiota of piglets at D7 (Shannon diversity index *P* = 0.028) compared with the control group (Fig. 1). This rise in alpha diversity was not maintained through time and no significant differences were observed between TS and the control group at D21 or D49. When the beta diversity was analyzed, significant differences were detected at all sampling times, by either quantitative (Bray Curtis, weighted Unifrac) or qualitative (Jaccard, unweighted Unifrac) indexes. A stronger divergence was observed at D7 (Fig. 2), since the percentage of explanation measured on weighted Unifrac between groups was higher (R^2^ = 35%) at this timepoint compared to D21 (R^2^ = 28%) and D49 (R^2^ = 22%).

**Fig. 3:**
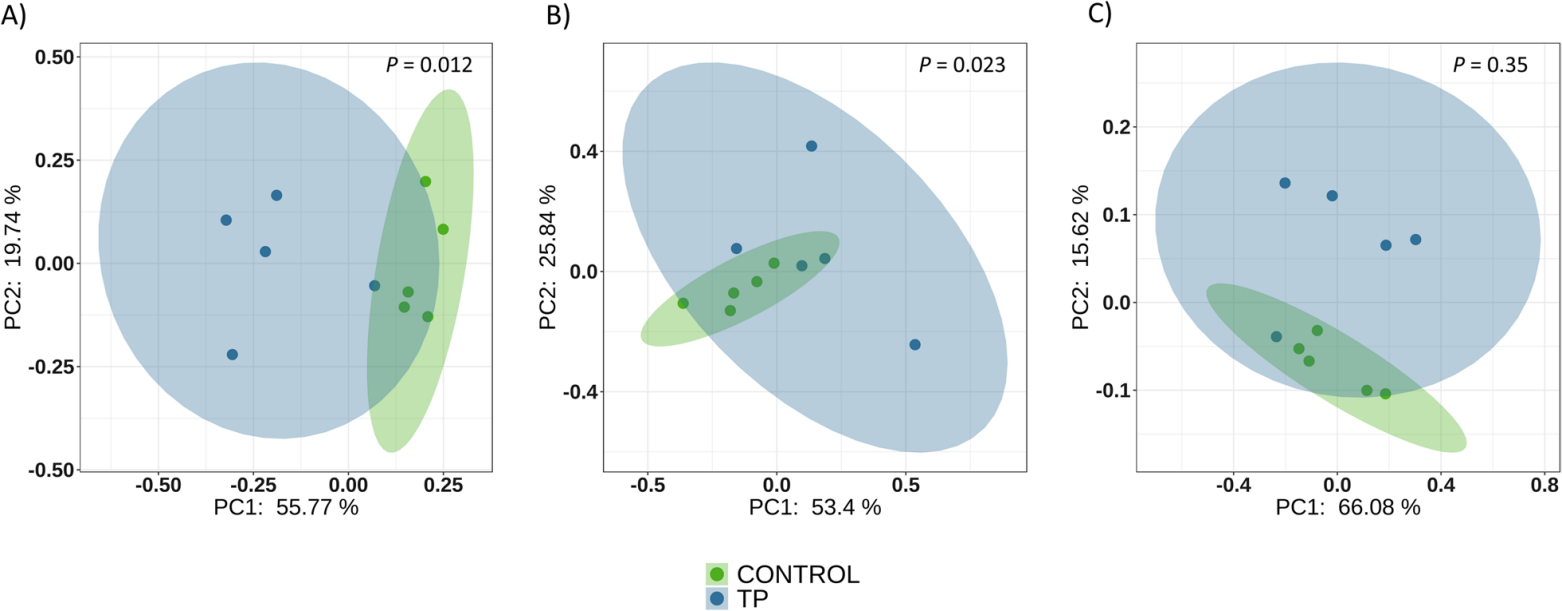
Beta diversity on weighted Unifrac distance matrices. PCoA was performed at D7 (**A**), D21 (**B**) and D49 (**C**). Blue dots represent samples from ceftiofur treated piglets born to non-treated sows (TP group) and green dots represent non-treated piglets born to non-treated sows (Control)

Ceftiofur treatment directly administered to piglets in early life (TP) produced similar trends in alpha diversity than the treatment applied to their sows (TS). A significant increase in alpha diversity (*P* = 0.009, Shannon diversity index) was detected at D7 in TP in comparison to the control group (Fig. 1). As observed in TP group, this increment was not maintained through time, and it was not observed at weaning (D21) or at the end of the study (D49). When beta diversity of TP and control groups was estimated, we detected differences at D7 and D21 using quantitative (Fig. 3) and qualitative metrics. At D49, the difference between these two groups was only detected using Jaccard distance (qualitative measurement). Importantly, the effect was more evident at D7 (Adonis function, R^2^ = 0.45, *P* = 0.012) than at the other timepoints (Adonis function, R^2^ = 0.24, and R^2^ = 0.15, at D21 and D49 respectively), underlying the different microbial composition in TP compared to the control soon after the antibiotic treatment.

Lastly, both treated groups (TP and TS) were compared to determine if the changes led by the antibiotic treatment were similar when administered to the piglets or to their sows. No significant differences were observed in alpha diversity at any sampling point. When beta diversity was analyzed, Jaccard, unweighted Unifrac and Bray Curtis metrics detected significative differences at D7 (*P* = 0.01, *P* = 0.026 and *P* = 0.03, respectively), but this difference was not observed using weighted Unifrac (Additional file 1: Fig. S1). Quantitative differences (weighted Unifrac and Bray Curtis) were not maintained at D21, while they were still detected with the qualitative analysis (Jaccard and unweighted Unifrac). Similar results that those found at D7 were obtained at D49 (unweighted Unifrac *P* = 0.006; Jaccard *P* = 0.008; Bray Curtis *P* = 0.012). The disparities observed in the beta diversity estimation, which detected significant differences when the analysis was made based on taxon presence-absence (qualitative metrics) but did not have the same outcome when taking into account also the relative abundance of these taxa (quantitative metrics), indicated that these qualitative differences were produced by bacterial taxa with low relative abundance.

To understand the microbial changes leading to these differences, we performed an analysis of the compositional microbiome with bias correction (ANCOM-BC) at each timepoint based on either amplicon sequence variants or at different taxonomic levels. At D7, we found 488 ASV in TS and 205 ASVs in TP that were differentially abundant when compared to the control group (ASVs; Additional file 2: Table S1), which was in agreement with the higher alpha diversity observed in this group. A significant increase in the relative abundance of ASVs from *Bacteroidales* (3.972% in TS, 3.534% in TP versus 0.357% in control) and *Clostridiales* (18.487% in TS, 7.733% in TP vs. 2.452% in control) was observed at D7 in both antibiotic-treated groups. On the other hand, typical members of the nasal microbiota were decreased in the treated groups with respect to the non-treated control group, including *Glaesserella* (at D21), *Moraxella* (at D7 and D21) and *Rothia* (at D21) (Table 5).

**Table 5:** Relative abundance of genera belonging to the core microbiota from control group at weaning from each group at D7 and D21

Taxa	D7			D21		
	Control	TS	TP	Control	TS	TP
*p__Proteobacteria;o__Pasteurellales;f__Pasteurellaceae;g__Glaesserella*	0.0183	0.0601	0.0017	0.2754	0.1094	0.1490
*p__Bacteroidetes;o__Flavobacteriales;f__[Weeksellaceae];g__Bergeyella*	0.0000	0.0250	0.0000	0.1063	0.2005	0.0679
*p__Proteobacteria;o__Pseudomonadales;f__Moraxellaceae;g__Enhydrobacter*	0.1252	0.0013	0.0010	0.0876	0.1265	0.1616
*p__Firmicutes;o__Lactobacillales;f__Streptococcaceae;g__Streptococcus*	0.0243	0.0249	0.0145	0.0320	0.0746	0.0368
*p__Proteobacteria;o__Caulobacterales;f__Caulobacteraceae;g__Caulobacter*	0.1005	0.0773	0.1593	0.0761	0.0801	0.1341
*p__Proteobacteria;o__Pseudomonadales;f__Moraxellaceae;g__Moraxella*	0.3518	0.0500	0.0476	0.0673	0.0298	0.0190
*p__Proteobacteria;o__Rhizobiales;f__Rhizobiaceae;g__Rhizobium*	0.0495	0.0307	0.0705	0.0402	0.0718	0.0785
*p__Firmicutes;o__Lactobacillales;f__Lactobacillaceae;g__Lactobacillus*	0.0174	0.0530	0.1236	0.0022	0.0095	0.0052
*p__Actinobacteria;o__Actinomycetales;f__Micrococcaceae;g__Rothia*	0.0069	0.0232	0.0100	0.0173	0.0026	0.0058
*p__Bacteroidetes;o__Bacteroidales;f__Bacteroidaceae;g__Bacteroides*	0.0008	0.0087	0.0214	0.0025	0.0018	0.0044
*p__Proteobacteria;o__Rhizobiales;__;__*	0.0495	0.0307	0.0705	0.0003	0.0009	0.0017
*p__Bacteroidetes;o__Bacteroidales;f__Prevotellaceae;g__Prevotella*	0.0008	0.0090	0.0064	0.0012	0.0015	0.0010
*p__Firmicutes;o__Clostridiales;f__Ruminococcaceae;g__*	0.0008	0.0046	0.0010	0.0020	0.0019	0.0009
*p__Proteobacteria;o__Pseudomonadales;f__Moraxellaceae;g__Acinetobacter*	0.0151	0.0996	0.0816	0.0018	0.0048	0.0151
*p__Proteobacteria;o__Rhizobiales;f__Bradyrhizobiaceae;g__Bradyrhizobium*	0.0064	0.0029	0.0078	0.0034	0.0035	0.0113
*p__Proteobacteria;o__Enterobacteriales;f__Enterobacteriaceae;g__Escherichia*	0.0018	0.0104	0.0229	0.0055	0.0058	0.0071
*p__Firmicutes;o__Clostridiales;f__Clostridiaceae;g__Clostridium*	0.0025	0.0247	0.0047	0.0009	0.0014	0.0007
*p__Proteobacteria;o__Burkholderiales;f__Comamonadaceae;g__Variovorax*	0.0060	0.0047	0.0083	0.0029	0.0072	0.0017
*p__Firmicutes;o__Clostridiales;f__;g__*	0.0025	0.0247	0.0047	0.0020	0.0019	0.0009
*p__Firmicutes;o__Turicibacterales;f__Turicibacteraceae;g__Turicibacter*	0.0009	0.0049	0.0016	0.0013	0.0002	0.0015

**Control**, non-treated piglets born to non-treated sows; **TS**, piglets born to treated sows; **TP**, piglets treated with ceftiofur born to non-treated sows

At D21, the number of differential ASVs was higher when comparing the control group with TS (92 ASVs) than with TP (68 ASVs). Among the ASVs affected by the antibiotic treatment, we found an ASV from *Mycoplasma* genus that was decreased in TS and absent in TP. This relative decrease in *Mycoplasma* in TP group was assigned, at least partially, to *M. hyorhinis* by PCR (Additional file 3: Table S2). Differentially abundant ASVs among the three groups (TS, TP and control) belonged to nasal-associated taxa, such as *Streptococcus* (more relatively abundant in TP), *Moraxella* and *Glaesserella* (both more relatively abundant in the control group). Interestingly, the same evidence was not observed at the genus level, suggesting that the antibiotic treatment in sows or piglets selected specific strains in each case. Within *Glaesserella genus, G. parasuis* is the only known member of the swine microbiota, for which a virulence-specific PCRs is available. We observed different colonization dynamics by virulent or non-virulent strains in the three groups, especially at early time points (Additional file 3: Table S2). The differences in ASVs were also supported by the isolation of colonies with a different fingerprinting profile in ERIC-PCR in the different groups. At genus level, not significant differences were detected, suggesting that the distribution of the different ASVs compensate each other yielding similar relative abundances in the groups. At D49, a global increase in the number and abundance of ASV was evident in all groups (as indicated in alpha diversity) and corresponded mostly to ASVs from *Bacteroidales* (9.40% in TS, 13.01% in TP and 12.56% in control) and *Clostridiales* (23.79% in TS, 21.54% in TP and 40.63% in control). At the end of nursery 593 ASVs were differentially abundant among the three groups (Additional file 4: Table S3). At genus level, some differentially abundant genera showed to be group-specific, such as *Mycoplasma,* whose abundance was higher in the control group (1.28% vs. 0.15% in TP and 0.33% in TS). In agreement, higher prevalence of *M. hyorhinis* was detected in the control group by PCR (95% vs. 74% in TP and 66% in TS). Sixteen genera were differentially abundant comparing the three groups (TS, TP and control), including some nasal-associated commensals: *Actinobacillus* (higher in TP), *Bordetella* (higher in TS), *Lactobacillus* (higher in TP), *Staphylococcus* (higher in TS) and a genus from the *Pasteurellaceae* family (higher in TS) (Additional file 5: Table S4). Ceftiofur treatment did not affect the prevalence of *S. suis* as detected by PCR, with a prevalence of over 90% at D7 and 100% at weaning and onwards in the three groups.

### Inoculation of early colonizers of the upper respiratory tract modifies the nasal microbiota of the piglets

**Fig. 4:**
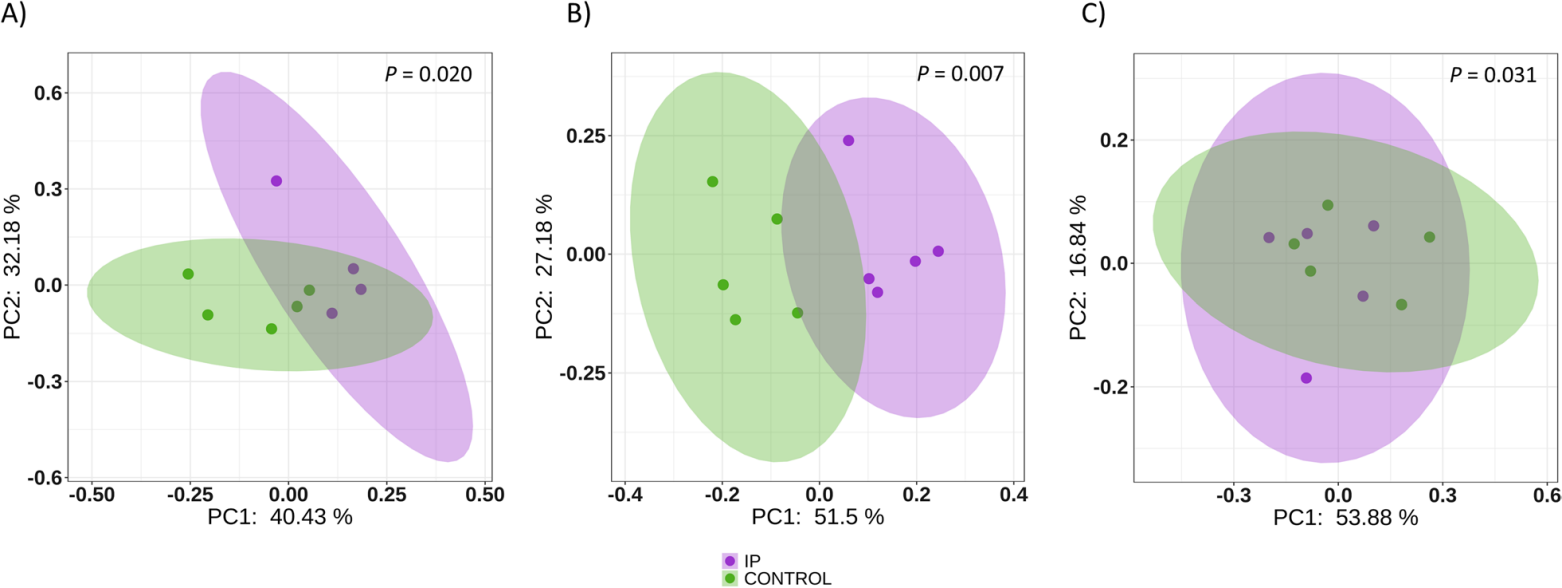
Beta diversity on weighted Unifrac distance matrices. PCoA was performed at D7 (**A**), D21 (**B**) and D49 (**C**). Purple dots represent samples from inoculated piglets with selected colonizers born to non-treated sows (IP) and green dots represent non-treated piglets born to non-treated sows (Control)

**Fig. 5:**
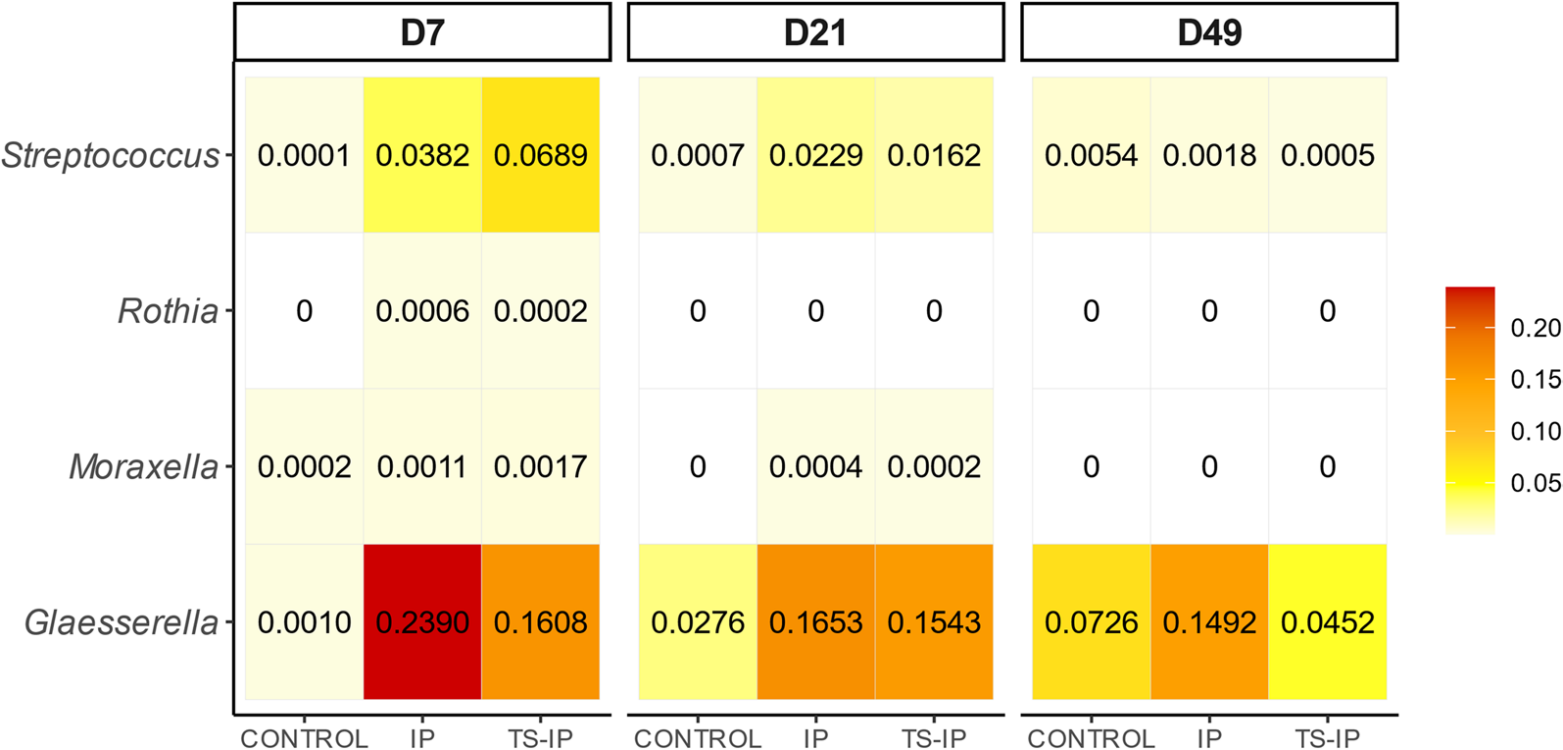
Heatmap representing the relative abundance (percentage) of the ASVs of the selected colonizers inoculated at birth in the inoculated (IP and TS-IP) and the control groups at D7, D21 and D49

Inoculation of non-treated piglets with selected colonizers (IP group) had an impact on their nasal microbiota. At D7, the mean alpha diversity measured by Shannon diversity index was higher in the inoculated IP piglets (Fig. 1) compared to the control group, although not significant at any timepoint (Kruskal–Wallis, *P* = 0.075, 0.14 and 0.25 for D7, D21 and D49, respectively). The microbiota composition estimated through beta diversity based on weighted Unifrac distance matrix was calculated for each timepoint (Fig. 4). Control and inoculated groups showed statistically different beta diversity at each timepoint (PERMANOVA, *P* = 0.02, *P* = 0.01 and *P* = 0.043 for D7, D21 and D49, respectively). The percentage of explanation attributed to the inoculation of the piglets (estimated through the Adonis function on the weighted Unifrac distance matrix) was 53% at D7, 44% at D21 and 36% at D49. Differential abundance analysis performed with ANCOM-BC methodology showed a total of 379 differential ASV, including the specific ASVs from the inoculated *Moraxella pluranimalium*, *Rothia nasimurium*, *Streptococcus pluranimalium* and *Glaesserella parasuis* strains which showed higher abundance in the IP group compared with the control group at D7 (Fig. 5), indicating that four out of five strains inoculated were able to colonize the nasal cavity of the piglets. The ASVs corresponding to the inoculated *Glaesserella*, *Streptococcus* and *Moraxella* were also present at weaning (D21), although significative differences were only detected with the *Streptococcus* ASV. These ASVs were not significantly different at the end of nursery where only *Glaesserella* and *Streptococcus* were detected at this latter timepoint. The inoculated *Glaesserella* strain was also recovered by culture at all the timepoints.

**Fig. 6:**
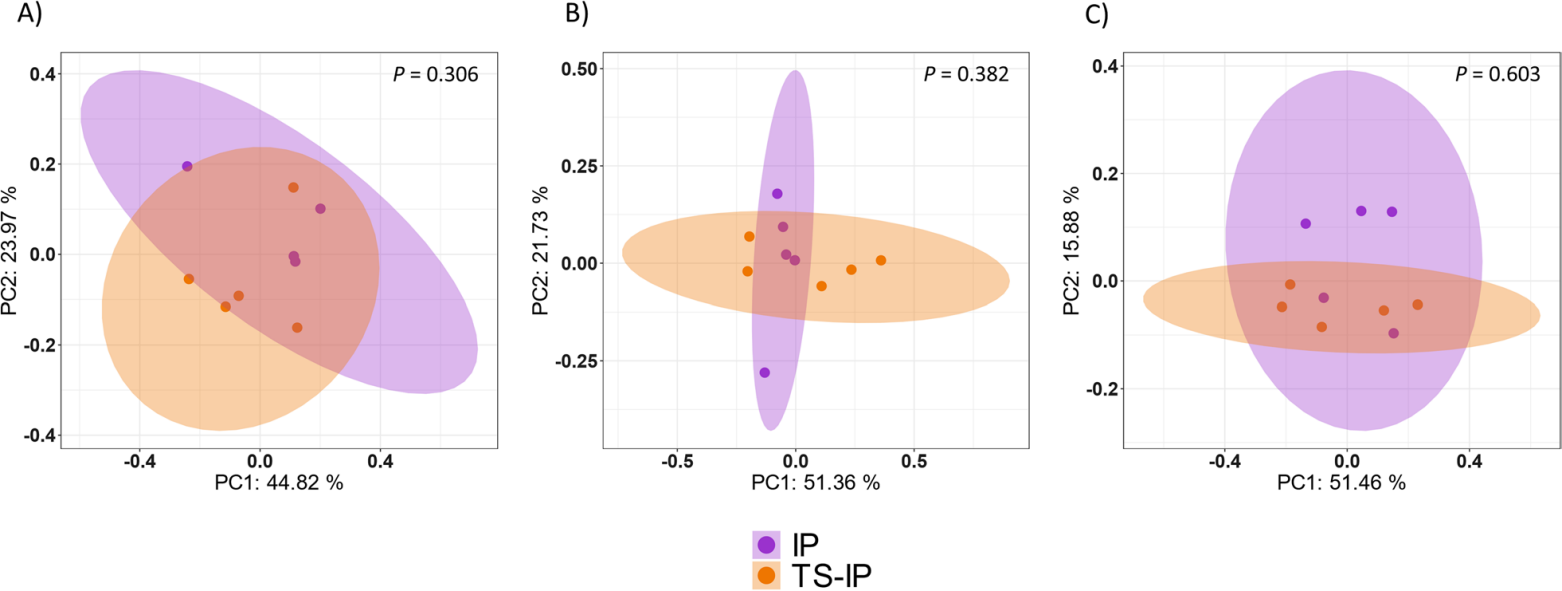
Beta diversity on weighted Unifrac distance matrices. PCoA was performed at D7 (**A**), D21 (**B**) and D49 (**C**). Purple dots represent samples from inoculated piglets with selected colonizers born to non-treated sows (IP) and dark orange dots represent inoculated piglets born to treated sows (TS-IP)

The colonization of the piglets by the selected colonizers seemed not to be favored by treating the sows with ceftiofur. When alpha diversity (measured through Shannon index) of inoculated piglets from treated or non-treated sows (IP vs TS-IP) was compared, no significant differences were detected at any of the time points analyzed (Kruskal Wallis, *P* > 0.05; Fig. 1). Differences in microbiota composition (beta diversity) were not detected with any of the metrics/distances analyzed (Jaccard, Bray Curtis and Unifrac; Fig. 6) at any of the sampling timepoints (IP vs. TS-IP; Bray Curtis PERMANOVA, *P* = 0.396). The nasal microbiota composition of the two inoculated groups was not different at D7, although it was different for both groups when compared to the control (PERMANOVA, TS-IP vs. control, *P* = 0.008 and IP vs. control, *P* = 0.013, at D7). ANCOM-BC analysis showed a total of 379 ASVs at D7, including the ASV of the inoculated *Rothia* (0.0006% in IP and 0.0002% in TS-IP, while absent in control group). Similar findings were observed at D21 (PERMANOVA, IP vs. TS-IP, *P* = 0.142; IP vs. control *P* = 0.011; TS-IP vs. control, *P* = 0.007) in beta diversity analysis, but at this timepoint none of the colonizers were differentially abundant. At the end of nursery (D49), inoculated groups were not significantly different (IP vs. TS-IP; Bray Curtis PERMANOVA, *P* = 0.062). At this timepoint, only *Glaesserella* and *Streptococcus* were detected in the samples, but no significant differences were found in the differential abundance analysis (ANCOM-BC). Inoculation of the selected nasal colonizers reduced the divergence of the microbiota composition produced by the treatment of the sows. The differences detected at D7 and D21 in beta diversity were absent at the end of the study, indicating TS-IP piglets had a similar composition to the control (*P* = 0.163, with all beta diversity metrics used, while piglets born to treated sows but not inoculated (TS) presented different composition than control at D49. The IP group also showed a different microbiota than control at D49 (*P* = 0.008). Interestingly, the inoculated colonizers were found in higher relative abundance in IP than in TS-IP.

### WGS metagenomics confirmed the differences observed in beta diversity between weaning and the end of nursery in all groups

**Fig. 7:**
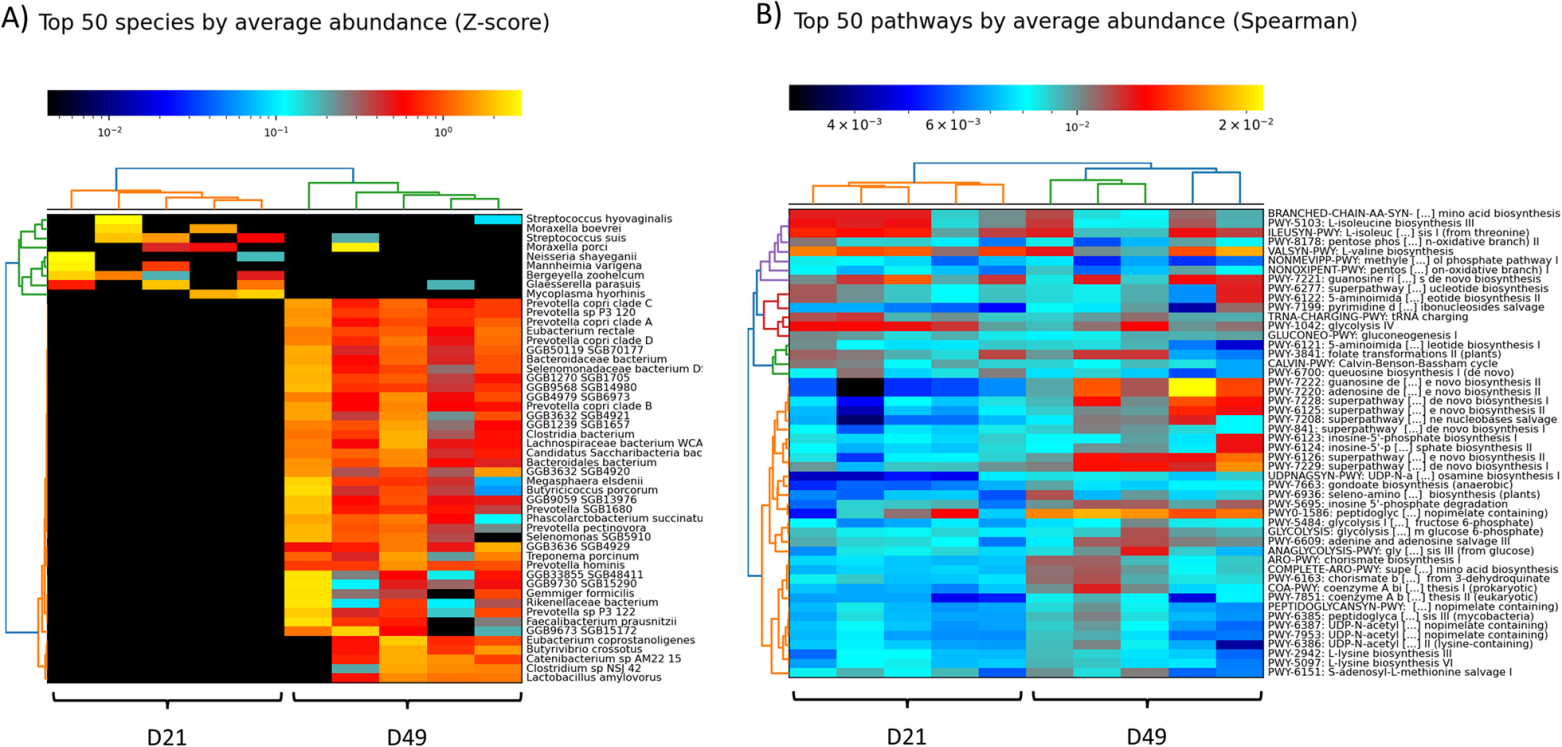
Heatmap representing the top 50 species by average abundance clustered with Z-score normalization in non-treated piglets born to non-treated sows (control) at D21 and D49 (**A**). Top 50 pathways by average abundance by Spearman correlation coefficient in control group at D21 and D49 (**B**)

A total of 143 species and 99 genera were identified in the nasal microbiota samples from D21 and D49. In the control group, we identified a total of 10 genera and 14 species at weaning (D21), and the composition of the nasal microbiota was clearly modified through the incorporation of other members at D49 (Fig. 7A Top 50 species z-score control group D21 y D49). The divergence between D21 and D49 was also observed in piglets from the rest of the groups in a clustering analysis by average abundance (Z-score) at different taxonomic levels, including species (Additional file 6: Fig. S2). At D21, the top 10 most abundant genera in all groups were *Glaesserella*, *Moraxella*, *Bergeyella*, *Mycoplasma*, *Streptococcus*, *Pasteurella*, *Neisseria*, *Mannheimia*, *Lactobacillus* and *Acinetobacter*, which belong to taxa commonly associated to nasal microbiota. At the end of the nursery (D49), a plethora of species from taxa classically associated to gut microbiota and absent at weaning was detected. In inoculated piglets, shotgun metagenomic data for the inoculated *Glaesserella, Moraxella* and *Streptococcus* agreed with the 16S sequencing data. *Rothia* was not detected at weaning, but on the other hand, it was detected at D49 by shotgun sequencing*,* contrary to the 16S data.

Several species within *Archaea* domain were affected by the antibiotic treatment. While they were not found in any group at weaning, at the end of the nursery they were found more relatively abundant in the control and the IP group than the piglets born to treated sows (TS and TS-IP), although in less than one percent in all groups. *Archaea* were absent in the TP group at this latter timepoint as well.

A total of 314 pathways and 1580 enzymes were found considering all samples. Most of the pathways were present in all groups (Additional file 7: Fig. S3), except TS group which showed several specific pathways at D21, although represented in very low relative abundance. The differences found at taxa level between weaning and the end of nursery were also found in the functional profiling but were not so remarkable (Fig. 7B and Additional file 6: Fig. S2).

In the control group, 12 significantly different pathways were detected (through multivariable association analysis with linear model) when compared longitudinally (between D21 and D49 timepoints).These pathways were not among the top-50 most abundant pathways, and included several pathways in relative higher abundance at D21 (peptidoglycan maturation, heme b biosynthesis II, and two lipid IVA -precursor of lipid A- biosynthesis pathways) and others with higher abundance at D49 (anaerobic degradation of acetylene, degradation of stachyose, degradation of fucose and rhamnose, incomplete reductive TCA cycle, de novo biosynthesis of NAD from aspartate, L-arginine biosynthesis and two pathways of biosynthesis of pyrimidine).

Focusing on differences due to the antibiotic treatment at each time point, we detected few differential pathways, which were in higher abundance in the TS group (L-glutamate and L-glutamine biosynthesis at D21 but also at D49, superpathway of anaerobic sucrose degradation at D21, glycolysis-TCA-Glyox-bypass: superpathway of glycolysis at D49, and L-glutamine biosynthesis II at D21) when compared to TP and control groups.

In inoculated groups, we observed several pathways differentially increased at D21. Interestingly, these pathways increased in all groups at D49, indicating that these functions appeared earlier due to intervention, such as stachyose degradation, NAD de novo biosynthesis from aspartate, dTDP-beta-L-rhamnose biosynthesis (Additional file 8: Fig. S4). On the other hand, three pathways were found at lower abundance in both inoculated groups at D21 when compared to the control group, showing a delay in their natural appearance: superpathway of branched chain aminoacid, L-isoleucine biosynthesis I (from threonine) and L-isoleucine biosynthesis III. In addition, some differences were specifically observed in each inoculated group. The IP group showed two differentially abundant pathways: one in higher abundance at D49 (chitin derivatives degradation), and a second with lower abundance also at D49 (beta-(1,4)-mannan degradation). In TS-IP group, we observed five pathways that were more abundant at D21: incomplete reductive TCA cycle, NAD salvage pathway II (PNC IV cycle), 4-deoxy-L-threo-hex-4-enopyranuronate degradation, superpathway of arginine and polyamine biosynthesis, and purine nucleobases degradation II (anaerobic), where the latter three pathways appeared in lower relative abundance at D49 in the same group TS-IP (Additional file 8: Fig. S4).

The divergence observed in the taxonomic profiles among the groups was not so evident at the functional level, suggesting that different microbial communities may be responsible for similar functions.

### A higher number of beta-lactamase genes were detected in antibiotic-treated piglets

**Fig. 8:**
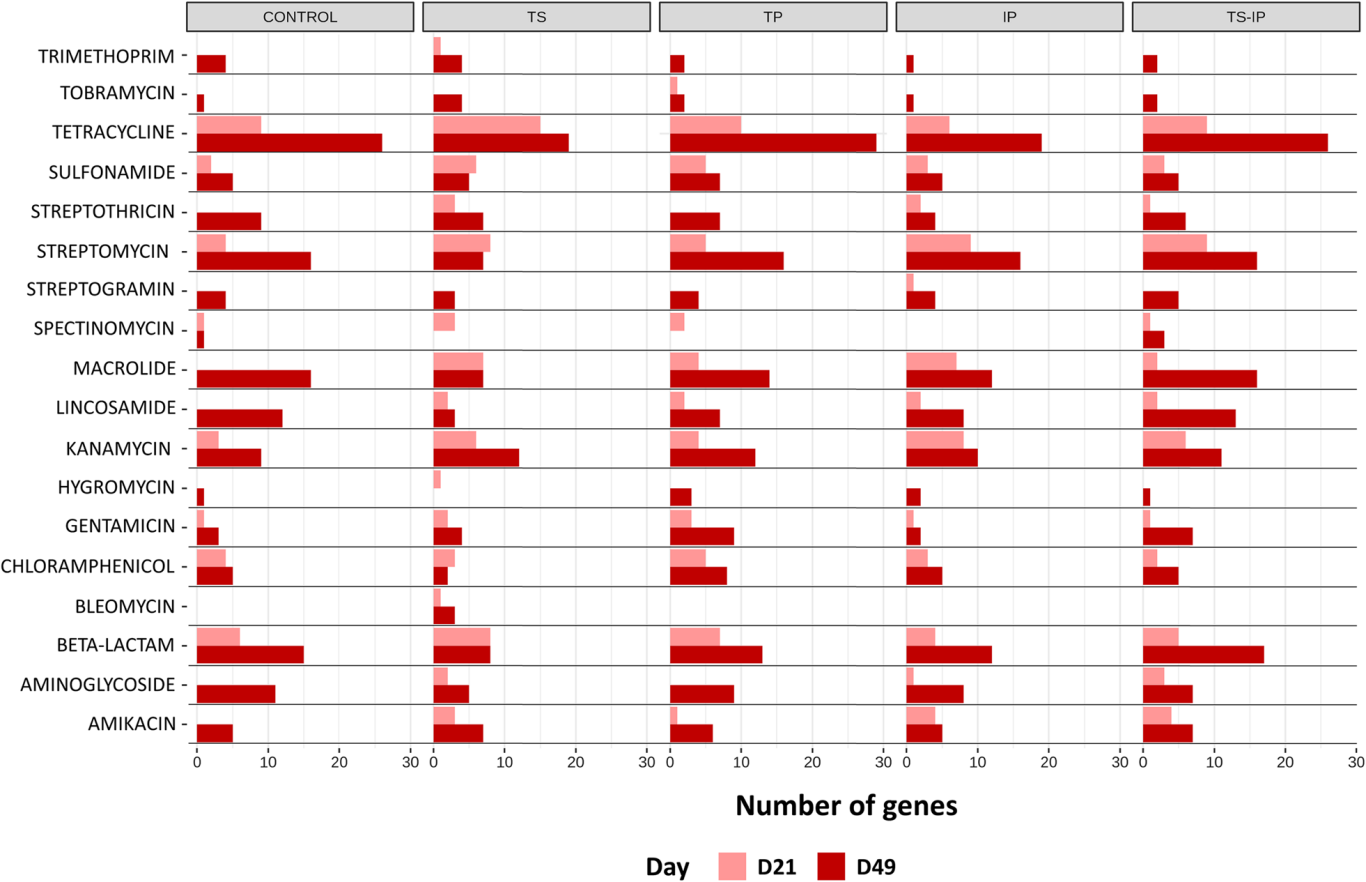
Number of genes associated with the different antibiotic classes found in the MAGs in the different groups and timepoints (D21 and D49). Pregnant sows were treated with ceftiofur or remained untreated. A group of piglets born to treated sows remained nontreated (TS group), while piglets born to non-treated sows were treated with ceftiofur (TP group). A group of piglets was inoculated with selected colonizers of the upper respiratory tract either born to ceftiofur-treated sows (TS-IP) and to non-treated sows (IP group). As control, piglets born to non-treated sows remained non-treated

The genes involved in antibiotic resistances (resistome) were predicted using the AMRFinderPlus database by comparing the MAGs present in each group at D21 and D49. For each group and timepoint, the total number of genes associated to AMR found in each group is listed in Additional file 9: Table S6. Globally, the number of unique resistance genes increased significantly over time in all groups except TS. This TS group showed the highest number of unique resistance genes at D21 (31 genes from 17 families), a number significantly higher than the control (Fisher test with Bonferroni correction, *P* = 0.026), which presented the lowest number at D21 (14 genes from 9 families). The number of unique AMR genes after the inoculation of colonizers was not different from the control group at D21 or D49. The counts of genes from the predicted resistome belonging to different antibiotic families is represented in Fig. 8.

**Fig. 9:**
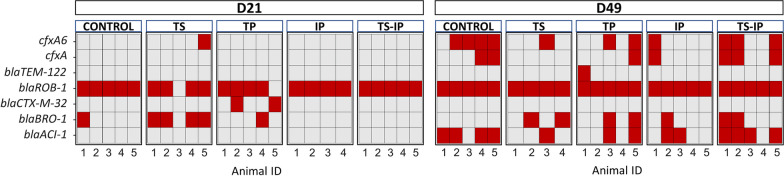
Beta-lactam genes presence (in red) individually in piglets by group and timepoint (D21 and D49). Pregnant sows were treated with ceftiofur or remained untreated. A group of piglets born to treated sows remained nontreated (TS group), while piglets born to non-treated sows were treated with ceftiofur (TP group). A group of piglets were inoculated with selected colonizers of the upper respiratory tract either born to ceftiofur-treated sows (TS-IP) and to non-treated sows (IP group). As control, piglets born to non-treated sows remained non-treated

Since ceftiofur was the antibiotic administered in this study, we focused on the genes related to this resistance. In general, we detected 7 genes for class-A beta-lactamases: *cfxA* and *cfxA6* (broad spectrum), *blaTEM-122* (inhibitor-resistant broad spectrum), *blaROB-1* (cephalosporin hydrolyzing enzyme), *blaACI-1* and *blaCTX-M-32* (extended spectrum) and *blaBRO-1* (Fig. 9). The number of these genes increased with age in all groups. No major differences were detected among the distribution of these genes between the groups, except for *blaCTX-M-32* and *blaTEM-122*, which were found only in the TP group at D21 and D49, respectively.

## Discussion

This study was motivated by two main questions, firstly to assess the impact of the ceftiofur treatment either in sows or piglets over time on their nasal microbiota and secondly, to explore the possibility of modify it by inoculation at birth of natural colonizers selected from healthy piglets. The administration of ceftiofur had a longer impact on the nasal microbiota composition of piglets when administered to their sows before farrowing than directly applied to the piglets at birth. The effect of ceftiofur on nasal microbiota composition was partially reverted by the inoculation of nasal colonizers to newborn piglets and was accompanied by an improvement in piglet health. In addition, the prevalence of antibiotic resistance genes increased over time in all groups, being higher at weaning in the group of piglets born to treated sows. The selection of more beta-lactam resistance genes (*blaCTX-M32* and *blaTEM-122*) was also observed when ceftiofur was administered directly to the piglets. Of note, our findings may be farm specific since one of the major factors involved in shaping the microbiota of the animals is the environment [6, 61, 62]. However, the strength of this study relies in the number of animal and the longitudinal approach to study the nasal microbiota, together with the two sequencing techniques used at a high sequencing depth. In agreement with Pirolo et al. [63], we found less diversity in the samples sequenced by WGS metagenomics than by amplicon sequencing, although the specific results are not completely comparable due to the different study design (age of the animals, sampling technique, bioinformatic analysis, etc.).

The nasal microbiota composition of the followed-up piglets switched from a clear predominance of *Firmicutes*, *Proteobacteria* and *Bacteroidetes* at weaning to a microbiota composition with an important contribution of *Clostridiales* and other taxa classically found in gastrointestinal tract [62]. This can be explained by the change from milk to solid feed [62] and the natural rooting behavior of the piglets that put the nostrils in contact with fecal material [64]. In the postweaning barn, the higher animal density worsens the air quality (increased levels of ammonia and dust), probably contributing to an inflammation state in the nose allowing the presence of anaerobic taxa. In fact, the increased presence of anaerobic bacteria in the human sinus correlates with inflammation and chronic diseases related to failure in immune system priming by nasopharyngeal microbiota [65]. These changes underline the importance to conduct longitudinal studies to understand the evolution of the nasal microbiota over time. Although the differences observed in composition seemed to be reduced at functional level, the specific pathways that were modulated by the treatment and/or the inoculation of colonizers deserve further studies to understand the implications in microbiota functionality. It would be also important to determine which member of the microbiota is implicated in health improvement in order to be able to design rational interventions.

As expected, ceftiofur treatment affected the microbiota composition. However, early after administration, the antibiotic induced an unforeseen increase in the diversity of the piglets’ nasal microbiota. This finding confronts many studies that have shown that antibiotic administration reduces bacterial diversity in different ecological niches [21, 66–68]. However, this was a transitory increment in the diversity derived from the presence of a plethora of bacterial taxa that are not consistently described in the respiratory microbiota of suids, whose presence can be considered as dysbiotic. These intrusive bacteria belonged mainly to taxa that could be traced back to the environment and were probably occupying the space available on the nasal mucosa after the drastic reduction of the nasal-associated bacteria, caused by the antibiotic treatment. Contrary to the digestive tract, which has several barriers to avoid contamination with foreign bacteria, such as the acidic pH of the stomach, the nasal cavity is directly open to the exterior and this may facilitate temporal colonization by environmental bacteria when the diversity of the resident microbiota is reduced.

In the long term, our data showed that the effect of the antibiotic treatment lasted longer when administered to the sows compared to the direct administration to the piglets. This finding is in agreement with other studies where the sow contact with the newborn piglets was reported as one of the major drivers in the nasal microbiota composition [8]. The treatment of the sows probably reduced the bacterial load in their microbiota, consequently reducing the possibility of bacterial transmission to the piglets. Management of the sow seems to have an important impact on the piglet’s nasal microbiota at least during the first weeks of age when the microbiota is still not stable [62], as previously observed when vaccination performed on sows modified the nasal microbiota of piglets [69]. Interestingly, inoculation of nasal colonizers reverted the effect of the ceftiofur administration in sows, reducing the fluctuations in diversity and modulating the microbiota towards a more stable scenario. Inoculation delayed the appearance of virulent strains of *G. parasuis* and we detected different dynamics of the prevalence of *G. parasuis*, *S. suis* and *M. hyorhinis* strains, but it is difficult to correlate these changes to the clinical status. Nevertheless, the group of piglets born to treated sows that were inoculated with colonizers showed better clinical status after weaning. It is tempting to hypothetise that the sow treatment resulted in a reduction of the transmission of pathogens from the sows to the piglets, which may have been then replaced by the inoculated natural members of the microbiota. In humans, the impact of antibiotic administration before delivery has been also associated with alteration in the infant’s microbiota and health [70, 71]. Our findings suggest that the inoculation of potential beneficial colonizers may open a strategy to promote newborn health in case the mother needs to be treated. In fact, some studies have tested nasal probiotics against respiratory infections in broilers and humans with positive results [24, 26]. However, the strains used in this study may not be the optimal colonizers and microbiota intervention may be improved by choosing other strains with better colonization capacities.

In the present study, ceftiofur treatment by itself, either of the sows or the piglets, did not improve the health status or the productivity of the piglets, supporting that metaphylactic treatments can be avoided without deleterious effects on production. In addition, we have observed that, with age, piglets showed an increasing number of antibiotic resistances, in agreement with previous reports [72]. In the current scenario of antimicrobial reduction in farms, the manipulation of the microbiota to maintain animal health appears as a promising strategy. Here, we have demonstrated that modification of the piglets’ nasal microbiota by the inoculation of natural colonizers is possible. However, it would be essential to select natural colonizers free of antimicrobial resistances, especially those in mobile elements that could be easily transferable. Interestingly, the inoculated bacteria seemed to colonize better in piglets born to non-treated sows, probably due to some beneficial interactions with other members of the nasal microbiota.

## Conclusions

This study shed light on the influence of the antibiotic treatment on the piglets’ nasal microbiota over time. We have demonstrated that ceftiofur treatment has a longer effect on the piglet’s nasal microbiota when it is administered to the sow than directly to the piglet. Moreover, the effects of the sow antibiotic treatment on piglet's nasal microbiota were partially reverted by inoculating a pool of nasal colonizers. This might represent a strategy to improve pig’s health by using a non-invasive alternative to antibiotics.

### Supplementary Information

**Additional file 1**. **Figure S1.** Beta diversity on weighted Unifrac distance matrices. PCoA was performed at D7 (A), D21 (B) and D49 (C). Blue dots represent samples from treated piglets born to non-treated sows (TP) and orange dots represent non-treated piglets born to treated sows (TS).

**Additional file 2**. **Table S1.** Statistical results from differential abundance analysis at ASV level of the TP, TS and control groups at D7 and D21 (ANCOM-BC). Each comparison performed is indicated in the column header and includes a column with the W value and a second column with the significance.

**Additional file 3**.** Table S2.** Results of the detection by PCRs of the query pathogens (*G. parasuis*, *M. hyorhinis* and *S. suis*) in the different study groups at D7, D15, D21, and D49.

**Additional file 4**. **Table S3.** Statistical results from differential abundance analysis at ASV level of the TP, TS and control groups at D49 (ANCOM-BC). Each comparison performed is indicated in the column header and includes a column with the W value and a second column with the significance.

**Additional file 5**. **Table S4.** Statistical results from differential abundance analysis at genus level of the TP, TS and control groups at D49 (ANCOM-BC). Each comparison performed is indicated in the column header and includes a column with the W value and a second column with the significance.

**Additional file 6**. **Figure S2. **Heatmap representing the top 25 species by average abundance with Bray Curtis coefficient (A and C) and top 25 pathways by average abundance by Spearman correlation coefficient (B and D). Pregnant sows were treated with ceftiofur or remained untreated. A group of piglets born to treated sows remained nontreated (TS group), while piglets born to non-treated sows were treated with ceftiofur (TP group). A group of piglets were inoculated with selected colonizers of the upper respiratory tract either born to ceftiofur-treated sows (TS-IP) and to non-treated sows (IP group). As control, piglets born to non-treated sows remained non-treated. Groups TS, TP and control groups are compared in panels A and B. Groups IP, TS-IP and control are compared in panels C and D.

**Additional file 7**. **Figure S3.** Venn diagrams representing the pathways presence and absence at weaning (A) and the end of nursery (B) in the different study groups. Pregnant sows were treated with ceftiofur or remained untreated. A group of piglets born to treated sows remained nontreated (TS group), while piglets born to non-treated sows were treated with ceftiofur (TP group). A group of piglets were inoculated with selected colonizers of the upper respiratory tract either born to ceftiofur-treated sows (TS-IP) and to non-treated sows (IP group). As control, piglets born to non-treated sows remained non-treated.

**Additional file 8**. **Figure S4.** Relative abundance of the significant pathways found in inoculated piglets. Pregnant sows were treated with ceftiofur or remained untreated. Ppiglets were inoculated with selected colonizers of the upper respiratory tract either born to ceftiofur-treated sows (TS-IP) or to non-treated sows (IP group). As control, piglets born to non-treated sows remained non-treated and non-inoculated. Sampling time points were 21 days of age (D21, weaning) and 49 days of age (D49, end of nursery).

**Additional file 9**. **Table S5.** Total number of AMR genes, unique genes and gene families at D21 and D49 in the different study groups. Pregnant sows were treated with ceftiofur or remained untreated. A group of piglets born to treated sows remained nontreated (TS group), while piglets born to non-treated sows were treated with ceftiofur (TP group). A group of piglets were inoculated with selected colonizers of the upper respiratory tract either born to ceftiofur-treated sows (TS-IP) and to non-treated sows (IP group). As control, piglets born to non-treated sows remained non-treated.

## Data Availability

The sequencing data produced for this study are available in the Sequence Read Archive (SRA) under accession number; Bioproject PRJNA954430.

## Abbreviations

ASV(s): Amplicon sequence variant(s)
ADWG: Average daily weight gain
TS: Treated sow
NTS: Non-treated sow
TP: Treated piglet
IP: Inoculated piglet
WGS: Whole genome shotgun
MAG: Metagenome assembled genome
AMR: Antimicrobial resistance
ANCOM-BC: Analysis of compositions of microbiomes with bias correction
