# Supplementary figures and images for: Ceftiofur treatment of sows results in long-term alterations in the nasal microbiota of the offspring that can be ameliorated by inoculation of nasal colonizers
Source: Anim Microbiome. 2023 Oct 20;5:53. doi: 10.1186/s42523-023-00275-3 (PMC10588210; doi:10.1186/s42523-023-00275-3)

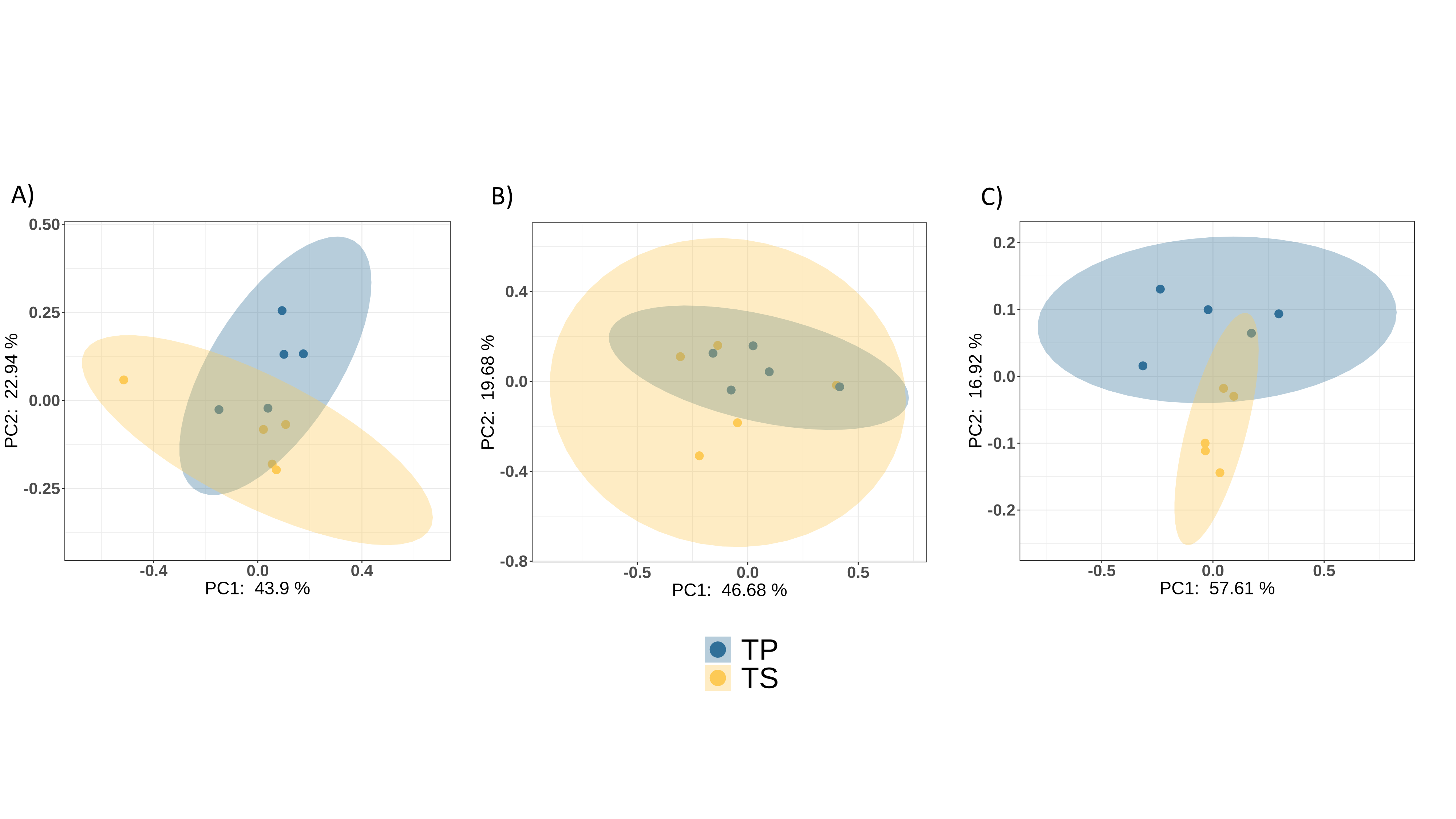

Supplement: Supplementary file 1 — Additional file 1. Figure S1. Beta diversity on weighted Unifrac distance matrices. PCoA was performed at D7 (A), D21 (B) and D49 (C). Blue dots represent samples from treated piglets born to non-treated sows (TP) and orange dots represent non-treated piglets born to treated sows (TS). [file 42523_2023_275_MOESM1_ESM.tif]

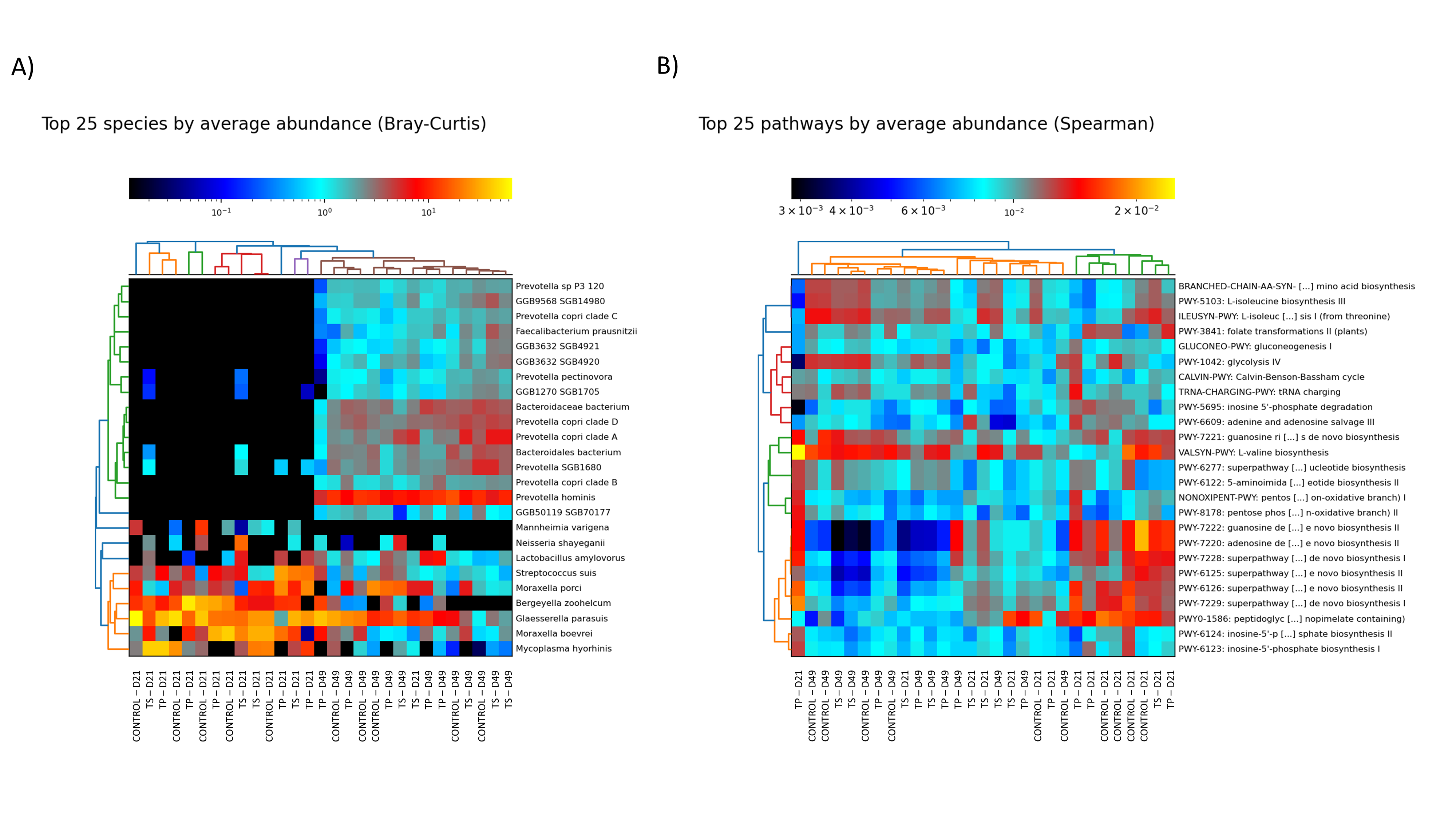

Supplement: Supplementary file 6 — Additional file 6. Figure S2. Heatmap representing the top 25 species by average abundance with Bray Curtis coefficient (A and C) and top 25 pathways by average abundance by Spearman correlation coefficient (B and D). Pregnant sows were treated with ceftiofur or remained untreated. A group of piglets born to treated sows remained nontreated (TS group), while piglets born to non-treated sows were treated with ceftiofur (TP group). A group of piglets were inoculated with selected colonizers of the upper respiratory tract either born to ceftiofur-treated sows (TS-IP) and to non-treated sows (IP group). As control, piglets born to non-treated sows remained non-treated. Groups TS, TP and control groups are compared in panels A and B. Groups IP, TS-IP and control are compared in panels C and D. [file 42523_2023_275_MOESM6_ESM.zip › 42523_2023_275_MOESM6_ESM/Figure S2 A-B.TIF]

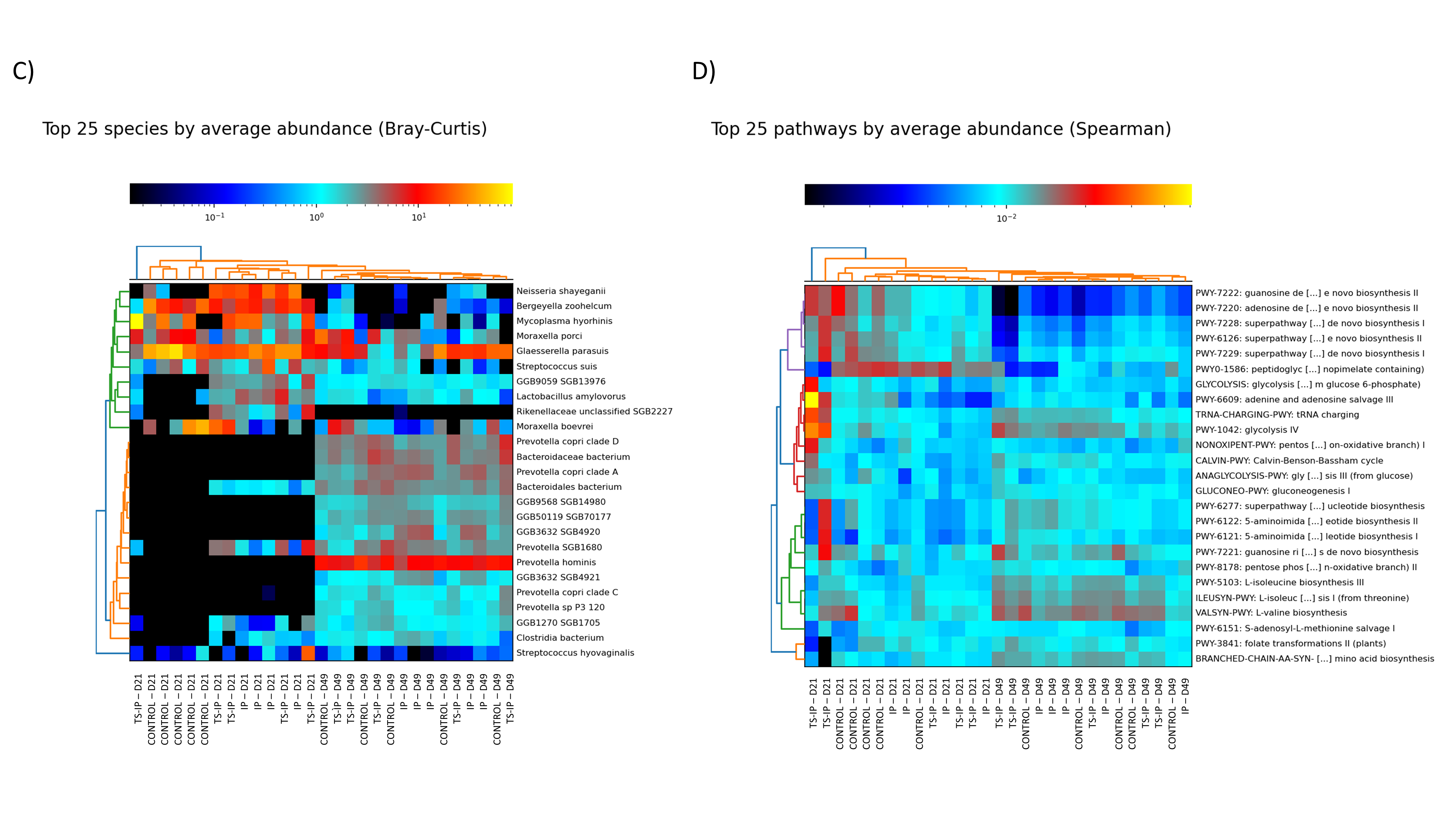

Supplement: Supplementary file 6 — Additional file 6. Figure S2. Heatmap representing the top 25 species by average abundance with Bray Curtis coefficient (A and C) and top 25 pathways by average abundance by Spearman correlation coefficient (B and D). Pregnant sows were treated with ceftiofur or remained untreated. A group of piglets born to treated sows remained nontreated (TS group), while piglets born to non-treated sows were treated with ceftiofur (TP group). A group of piglets were inoculated with selected colonizers of the upper respiratory tract either born to ceftiofur-treated sows (TS-IP) and to non-treated sows (IP group). As control, piglets born to non-treated sows remained non-treated. Groups TS, TP and control groups are compared in panels A and B. Groups IP, TS-IP and control are compared in panels C and D. [file 42523_2023_275_MOESM6_ESM.zip › 42523_2023_275_MOESM6_ESM/Figure S2 C-D.TIF]

# PATHWAYS

A)

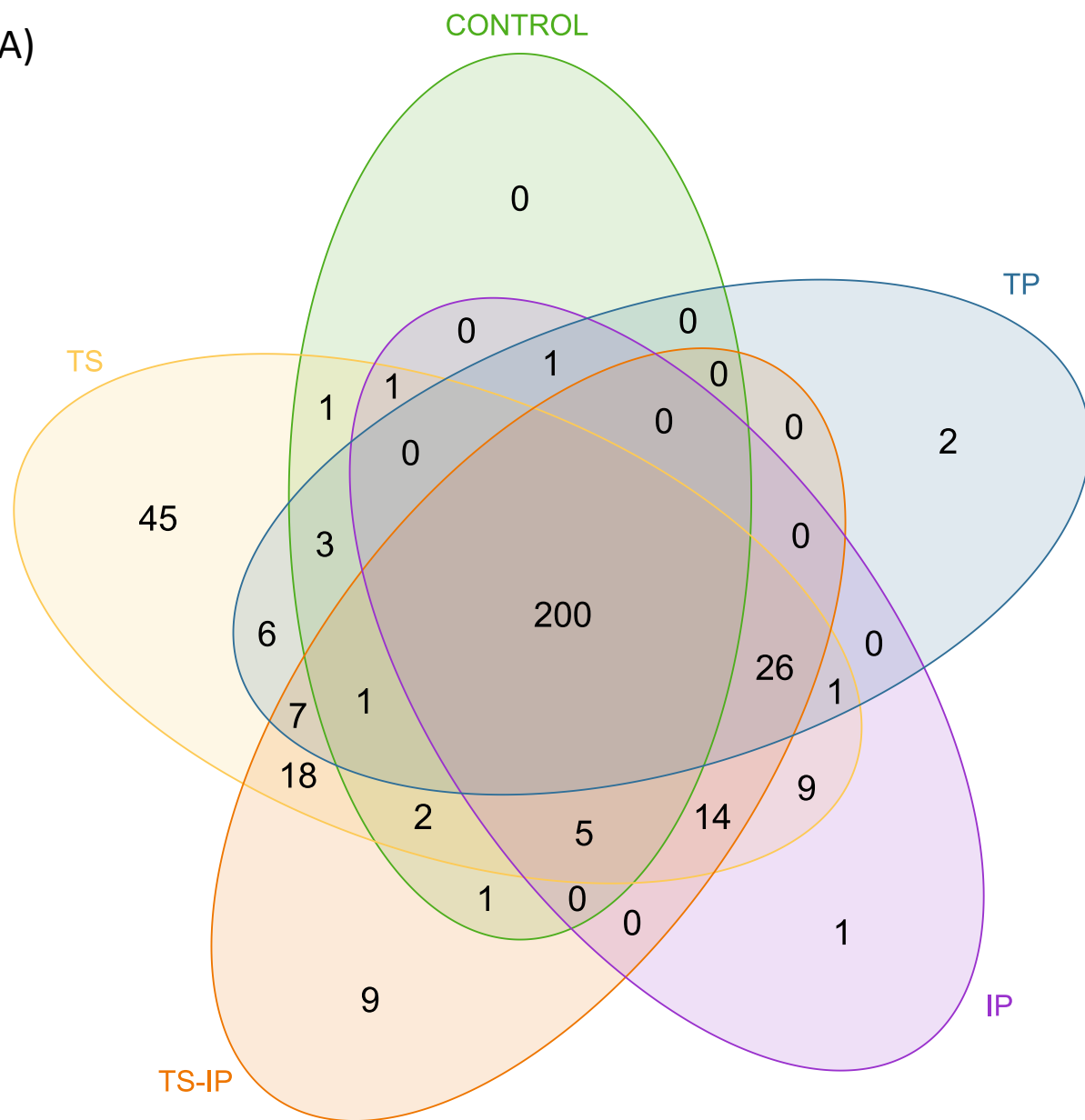

B)

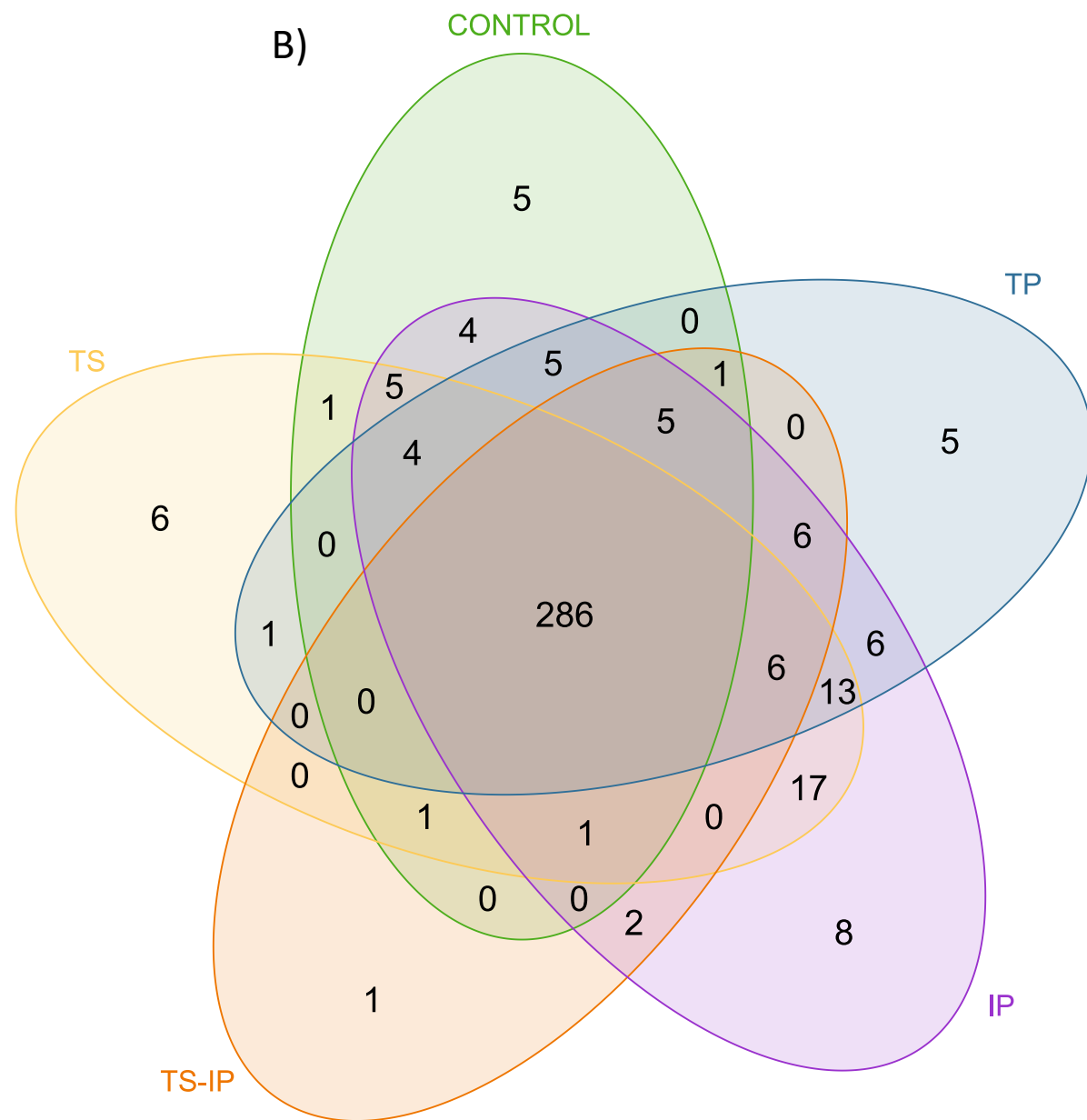

Supplement: Supplementary file 7 — Additional file 7. Figure S3. Venn diagrams representing the pathways presence and absence at weaning (A) and the end of nursery (B) in the different study groups. Pregnant sows were treated with ceftiofur or remained untreated. A group of piglets born to treated sows remained nontreated (TS group), while piglets born to non-treated sows were treated with ceftiofur (TP group). A group of piglets were inoculated with selected colonizers of the upper respiratory tract either born to ceftiofur-treated sows (TS-IP) and to non-treated sows (IP group). As control, piglets born to non-treated sows remained non-treated. [file 42523_2023_275_MOESM7_ESM.pdf]
