# Supplementary material for: Ceftiofur treatment of sows results in long-term alterations in the nasal microbiota of the offspring that can be ameliorated by inoculation of nasal colonizers
Source: Anim Microbiome. 2023 Oct 20;5:53. doi: 10.1186/s42523-023-00275-3 (PMC10588210; doi:10.1186/s42523-023-00275-3)
Supplement: Supplementary file 8 — Additional file 8. Figure S4. Relative abundance of the significant pathways found in inoculated piglets. Pregnant sows were treated with ceftiofur or remained untreated. Ppiglets were inoculated with selected colonizers of the upper respiratory tract either born to ceftiofur-treated sows (TS-IP) or to non-treated sows (IP group). As control, piglets born to non-treated sows remained non-treated and non-inoculated. Sampling time points were 21 days of age (D21, weaning) and 49 days of age (D49, end of nursery). [file 42523_2023_275_MOESM8_ESM.pdf]

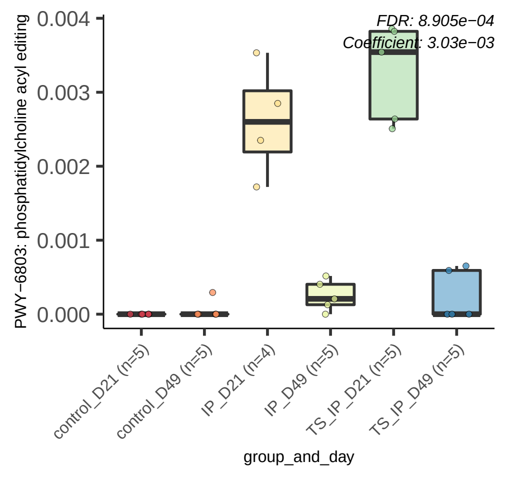
FDR: 8.905e−04
Coefficient: 3.03e−03
PWY−6803: phosphatidylcholine acyl editing
0.004
0.003
0.002
0.001
0.000
control_D21 (n=5)
control_D49 (n=5)
IP_D21 (n=4)
IP_D49 (n=5)
TS_IP_D21 (n=5)
TS_IP_D49 (n=5)
group_and_day

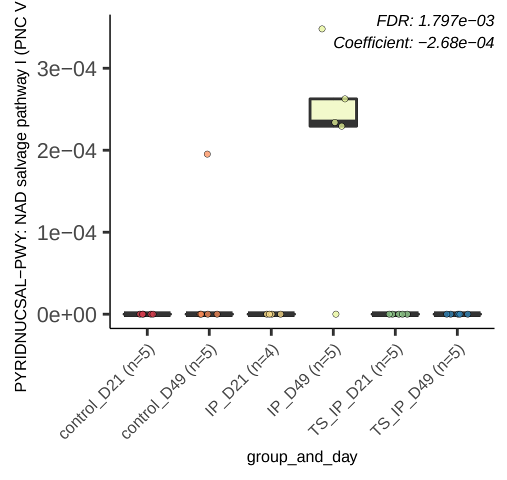

FDR: 1.797e−03
Coefficient: −2.68e−04
PYRIDNUCSAL−PWY: NAD salvage pathway I (PNC V
3e−04
2e−04
1e−04
0e+00
control_D21 (n=5)
control_D49 (n=5)
IP_D21 (n=4)
IP_D49 (n=5)
TS_IP_D21 (n=5)
TS_IP_D49 (n=5)
group_and_day

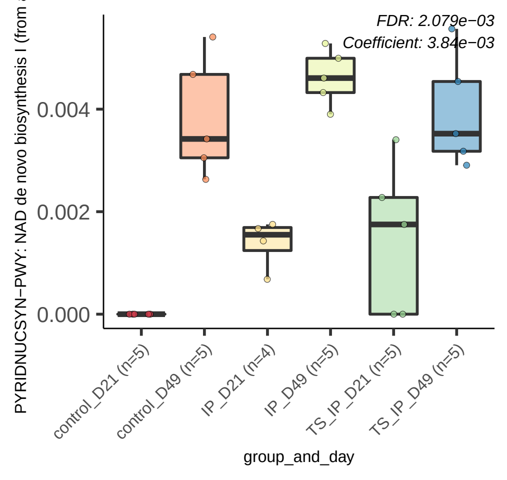
FDR: 2.079e−03
Coefficient: 3.84e−03
PYRIDNUCSYN−PWY: NAD de novo biosynthesis I (from a
0.000
0.002
0.004
control_D21 (n=5)
control_D49 (n=5)
IP_D21 (n=4)
IP_D49 (n=5)
TS_IP_D21 (n=5)
TS_IP_D49 (n=5)
group_and_day

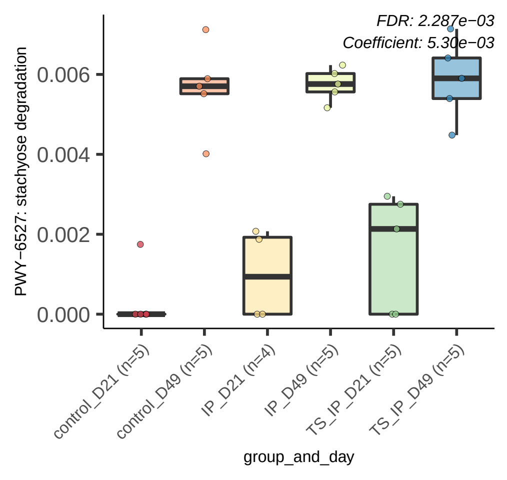

FDR: 2.287e−03
Coefficient: 5.30e−03
PWY−6527: stachyose degradation
0.000
0.002
0.004
0.006
control_D21 (n=5)
control_D49 (n=5)
IP_D21 (n=4)
IP_D49 (n=5)
TS_IP_D21 (n=5)
TS_IP_D49 (n=5)
group_and_day

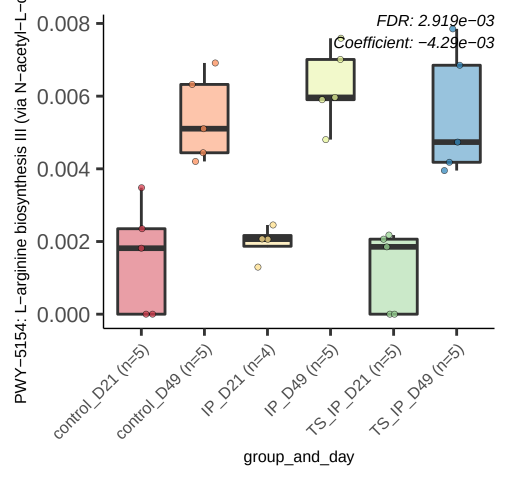
FDR: 2.919e−03
Coefficient: −4.29e−03
PWY−5154: L−arginine biosynthesis III (via N−acetyl−L−c
0.008
0.006
0.004
0.002
0.000
control_D21 (n=5)
control_D49 (n=5)
IP_D21 (n=4)
IP_D49 (n=5)
TS_IP_D21 (n=5)
TS_IP_D49 (n=5)
group_and_day

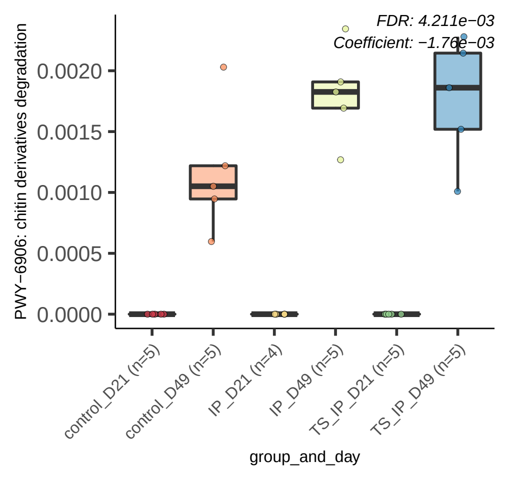
FDR: 4.211e−03
Coefficient: −1.76e−03
PWY−6906: chitin derivatives degradation
0.0020
0.0015
0.0010
0.0005
0.0000
control_D21 (n=5)
control_D49 (n=5)
IP_D21 (n=4)
IP_D49 (n=5)
TS_IP_D21 (n=5)
TS_IP_D49 (n=5)
group_and_day

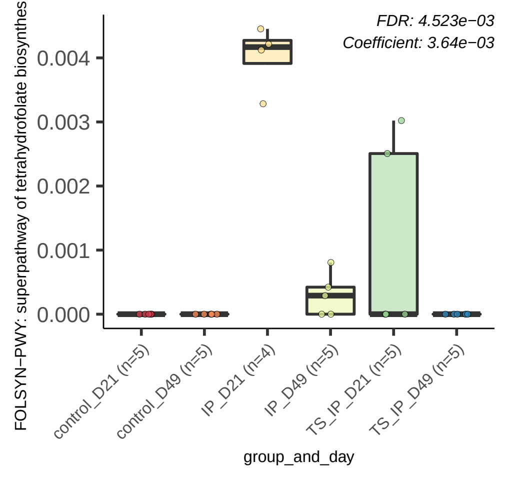

FDR: 4.523e−03
Coefficient: 3.64e−03
FOLSYN−PWY: superpathway of tetrahydrofolate biosynthes
0.000
0.001
0.002
0.003
0.004
control_D21 (n=5)
control_D49 (n=5)
IP_D21 (n=4)
IP_D49 (n=5)
TS_IP_D21 (n=5)
TS_IP_D49 (n=5)
group_and_day

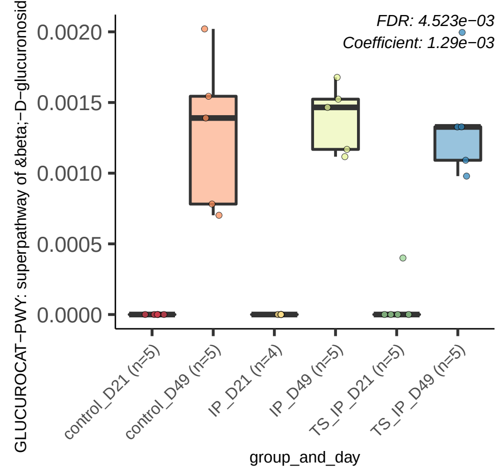
FDR: 4.523e−03
Coefficient: 1.29e−03
GLUCUROCAT−PWY: superpathway of β−D−glucuronosid
0.0020
0.0015
0.0010
0.0005
0.0000
control_D21 (n=5)
control_D49 (n=5)
IP_D21 (n=4)
IP_D49 (n=5)
TS_IP_D21 (n=5)
TS_IP_D49 (n=5)
group_and_day

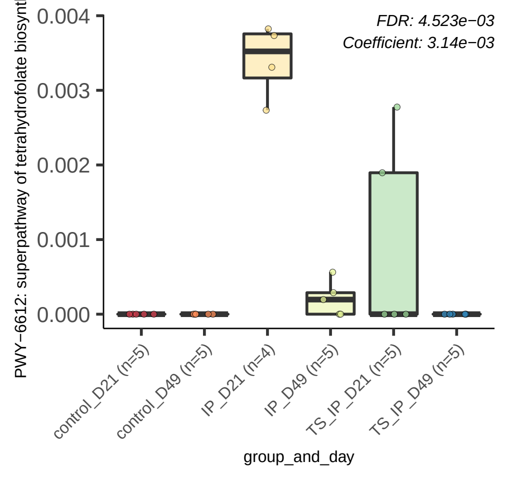
FDR: 4.523e−03
Coefficient: 3.14e−03
PWY−6612: superpathway of tetrahydrofolate biosynt
0.004
0.003
0.002
0.001
0.000
control_D21 (n=5)
control_D49 (n=5)
IP_D21 (n=4)
IP_D49 (n=5)
TS_IP_D21 (n=5)
TS_IP_D49 (n=5)
group_and_day

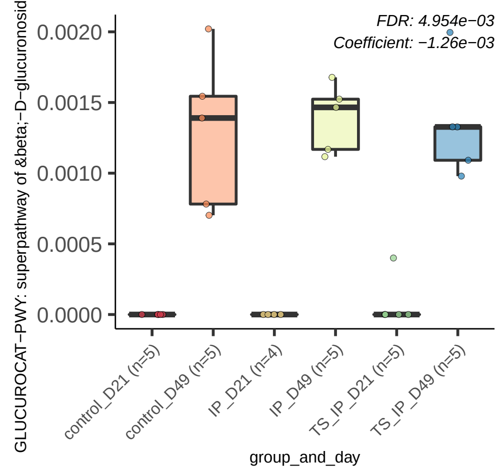

FDR: 4.954e−03
Coefficient: −1.26e−03
GLUCUROCAT−PWY: superpathway of β−D−glucuronosid
0.0020
0.0015
0.0010
0.0005
0.0000
control_D21 (n=5)
control_D49 (n=5)
IP_D21 (n=4)
IP_D49 (n=5)
TS_IP_D21 (n=5)
TS_IP_D49 (n=5)
group_and_day

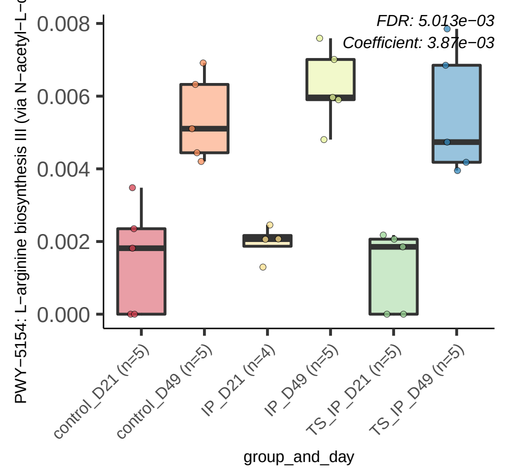

FDR: 5.013e−03
Coefficient: 3.87e−03
PWY−5154: L−arginine biosynthesis III (via N−acetyl−L−c
0.008
0.006
0.004
0.002
0.000
control_D21 (n=5)
control_D49 (n=5)
IP_D21 (n=4)
IP_D49 (n=5)
TS_IP_D21 (n=5)
TS_IP_D49 (n=5)
group_and_day

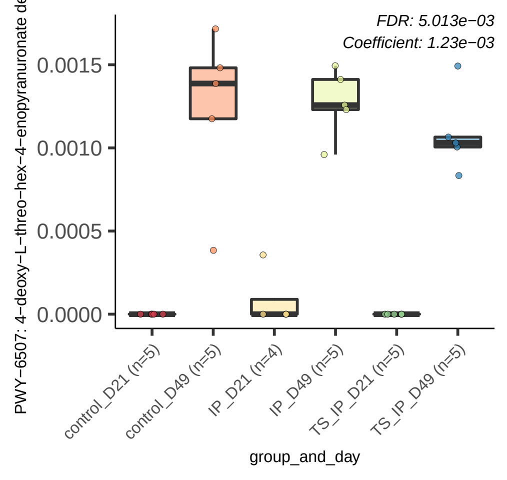
FDR: 5.013e−03
Coefficient: 1.23e−03
PWY−6507: 4−deoxy−L−threo−hex−4−enopyranuronate de
0.0015
0.0010
0.0005
0.0000
control_D21 (n=5)
control_D49 (n=5)
IP_D21 (n=4)
IP_D49 (n=5)
TS_IP_D21 (n=5)
TS_IP_D49 (n=5)
group_and_day

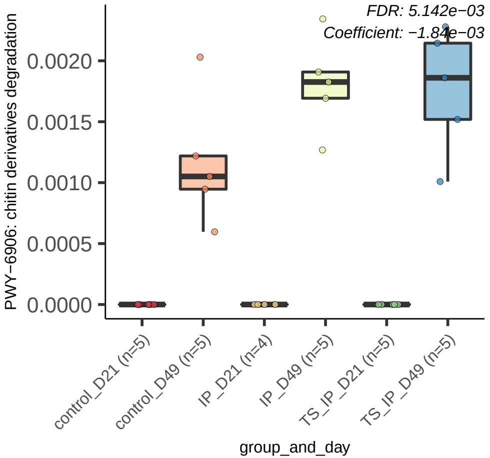

FDR: 5.142e−03
Coefficient: −1.84e−03
PWY−6906: chitin derivatives degradation
0.0020
0.0015
0.0010
0.0005
0.0000
control_D21 (n=5)
control_D49 (n=5)
IP_D21 (n=4)
IP_D49 (n=5)
TS_IP_D21 (n=5)
TS_IP_D49 (n=5)
group_and_day

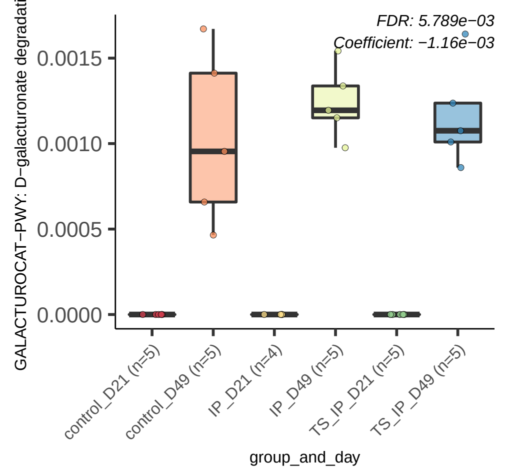
FDR: 5.789e−03
Coefficient: −1.16e−03
GALACTUROCAT−PWY: D−galacturonate degradati
0.0015
0.0010
0.0005
0.0000
control_D21 (n=5)
control_D49 (n=5)
IP_D21 (n=4)
IP_D49 (n=5)
TS_IP_D21 (n=5)
TS_IP_D49 (n=5)
group_and_day

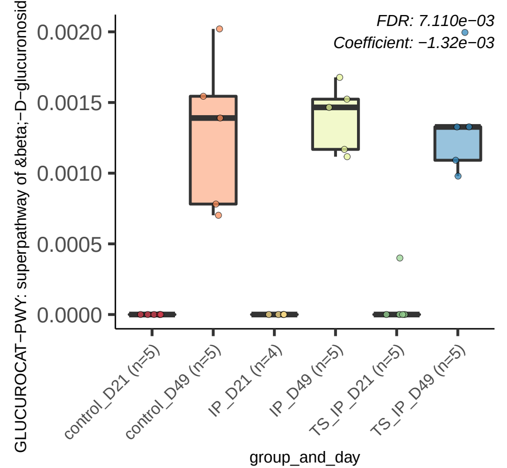
FDR: 7.110e−03
Coefficient: −1.32e−03
GLUCUROCAT−PWY: superpathway of β−D−glucuronosid
0.0020
0.0015
0.0010
0.0005
0.0000
control_D21 (n=5)
control_D49 (n=5)
IP_D21 (n=4)
IP_D49 (n=5)
TS_IP_D21 (n=5)
TS_IP_D49 (n=5)
group_and_day

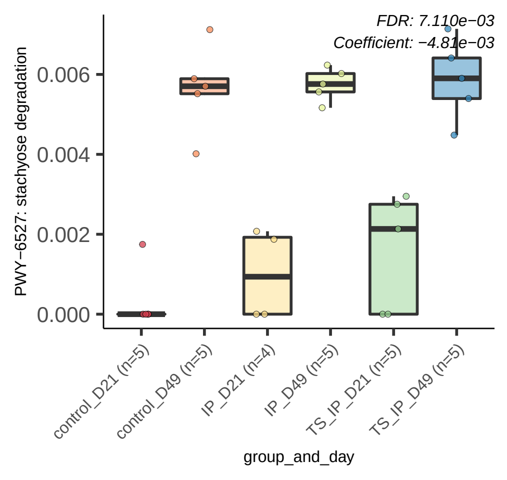

FDR: 7.110e−03
Coefficient: −4.81e−03
PWY−6527: stachyose degradation
0.006
0.004
0.002
0.000
control_D21 (n=5)
control_D49 (n=5)
IP_D21 (n=4)
IP_D49 (n=5)
TS_IP_D21 (n=5)
TS_IP_D49 (n=5)
group_and_day

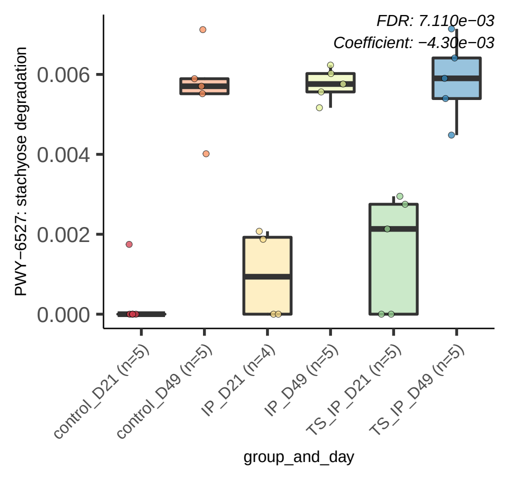
FDR: 7.110e−03
Coefficient: −4.30e−03
PWY−6527: stachyose degradation
0.006
0.004
0.002
0.000
control_D21 (n=5)
control_D49 (n=5)
IP_D21 (n=4)
IP_D49 (n=5)
TS_IP_D21 (n=5)
TS_IP_D49 (n=5)
group_and_day

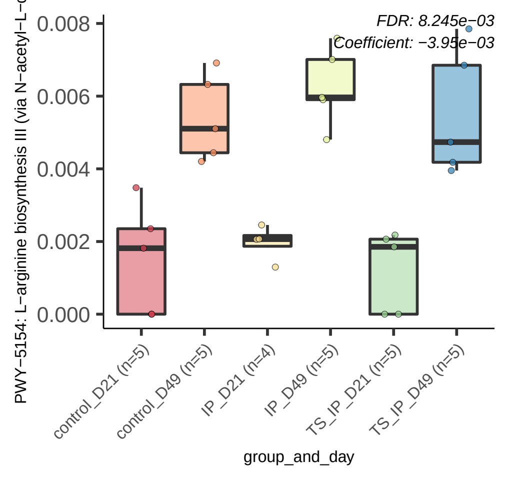
FDR: 8.245e−03
Coefficient: −3.95e−03
PWY−5154: L−arginine biosynthesis III (via N−acetyl−L−c
0.000
0.002
0.004
0.006
0.008
control_D21 (n=5)
control_D49 (n=5)
IP_D21 (n=4)
IP_D49 (n=5)
TS_IP_D21 (n=5)
TS_IP_D49 (n=5)
group_and_day

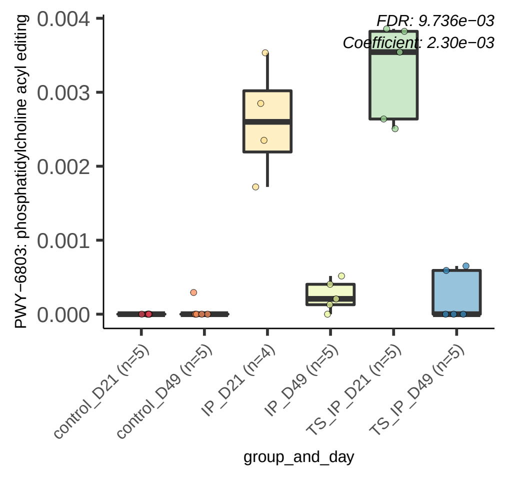

FDR: 9.736e−03
Coefficient: 2.30e−03
PWY−6803: phosphatidylcholine acyl editing
0.004
0.003
0.002
0.001
0.000
control_D21 (n=5)
control_D49 (n=5)
IP_D21 (n=4)
IP_D49 (n=5)
TS_IP_D21 (n=5)
TS_IP_D49 (n=5)
group_and_day

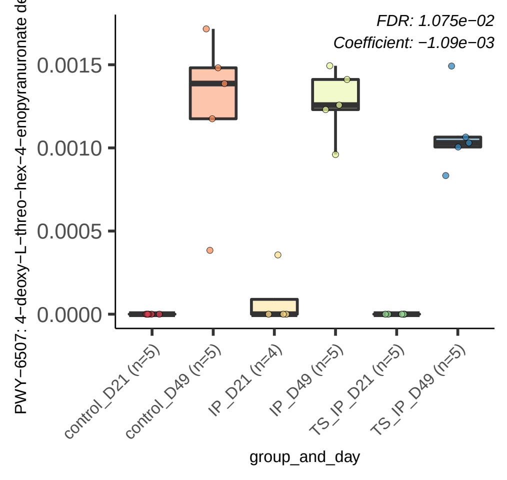
FDR: 1.075e−02
Coefficient: −1.09e−03
PWY−6507: 4−deoxy−L−threo−hex−4−enopyranuronate de
0.0015
0.0010
0.0005
0.0000
control_D21 (n=5)
control_D49 (n=5)
IP_D21 (n=4)
IP_D49 (n=5)
TS_IP_D21 (n=5)
TS_IP_D49 (n=5)
group_and_day

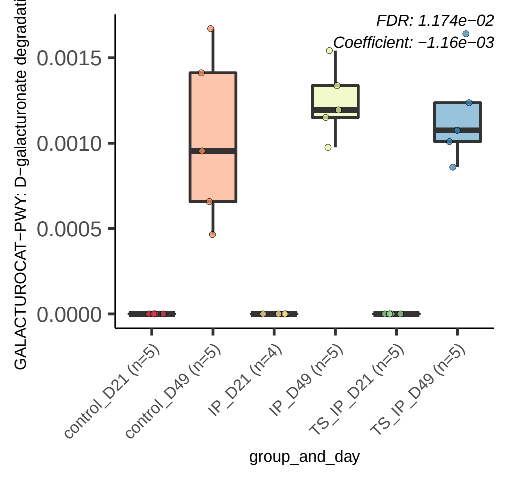
FDR: 1.174e−02
Coefficient: −1.16e−03
GALACTUROCAT−PWY: D−galacturonate degradati
0.0015
0.0010
0.0005
0.0000
control_D21 (n=5)
control_D49 (n=5)
IP_D21 (n=4)
IP_D49 (n=5)
TS_IP_D21 (n=5)
TS_IP_D49 (n=5)
group_and_day

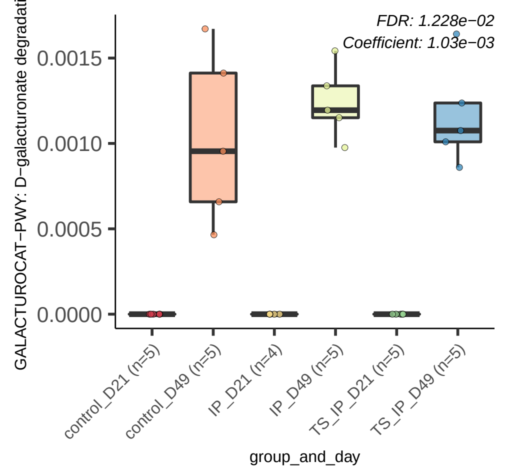
FDR: 1.228e−02
Coefficient: 1.03e−03
GALACTUROCAT−PWY: D−galacturonate degradati
0.0015
0.0010
0.0005
0.0000
control_D21 (n=5)
control_D49 (n=5)
IP_D21 (n=4)
IP_D49 (n=5)
TS_IP_D21 (n=5)
TS_IP_D49 (n=5)
group_and_day

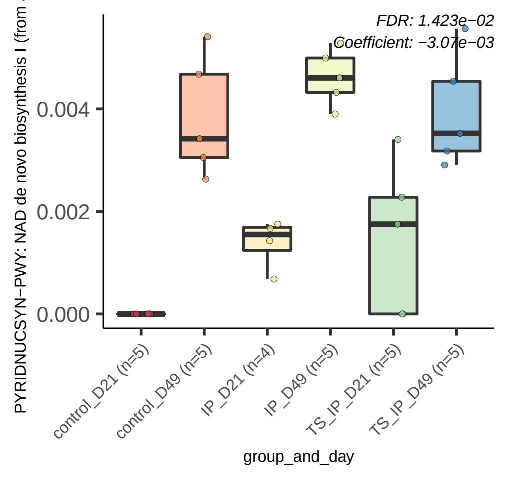
FDR: 1.423e−02
Coefficient: −3.07e−03
PYRIDNUCSYN−PWY: NAD de novo biosynthesis I (from a
0.004
0.002
0.000
control_D21 (n=5)
control_D49 (n=5)
IP_D21 (n=4)
IP_D49 (n=5)
TS_IP_D21 (n=5)
TS_IP_D49 (n=5)
group_and_day

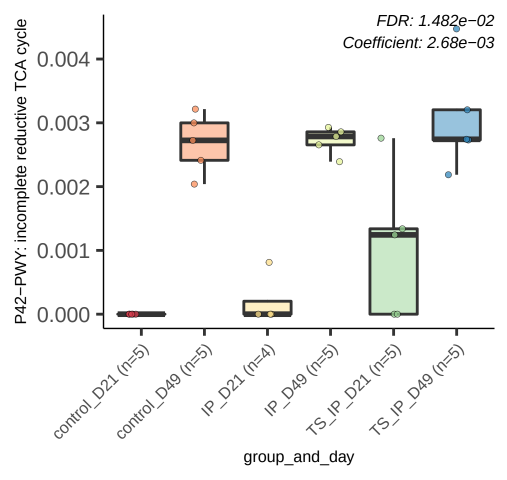

FDR: 1.482e−02
Coefficient: 2.68e−03
P42−PWY: incomplete reductive TCA cycle
0.004
0.003
0.002
0.001
0.000
control_D21 (n=5)
control_D49 (n=5)
IP_D21 (n=4)
IP_D49 (n=5)
TS_IP_D21 (n=5)
TS_IP_D49 (n=5)
group_and_day

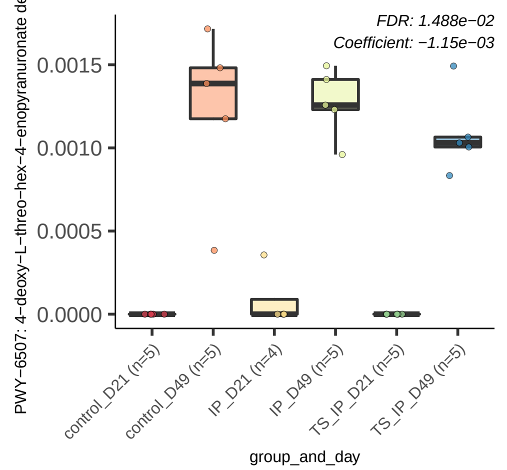

FDR: 1.488e−02
Coefficient: −1.15e−03
PWY−6507: 4−deoxy−L−threo−hex−4−enopyranuronate de
0.0015
0.0010
0.0005
0.0000
control_D21 (n=5)
control_D49 (n=5)
IP_D21 (n=4)
IP_D49 (n=5)
TS_IP_D21 (n=5)
TS_IP_D49 (n=5)
group_and_day

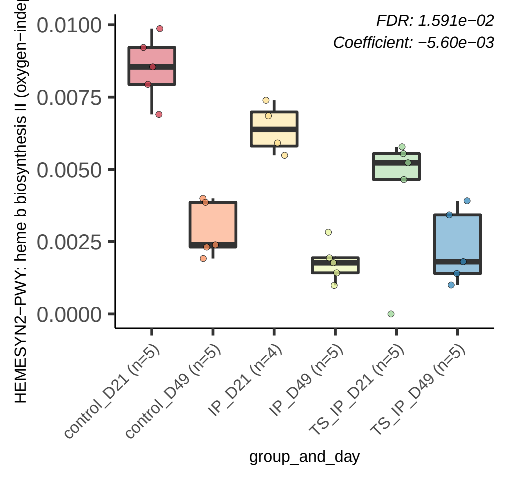
FDR: 1.591e−02
Coefficient: −5.60e−03
HEMESYN2−PWY: heme b biosynthesis II (oxygen−indep
0.0100
0.0075
0.0050
0.0025
0.0000
control_D21 (n=5)
control_D49 (n=5)
IP_D21 (n=4)
IP_D49 (n=5)
TS_IP_D21 (n=5)
TS_IP_D49 (n=5)
group_and_day

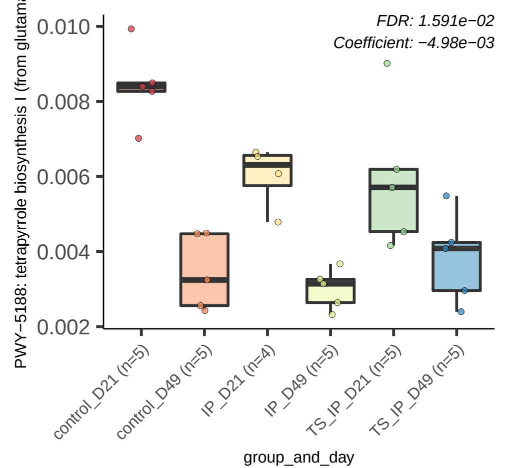
FDR: 1.591e−02
Coefficient: −4.98e−03
PWY−5188: tetrapyrrole biosynthesis I (from glutama
0.010
0.008
0.006
0.004
0.002
control_D21 (n=5)
control_D49 (n=5)
IP_D21 (n=4)
IP_D49 (n=5)
TS_IP_D21 (n=5)
TS_IP_D49 (n=5)
group_and_day

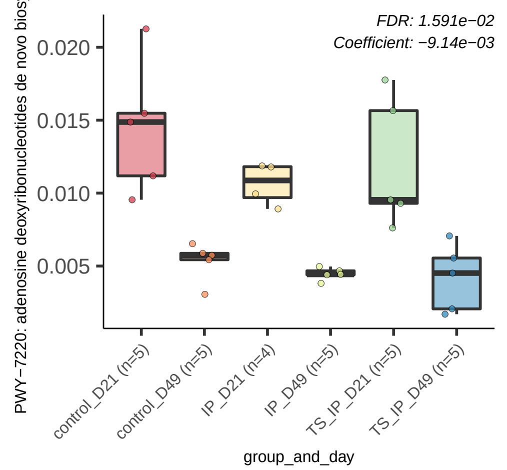

FDR: 1.591e−02
Coefficient: −9.14e−03
PWY−7220: adenosine deoxyribonucleotides de novo bios
0.020
0.015
0.010
0.005
control_D21 (n=5)
control_D49 (n=5)
IP_D21 (n=4)
IP_D49 (n=5)
TS_IP_D21 (n=5)
TS_IP_D49 (n=5)
group_and_day

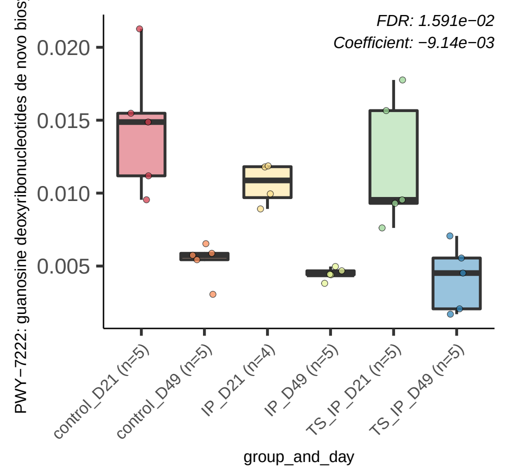
FDR: 1.591e−02
Coefficient: −9.14e−03
PWY−7222: guanosine deoxyribonucleotides de novo bios
0.020
0.015
0.010
0.005
control_D21 (n=5)
control_D49 (n=5)
IP_D21 (n=4)
IP_D49 (n=5)
TS_IP_D21 (n=5)
TS_IP_D49 (n=5)
group_and_day

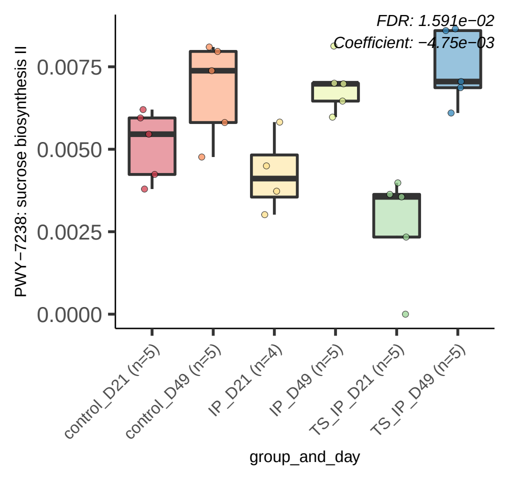

FDR: 1.591e−02
Coefficient: −4.75e−03
PWY−7238: sucrose biosynthesis II
0.0000
0.0025
0.0050
0.0075
control_D21 (n=5)
control_D49 (n=5)
IP_D21 (n=4)
IP_D49 (n=5)
TS_IP_D21 (n=5)
TS_IP_D49 (n=5)
group_and_day

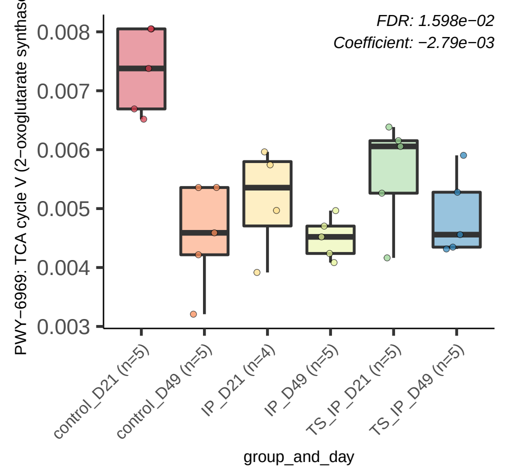

FDR: 1.598e−02
Coefficient: −2.79e−03
PWY−6969: TCA cycle V (2−oxoglutarate synthase
0.008
0.007
0.006
0.005
0.004
0.003
control_D21 (n=5)
control_D49 (n=5)
IP_D21 (n=4)
IP_D49 (n=5)
TS_IP_D21 (n=5)
TS_IP_D49 (n=5)
group_and_day

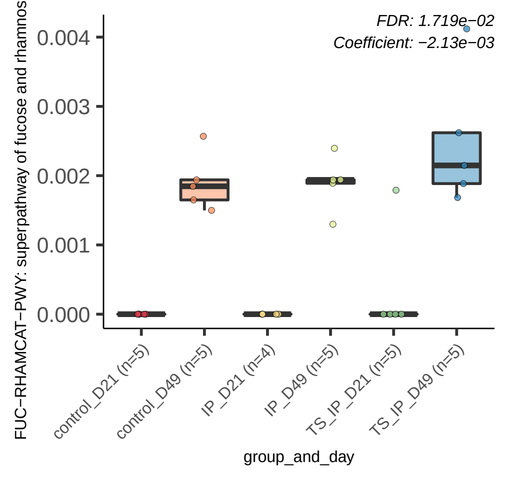
FDR: 1.719e−02
Coefficient: −2.13e−03
FUC−RHAMCAT−PWY: superpathway of fucose and rhamnos
0.004
0.003
0.002
0.001
0.000
control_D21 (n=5)
control_D49 (n=5)
IP_D21 (n=4)
IP_D49 (n=5)
TS_IP_D21 (n=5)
TS_IP_D49 (n=5)
group_and_day

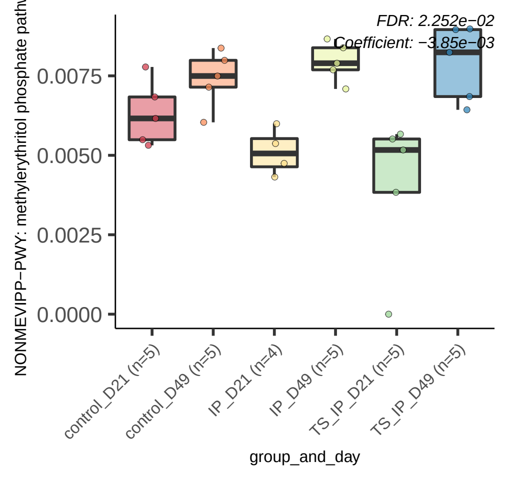

FDR: 2.252e−02
Coefficient: −3.85e−03
NONMEVIPP−PWY: methylerythritol phosphate pathw
0.0075
0.0050
0.0025
0.0000
control_D21 (n=5)
control_D49 (n=5)
IP_D21 (n=4)
IP_D49 (n=5)
TS_IP_D21 (n=5)
TS_IP_D49 (n=5)
group_and_day

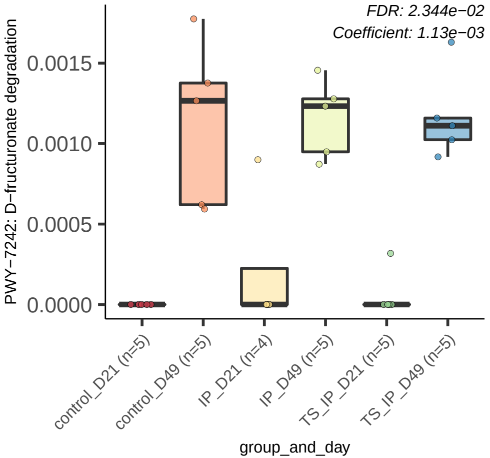

FDR: 2.344e−02
Coefficient: 1.13e−03
PWY−7242: D−fructuronate degradation
0.0015
0.0010
0.0005
0.0000
control_D21 (n=5)
control_D49 (n=5)
IP_D21 (n=4)
IP_D49 (n=5)
TS_IP_D21 (n=5)
TS_IP_D49 (n=5)
group_and_day

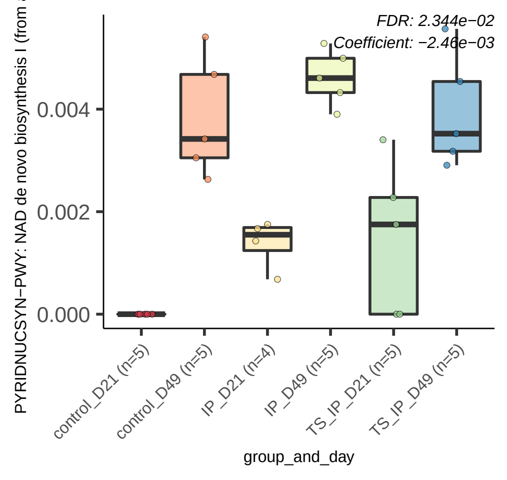
FDR: 2.344e−02
Coefficient: −2.46e−03
PYRIDNUCSYN−PWY: NAD de novo biosynthesis I (from a
0.004
0.002
0.000
control_D21 (n=5)
control_D49 (n=5)
IP_D21 (n=4)
IP_D49 (n=5)
TS_IP_D21 (n=5)
TS_IP_D49 (n=5)
group_and_day

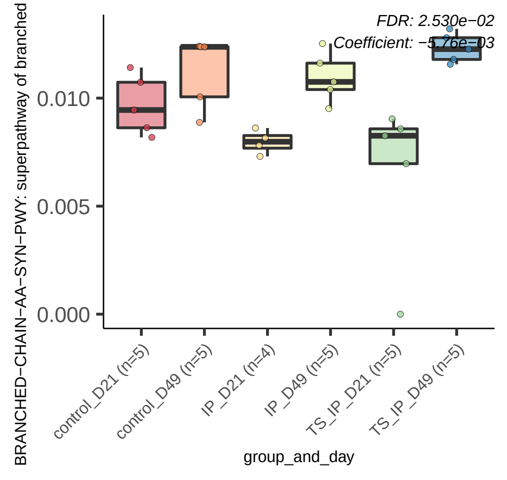
FDR: 2.530e−02
Coefficient: −5.76e−03
BRANCHED−CHAIN−AA−SYN−PWY: superpathway of branched
0.010
0.005
0.000
control_D21 (n=5)
control_D49 (n=5)
IP_D21 (n=4)
IP_D49 (n=5)
TS_IP_D21 (n=5)
TS_IP_D49 (n=5)
group_and_day

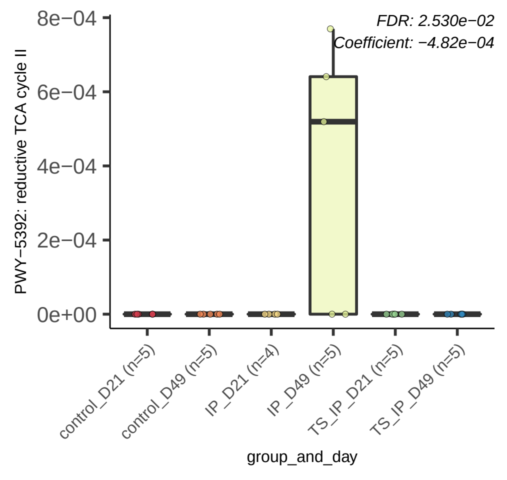

FDR: 2.530e−02
Coefficient: −4.82e−04
PWY−5392: reductive TCA cycle II
8e−04
6e−04
4e−04
2e−04
0e+00
control_D21 (n=5)
control_D49 (n=5)
IP_D21 (n=4)
IP_D49 (n=5)
TS_IP_D21 (n=5)
TS_IP_D49 (n=5)
group_and_day

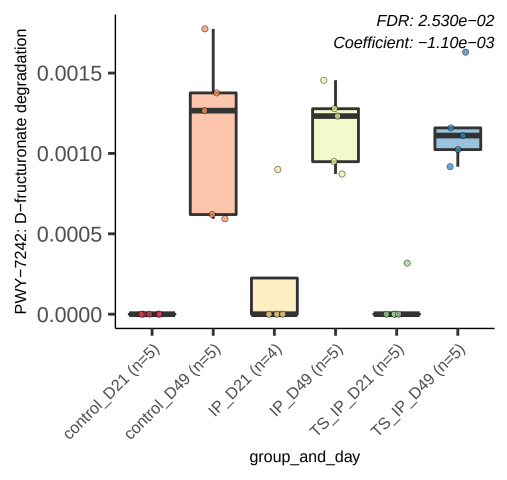

FDR: 2.530e−02
Coefficient: −1.10e−03
PWY−7242: D−fructuronate degradation
0.0015
0.0010
0.0005
0.0000
control_D21 (n=5)
control_D49 (n=5)
IP_D21 (n=4)
IP_D49 (n=5)
TS_IP_D21 (n=5)
TS_IP_D49 (n=5)
group_and_day

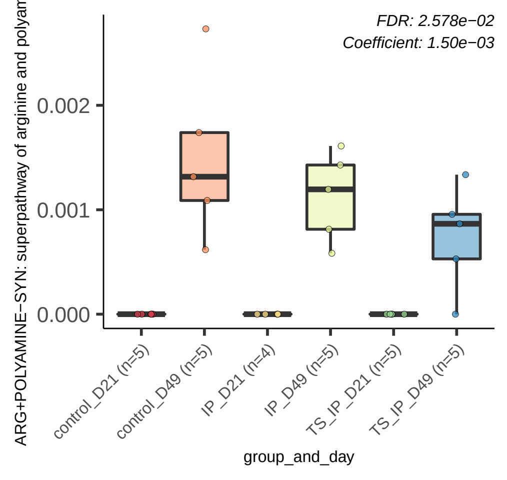

FDR: 2.578e−02
Coefficient: 1.50e−03
ARG+POLYAMINE−SYN: superpathway of arginine and polyam
0.002
0.001
0.000
control_D21 (n=5)
control_D49 (n=5)
IP_D21 (n=4)
IP_D49 (n=5)
TS_IP_D21 (n=5)
TS_IP_D49 (n=5)
group_and_day

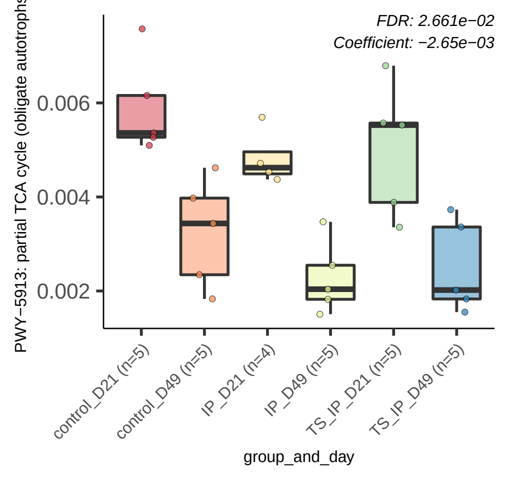
FDR: 2.661e−02
Coefficient: −2.65e−03
PWY−5913: partial TCA cycle (obligate autotrophs
0.006
0.004
0.002
control_D21 (n=5)
control_D49 (n=5)
IP_D21 (n=4)
IP_D49 (n=5)
TS_IP_D21 (n=5)
TS_IP_D49 (n=5)
group_and_day

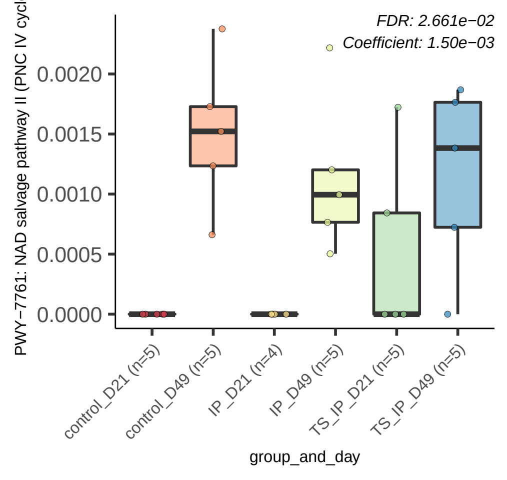

FDR: 2.661e−02
Coefficient: 1.50e−03
PWY−7761: NAD salvage pathway II (PNC IV cycl
0.0020
0.0015
0.0010
0.0005
0.0000
control_D21 (n=5)
control_D49 (n=5)
IP_D21 (n=4)
IP_D49 (n=5)
TS_IP_D21 (n=5)
TS_IP_D49 (n=5)
group_and_day

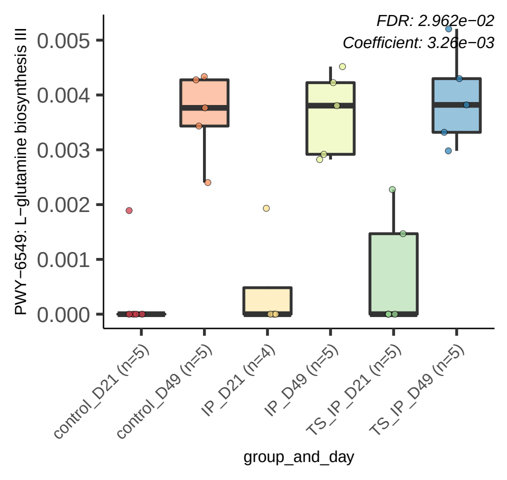

FDR: 2.962e−02
Coefficient: 3.26e−03
PWY−6549: L−glutamine biosynthesis III
0.005
0.004
0.003
0.002
0.001
0.000
control_D21 (n=5)
control_D49 (n=5)
IP_D21 (n=4)
IP_D49 (n=5)
TS_IP_D21 (n=5)
TS_IP_D49 (n=5)
group_and_day

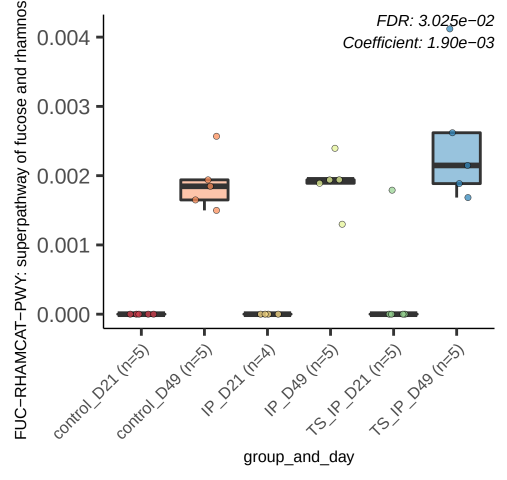

FDR: 3.025e−02
Coefficient: 1.90e−03
FUC−RHAMCAT−PWY: superpathway of fucose and rhamnos
0.004
0.003
0.002
0.001
0.000
control_D21 (n=5)
control_D49 (n=5)
IP_D21 (n=4)
IP_D49 (n=5)
TS_IP_D21 (n=5)
TS_IP_D49 (n=5)
group_and_day

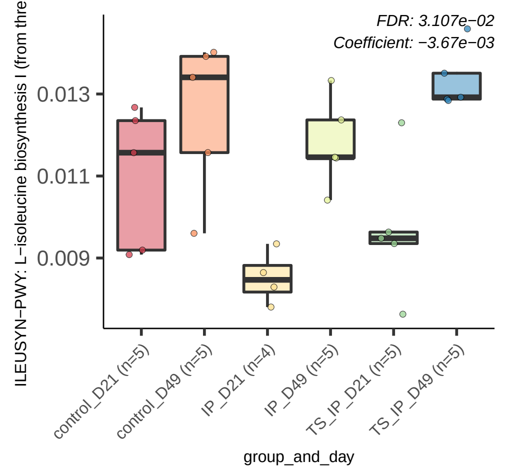
FDR: 3.107e−02
Coefficient: −3.67e−03
ILEUSYN−PWY: L−isoleucine biosynthesis I (from thre
0.013
0.011
0.009
control_D21 (n=5)
control_D49 (n=5)
IP_D21 (n=4)
IP_D49 (n=5)
TS_IP_D21 (n=5)
TS_IP_D49 (n=5)
group_and_day

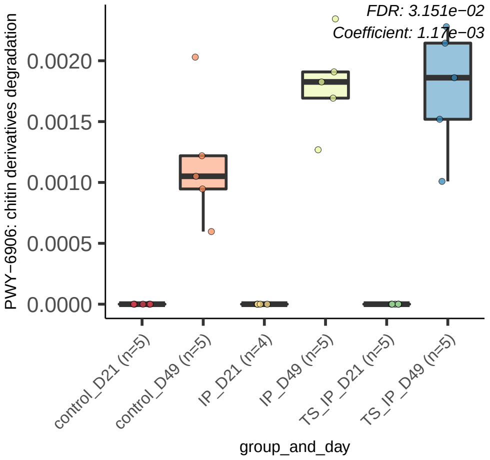

FDR: 3.151e−02
Coefficient: 1.17e−03
PWY−6906: chitin derivatives degradation
0.0020
0.0015
0.0010
0.0005
0.0000
control_D21 (n=5)
control_D49 (n=5)
IP_D21 (n=4)
IP_D49 (n=5)
TS_IP_D21 (n=5)
TS_IP_D49 (n=5)
group_and_day

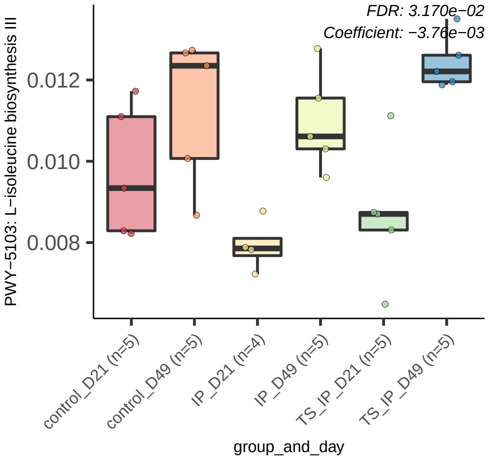
FDR: 3.170e−02
Coefficient: −3.76e−03
PWY−5103: L−isoleucine biosynthesis III
0.012
0.010
0.008
control_D21 (n=5)
control_D49 (n=5)
IP_D21 (n=4)
IP_D49 (n=5)
TS_IP_D21 (n=5)
TS_IP_D49 (n=5)
group_and_day

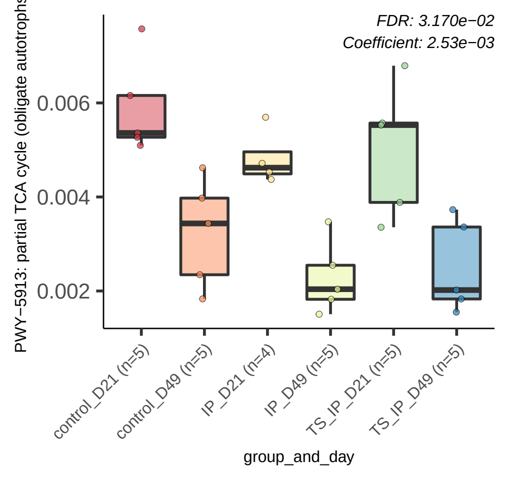

FDR: 3.170e−02
Coefficient: 2.53e−03
PWY−5913: partial TCA cycle (obligate autotrophs
0.006
0.004
0.002
control_D21 (n=5)
control_D49 (n=5)
IP_D21 (n=4)
IP_D49 (n=5)
TS_IP_D21 (n=5)
TS_IP_D49 (n=5)
group_and_day

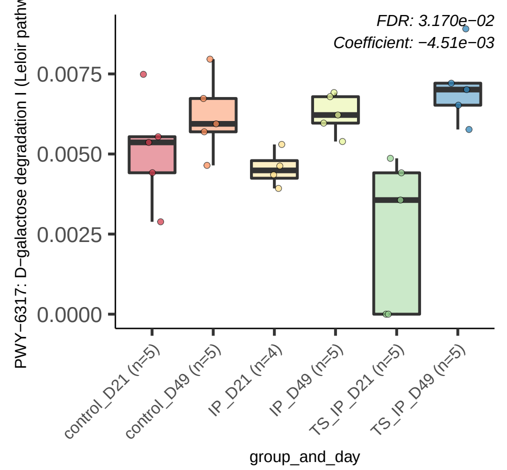
FDR: 3.170e−02
Coefficient: −4.51e−03
PWY−6317: D−galactose degradation I (Leloir pathw
0.0075
0.0050
0.0025
0.0000
control_D21 (n=5)
control_D49 (n=5)
IP_D21 (n=4)
IP_D49 (n=5)
TS_IP_D21 (n=5)
TS_IP_D49 (n=5)
group_and_day

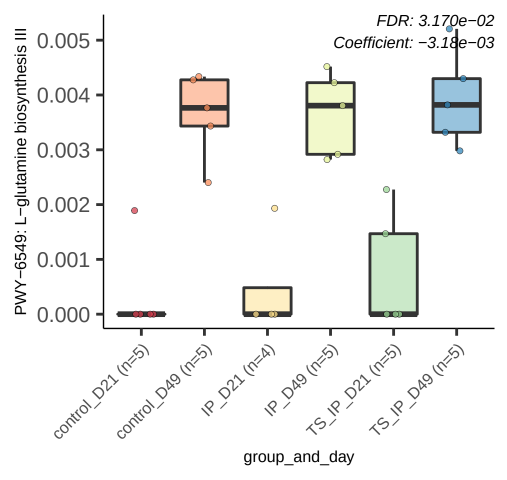

FDR: 3.170e−02
Coefficient: −3.18e−03
PWY−6549: L−glutamine biosynthesis III
0.005
0.004
0.003
0.002
0.001
0.000
control_D21 (n=5)
control_D49 (n=5)
IP_D21 (n=4)
IP_D49 (n=5)
TS_IP_D21 (n=5)
TS_IP_D49 (n=5)
group_and_day

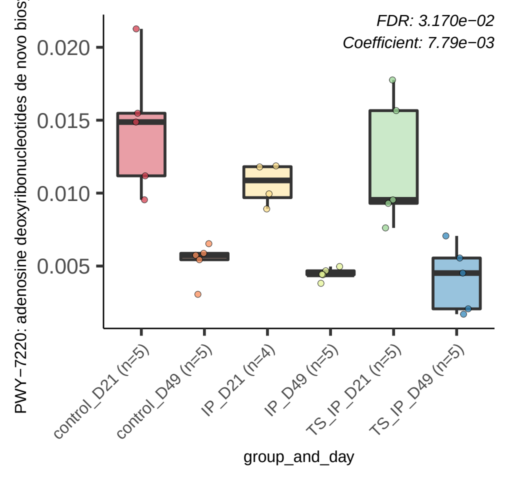
FDR: 3.170e−02
Coefficient: 7.79e−03
PWY−7220: adenosine deoxyribonucleotides de novo bios
0.020
0.015
0.010
0.005
control_D21 (n=5)
control_D49 (n=5)
IP_D21 (n=4)
IP_D49 (n=5)
TS_IP_D21 (n=5)
TS_IP_D49 (n=5)
group_and_day

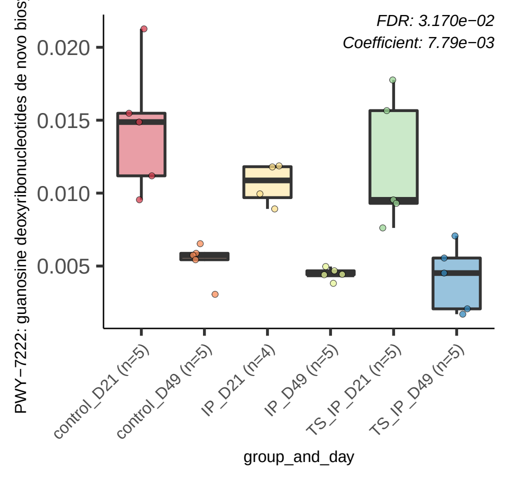

FDR: 3.170e−02
Coefficient: 7.79e−03
PWY−7222: guanosine deoxyribonucleotides de novo bios
0.020
0.015
0.010
0.005
control_D21 (n=5)
control_D49 (n=5)
IP_D21 (n=4)
IP_D49 (n=5)
TS_IP_D21 (n=5)
TS_IP_D49 (n=5)
group_and_day

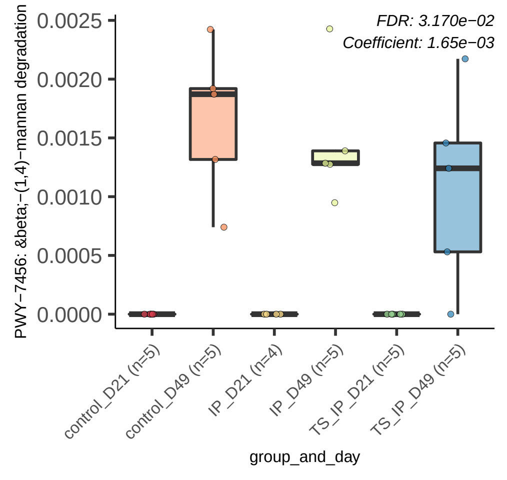
FDR: 3.170e−02
Coefficient: 1.65e−03
PWY−7456: β−(1,4)−mannan degradation
0.0025
0.0020
0.0015
0.0010
0.0005
0.0000
control_D21 (n=5)
control_D49 (n=5)
IP_D21 (n=4)
IP_D49 (n=5)
TS_IP_D21 (n=5)
TS_IP_D49 (n=5)
group_and_day

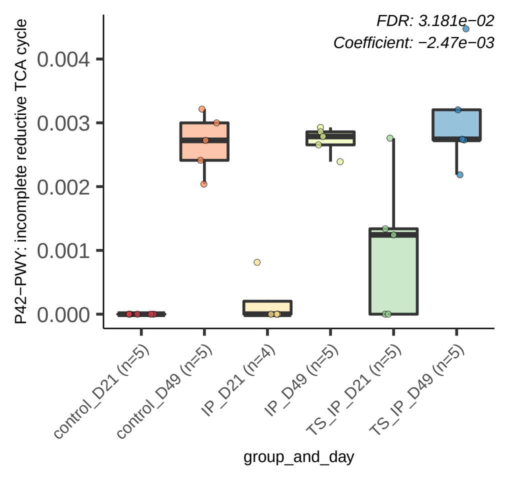

FDR: 3.181e−02
Coefficient: −2.47e−03
P42−PWY: incomplete reductive TCA cycle
0.004
0.003
0.002
0.001
0.000
control_D21 (n=5)
control_D49 (n=5)
IP_D21 (n=4)
IP_D49 (n=5)
TS_IP_D21 (n=5)
TS_IP_D49 (n=5)
group_and_day

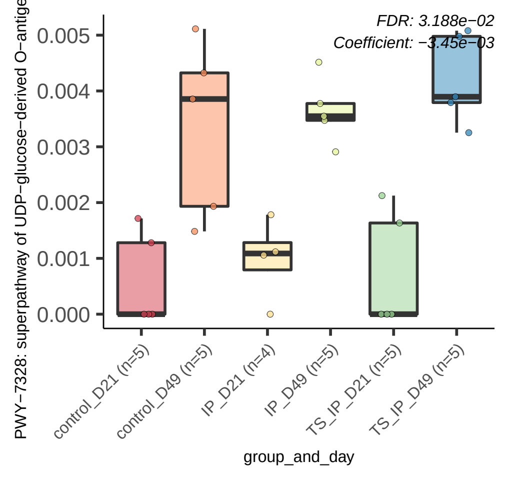
FDR: 3.188e−02
Coefficient: −3.45e−03
PWY−7328: superpathway of UDP−glucose−derived O−antige
0.005
0.004
0.003
0.002
0.001
0.000
control_D21 (n=5)
control_D49 (n=5)
IP_D21 (n=4)
IP_D49 (n=5)
TS_IP_D21 (n=5)
TS_IP_D49 (n=5)
group_and_day

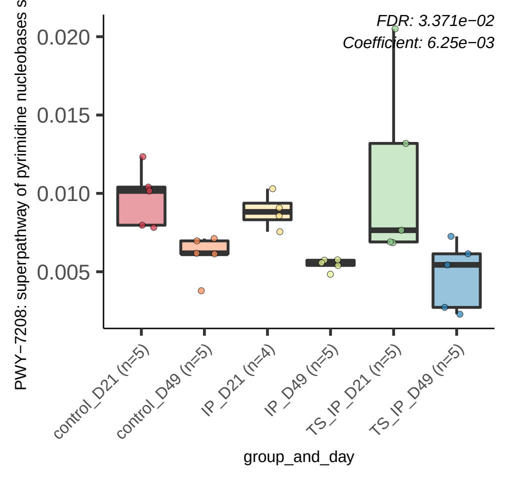
FDR: 3.371e−02
Coefficient: 6.25e−03
PWY−7208: superpathway of pyrimidine nucleobases s
0.020
0.015
0.010
0.005
control_D21 (n=5)
control_D49 (n=5)
IP_D21 (n=4)
IP_D49 (n=5)
TS_IP_D21 (n=5)
TS_IP_D49 (n=5)
group_and_day

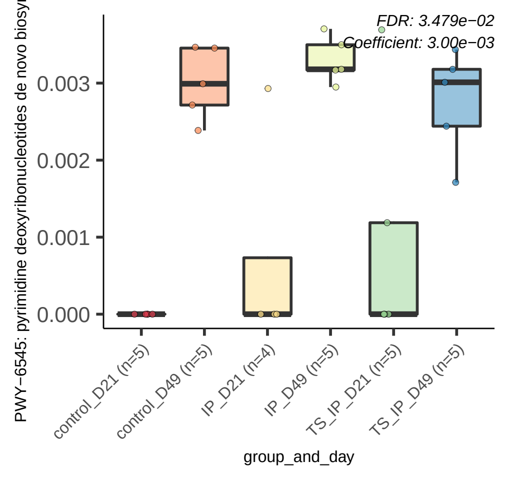

FDR: 3.479e−02
Coefficient: 3.00e−03
PWY−6545: pyrimidine deoxyribonucleotides de novo biosy
0.003
0.002
0.001
0.000
control_D21 (n=5)
control_D49 (n=5)
IP_D21 (n=4)
IP_D49 (n=5)
TS_IP_D21 (n=5)
TS_IP_D49 (n=5)
group_and_day

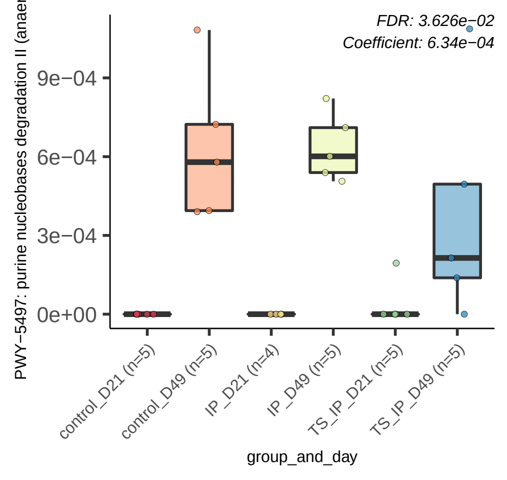

FDR: 3.626e−02
Coefficient: 6.34e−04
PWY−5497: purine nucleobases degradation II (anaer
9e−04
6e−04
3e−04
0e+00
control_D21 (n=5)
control_D49 (n=5)
IP_D21 (n=4)
IP_D49 (n=5)
TS_IP_D21 (n=5)
TS_IP_D49 (n=5)
group_and_day

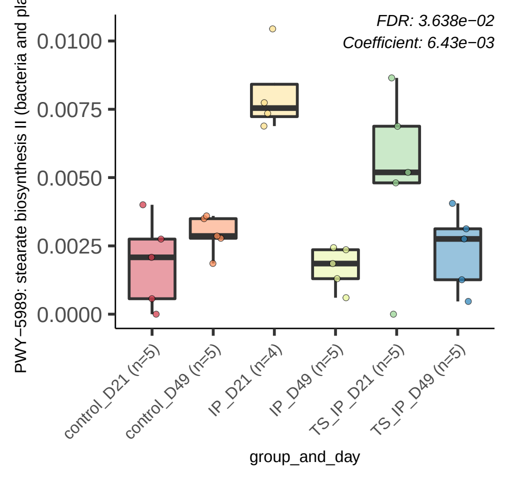
FDR: 3.638e−02
Coefficient: 6.43e−03
PWY−5989: stearate biosynthesis II (bacteria and pla
0.0100
0.0075
0.0050
0.0025
0.0000
control_D21 (n=5)
control_D49 (n=5)
IP_D21 (n=4)
IP_D49 (n=5)
TS_IP_D21 (n=5)
TS_IP_D49 (n=5)
group_and_day

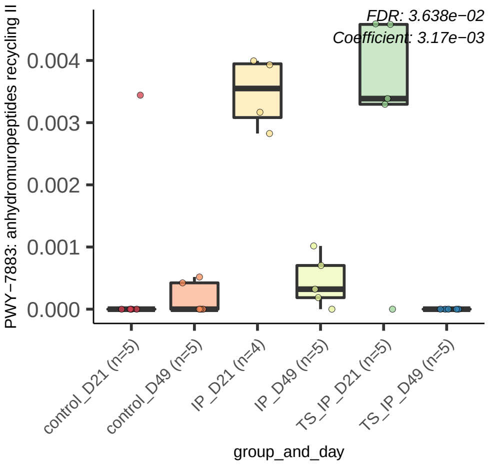

FDR: 3.638e−02
Coefficient: 3.17e−03
PWY−7883: anhydromuropeptides recycling II
0.000
0.001
0.002
0.003
0.004
control_D21 (n=5)
control_D49 (n=5)
IP_D21 (n=4)
IP_D49 (n=5)
TS_IP_D21 (n=5)
TS_IP_D49 (n=5)
group_and_day

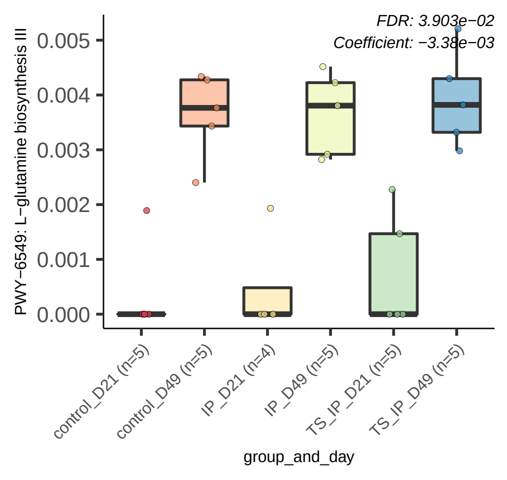
FDR: 3.903e−02
Coefficient: −3.38e−03
PWY−6549: L−glutamine biosynthesis III
0.005
0.004
0.003
0.002
0.001
0.000
control_D21 (n=5)
control_D49 (n=5)
IP_D21 (n=4)
IP_D49 (n=5)
TS_IP_D21 (n=5)
TS_IP_D49 (n=5)
group_and_day

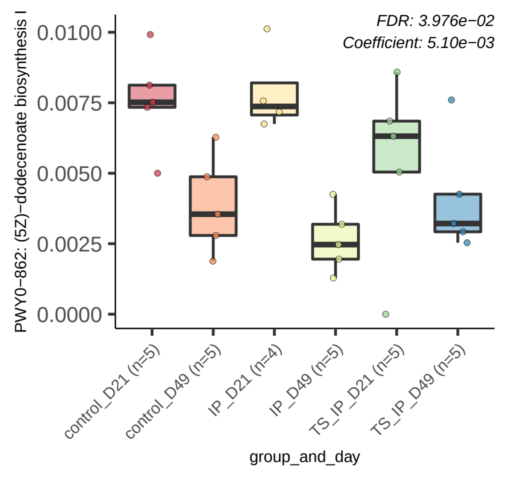

FDR: 3.976e−02
Coefficient: 5.10e−03
PWY0−862: (5Z)−dodecenoate biosynthesis I
0.0100
0.0075
0.0050
0.0025
0.0000
control_D21 (n=5)
control_D49 (n=5)
IP_D21 (n=4)
IP_D49 (n=5)
TS_IP_D21 (n=5)
TS_IP_D49 (n=5)
group_and_day

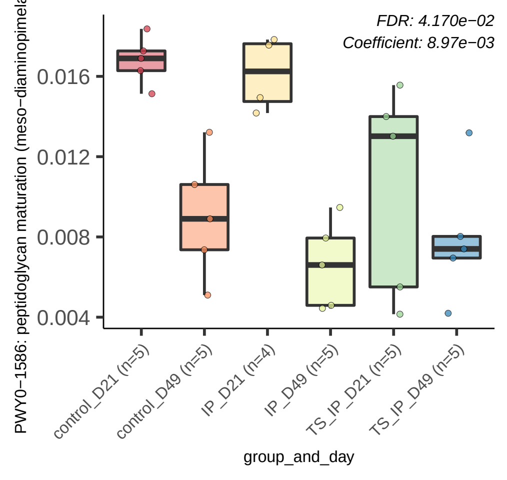

FDR: 4.170e−02
Coefficient: 8.97e−03
PWY0−1586: peptidoglycan maturation (meso−diaminopimela
0.016
0.012
0.008
0.004
control_D21 (n=5)
control_D49 (n=5)
IP_D21 (n=4)
IP_D49 (n=5)
TS_IP_D21 (n=5)
TS_IP_D49 (n=5)
group_and_day

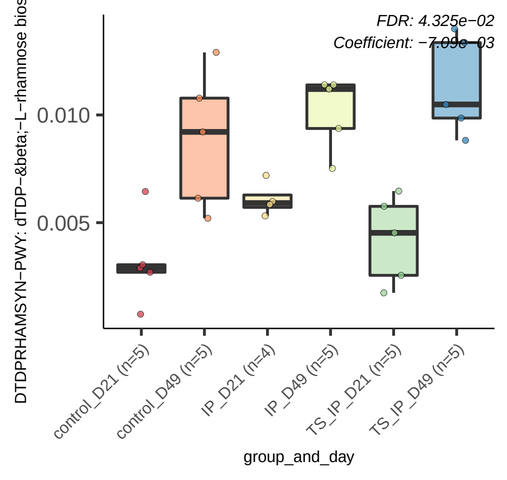

FDR: 4.325e−02
Coefficient: −7.09e−03
DTDPRHAMSYN−PWY: dTDP−β−L−rhamnose bios
0.010
0.005
control_D21 (n=5)
control_D49 (n=5)
IP_D21 (n=4)
IP_D49 (n=5)
TS_IP_D21 (n=5)
TS_IP_D49 (n=5)
group_and_day

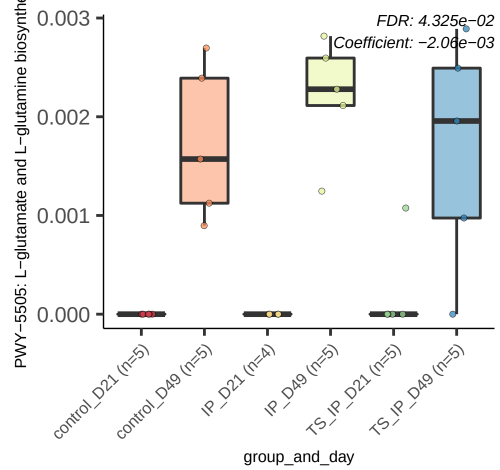

FDR: 4.325e−02
Coefficient: −2.06e−03
PWY−5505: L−glutamate and L−glutamine biosynthe
0.003
0.002
0.001
0.000
control_D21 (n=5)
control_D49 (n=5)
IP_D21 (n=4)
IP_D49 (n=5)
TS_IP_D21 (n=5)
TS_IP_D49 (n=5)
group_and_day

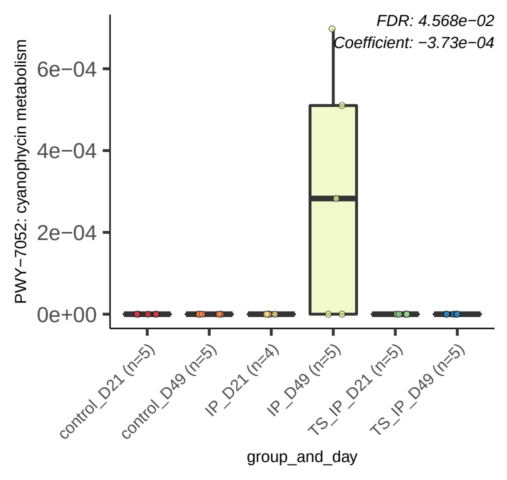
FDR: 4.568e−02
Coefficient: −3.73e−04
PWY−7052: cyanophycin metabolism
6e−04
4e−04
2e−04
0e+00
control_D21 (n=5)
control_D49 (n=5)
IP_D21 (n=4)
IP_D49 (n=5)
TS_IP_D21 (n=5)
TS_IP_D49 (n=5)
group_and_day

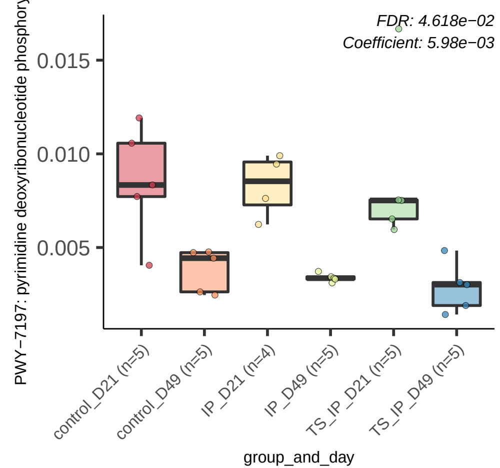
FDR: 4.618e−02
Coefficient: 5.98e−03
PWY−7197: pyrimidine deoxyribonucleotide phosphory
0.015
0.010
0.005
control_D21 (n=5)
control_D49 (n=5)
IP_D21 (n=4)
IP_D49 (n=5)
TS_IP_D21 (n=5)
TS_IP_D49 (n=5)
group_and_day

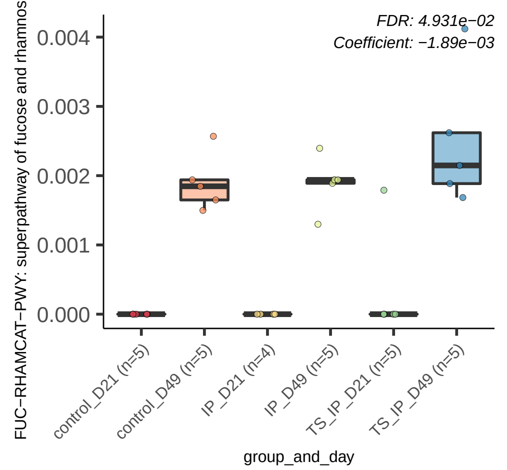
FDR: 4.931e−02
Coefficient: −1.89e−03
FUC−RHAMCAT−PWY: superpathway of fucose and rhamnos
0.004
0.003
0.002
0.001
0.000
control_D21 (n=5)
control_D49 (n=5)
IP_D21 (n=4)
IP_D49 (n=5)
TS_IP_D21 (n=5)
TS_IP_D49 (n=5)
group_and_day

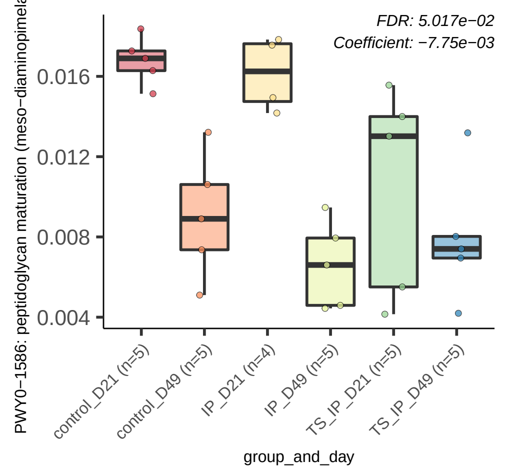

FDR: 5.017e−02
Coefficient: −7.75e−03
PWY0−1586: peptidoglycan maturation (meso−diaminopimela
0.016
0.012
0.008
0.004
control_D21 (n=5)
control_D49 (n=5)
IP_D21 (n=4)
IP_D49 (n=5)
TS_IP_D21 (n=5)
TS_IP_D49 (n=5)
group_and_day

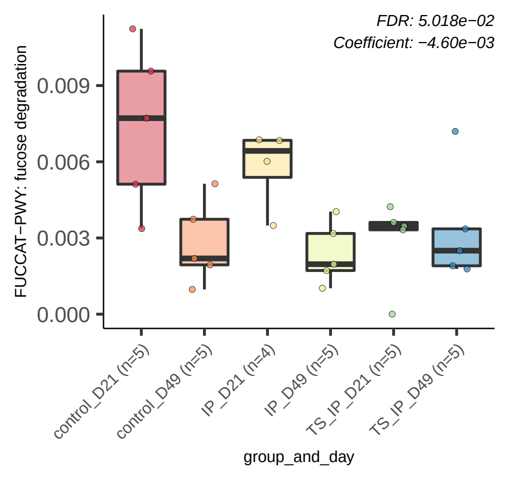

FDR: 5.018e−02
Coefficient: −4.60e−03
FUCCAT−PWY: fucose degradation
0.009
0.006
0.003
0.000
control_D21 (n=5)
control_D49 (n=5)
IP_D21 (n=4)
IP_D49 (n=5)
TS_IP_D21 (n=5)
TS_IP_D49 (n=5)
group_and_day

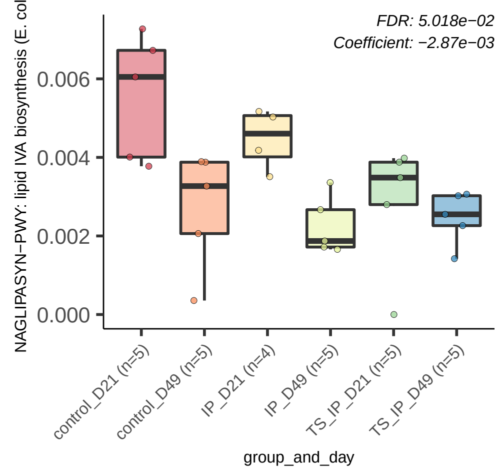

FDR: 5.018e−02
Coefficient: −2.87e−03
NAGLIPASYN−PWY: lipid IVA biosynthesis (E. col
0.000
0.002
0.004
0.006
control_D21 (n=5)
control_D49 (n=5)
IP_D21 (n=4)
IP_D49 (n=5)
TS_IP_D21 (n=5)
TS_IP_D49 (n=5)
group_and_day

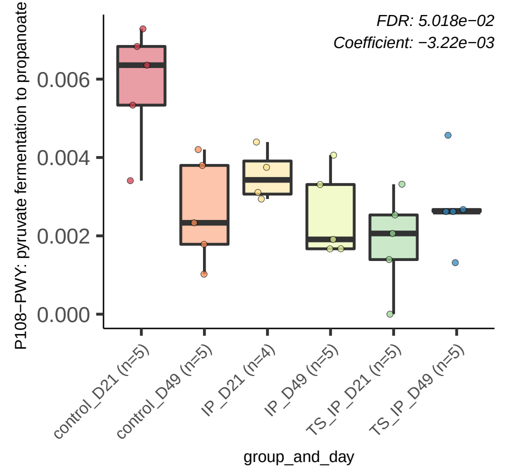
FDR: 5.018e−02
Coefficient: −3.22e−03
P108−PWY: pyruvate fermentation to propanoate
0.006
0.004
0.002
0.000
control_D21 (n=5)
control_D49 (n=5)
IP_D21 (n=4)
IP_D49 (n=5)
TS_IP_D21 (n=5)
TS_IP_D49 (n=5)
group_and_day

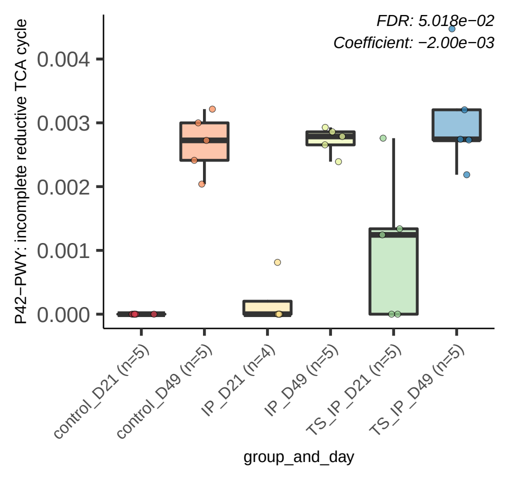

FDR: 5.018e−02
Coefficient: −2.00e−03
P42−PWY: incomplete reductive TCA cycle
0.004
0.003
0.002
0.001
0.000
control_D21 (n=5)
control_D49 (n=5)
IP_D21 (n=4)
IP_D49 (n=5)
TS_IP_D21 (n=5)
TS_IP_D49 (n=5)
group_and_day

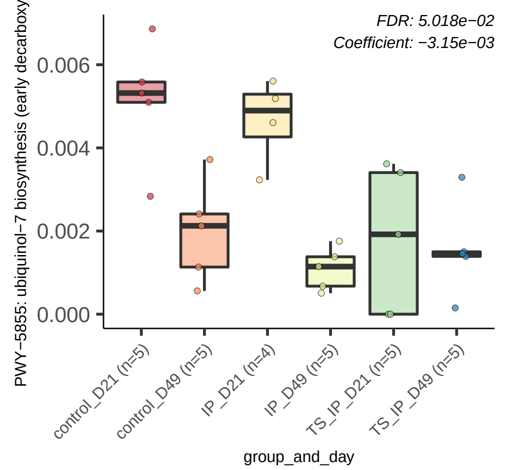

FDR: 5.018e−02
Coefficient: −3.15e−03
PWY−5855: ubiquinol−7 biosynthesis (early decarboxy
0.000
0.002
0.004
0.006
control_D21 (n=5)
control_D49 (n=5)
IP_D21 (n=4)
IP_D49 (n=5)
TS_IP_D21 (n=5)
TS_IP_D49 (n=5)
group_and_day

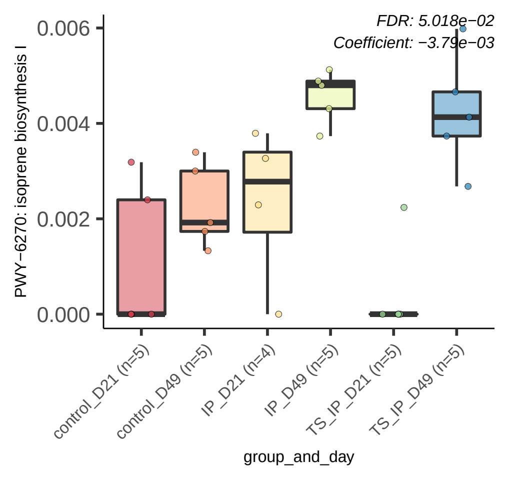
FDR: 5.018e−02
Coefficient: −3.79e−03
PWY−6270: isoprene biosynthesis I
0.006
0.004
0.002
0.000
control_D21 (n=5)
control_D49 (n=5)
IP_D21 (n=4)
IP_D49 (n=5)
TS_IP_D21 (n=5)
TS_IP_D49 (n=5)
group_and_day

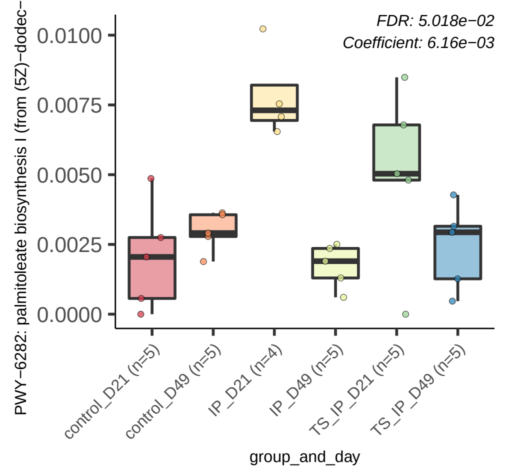

FDR: 5.018e−02
Coefficient: 6.16e−03
PWY−6282: palmitoleate biosynthesis I (from (5Z)−dodec−
0.0100
0.0075
0.0050
0.0025
0.0000
control_D21 (n=5)
control_D49 (n=5)
IP_D21 (n=4)
IP_D49 (n=5)
TS_IP_D21 (n=5)
TS_IP_D49 (n=5)
group_and_day

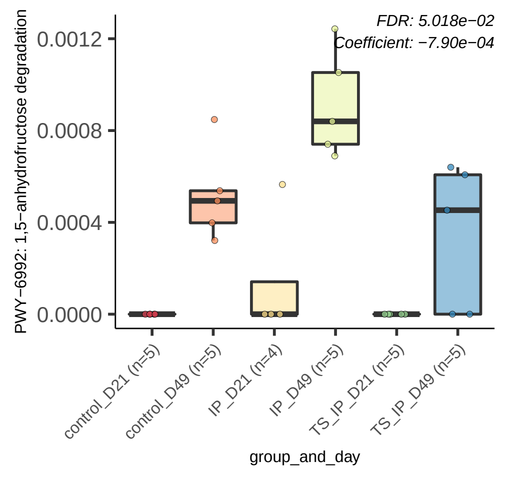

FDR: 5.018e−02
Coefficient: −7.90e−04
PWY−6992: 1,5−anhydrofructose degradation
0.0012
0.0008
0.0004
0.0000
control_D21 (n=5)
control_D49 (n=5)
IP_D21 (n=4)
IP_D49 (n=5)
TS_IP_D21 (n=5)
TS_IP_D49 (n=5)
group_and_day

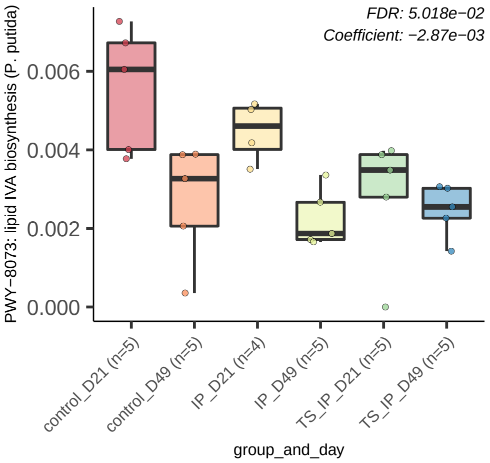
FDR: 5.018e−02
Coefficient: −2.87e−03
PWY−8073: lipid IVA biosynthesis (P. putida)
0.006
0.004
0.002
0.000
control_D21 (n=5)
control_D49 (n=5)
IP_D21 (n=4)
IP_D49 (n=5)
TS_IP_D21 (n=5)
TS_IP_D49 (n=5)
group_and_day

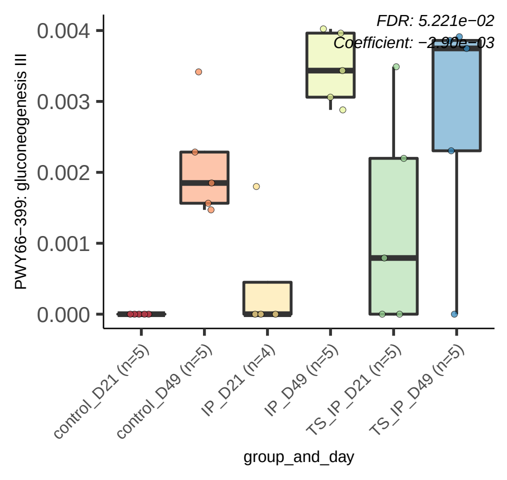
FDR: 5.221e−02
Coefficient: −2.90e−03
PWY66−399: gluconeogenesis III
0.004
0.003
0.002
0.001
0.000
control_D21 (n=5)
control_D49 (n=5)
IP_D21 (n=4)
IP_D49 (n=5)
TS_IP_D21 (n=5)
TS_IP_D49 (n=5)
group_and_day

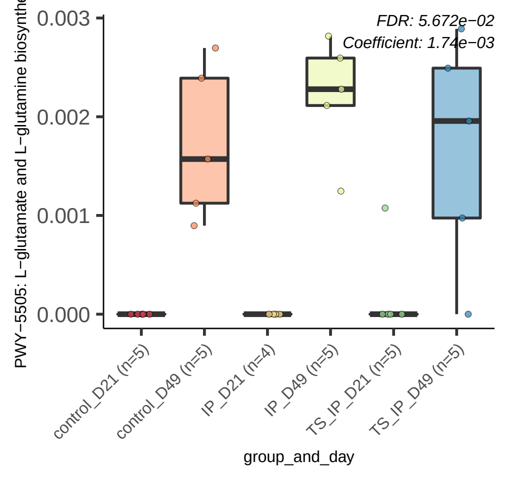
FDR: 5.672e−02
Coefficient: 1.74e−03
PWY−5505: L−glutamate and L−glutamine biosynthe
0.003
0.002
0.001
0.000
control_D21 (n=5)
control_D49 (n=5)
IP_D21 (n=4)
IP_D49 (n=5)
TS_IP_D21 (n=5)
TS_IP_D49 (n=5)
group_and_day

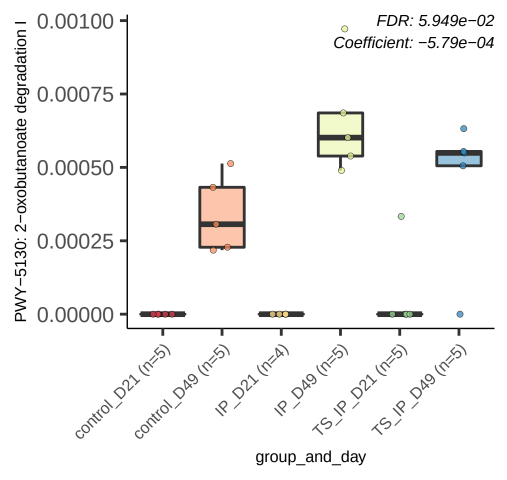

FDR: 5.949e−02
Coefficient: −5.79e−04
PWY−5130: 2−oxobutanoate degradation I
0.00100
0.00075
0.00050
0.00025
0.00000
control_D21 (n=5)
control_D49 (n=5)
IP_D21 (n=4)
IP_D49 (n=5)
TS_IP_D21 (n=5)
TS_IP_D49 (n=5)
group_and_day

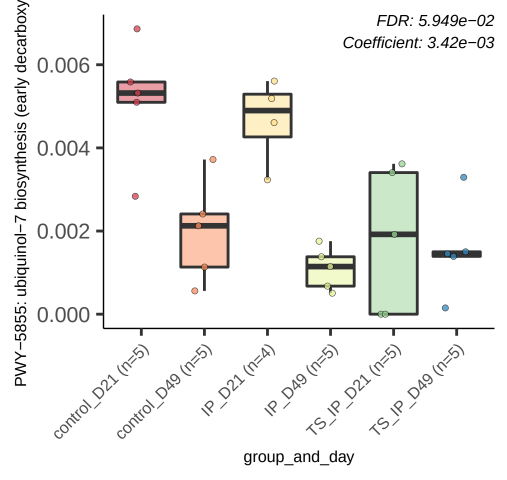
FDR: 5.949e−02
Coefficient: 3.42e−03
PWY−5855: ubiquinol−7 biosynthesis (early decarboxy
0.006
0.004
0.002
0.000
control_D21 (n=5)
control_D49 (n=5)
IP_D21 (n=4)
IP_D49 (n=5)
TS_IP_D21 (n=5)
TS_IP_D49 (n=5)
group_and_day

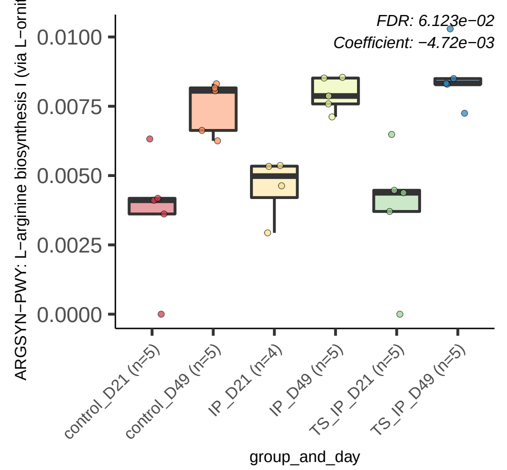
FDR: 6.123e−02
Coefficient: −4.72e−03
ARGSYN−PWY: L−arginine biosynthesis I (via L−orni
0.0100
0.0075
0.0050
0.0025
0.0000
control_D21 (n=5)
control_D49 (n=5)
IP_D21 (n=4)
IP_D49 (n=5)
TS_IP_D21 (n=5)
TS_IP_D49 (n=5)
group_and_day

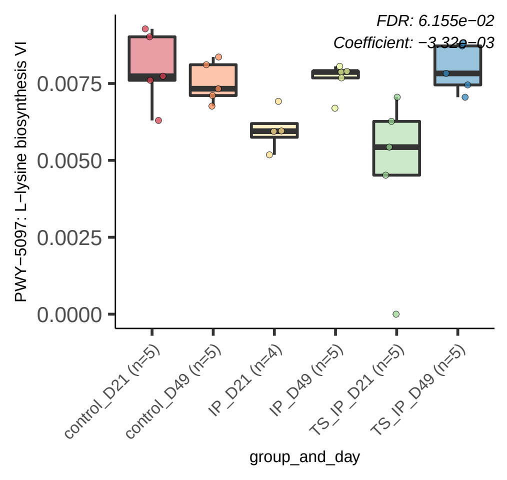
FDR: 6.155e−02
Coefficient: −3.32e−03
PWY−5097: L−lysine biosynthesis VI
0.0075
0.0050
0.0025
0.0000
control_D21 (n=5)
control_D49 (n=5)
IP_D21 (n=4)
IP_D49 (n=5)
TS_IP_D21 (n=5)
TS_IP_D49 (n=5)
group_and_day

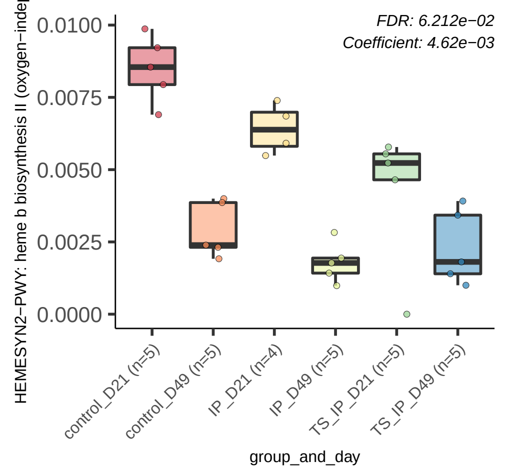

FDR: 6.212e−02
Coefficient: 4.62e−03
HEMESYN2−PWY: heme b biosynthesis II (oxygen−indep
0.0100
0.0075
0.0050
0.0025
0.0000
control_D21 (n=5)
control_D49 (n=5)
IP_D21 (n=4)
IP_D49 (n=5)
TS_IP_D21 (n=5)
TS_IP_D49 (n=5)
group_and_day

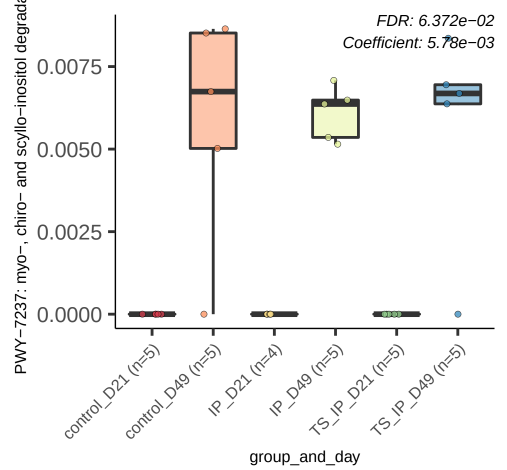
FDR: 6.372e−02
Coefficient: 5.78e−03
PWY−7237: myo−, chiro− and scyllo−inositol degrada
0.0075
0.0050
0.0025
0.0000
control_D21 (n=5)
control_D49 (n=5)
IP_D21 (n=4)
IP_D49 (n=5)
TS_IP_D21 (n=5)
TS_IP_D49 (n=5)
group_and_day

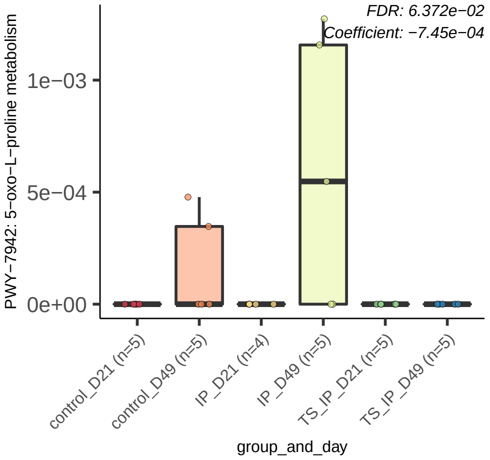

FDR: 6.372e−02
Coefficient: −7.45e−04
PWY−7942: 5−oxo−L−proline metabolism
1e−03
5e−04
0e+00
control_D21 (n=5)
control_D49 (n=5)
IP_D21 (n=4)
IP_D49 (n=5)
TS_IP_D21 (n=5)
TS_IP_D49 (n=5)
group_and_day

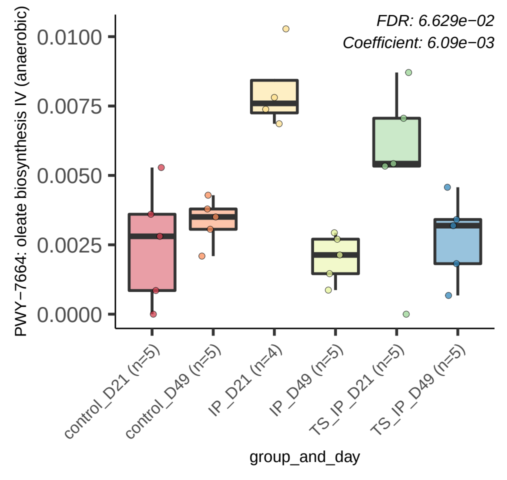

FDR: 6.629e−02
Coefficient: 6.09e−03
PWY−7664: oleate biosynthesis IV (anaerobic)
0.0100
0.0075
0.0050
0.0025
0.0000
control_D21 (n=5)
control_D49 (n=5)
IP_D21 (n=4)
IP_D49 (n=5)
TS_IP_D21 (n=5)
TS_IP_D49 (n=5)
group_and_day

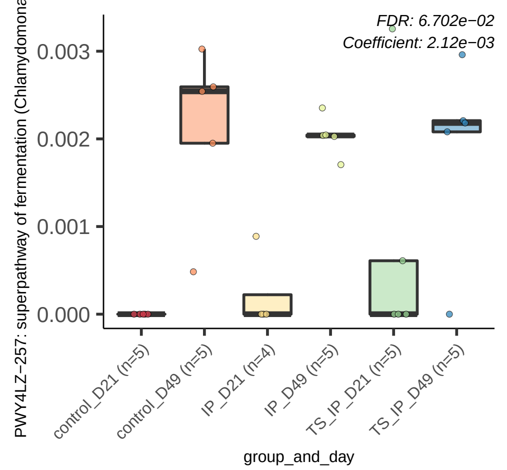

FDR: 6.702e−02
Coefficient: 2.12e−03
PWY4LZ−257: superpathway of fermentation (Chlamydomona
0.003
0.002
0.001
0.000
control_D21 (n=5)
control_D49 (n=5)
IP_D21 (n=4)
IP_D49 (n=5)
TS_IP_D21 (n=5)
TS_IP_D49 (n=5)
group_and_day

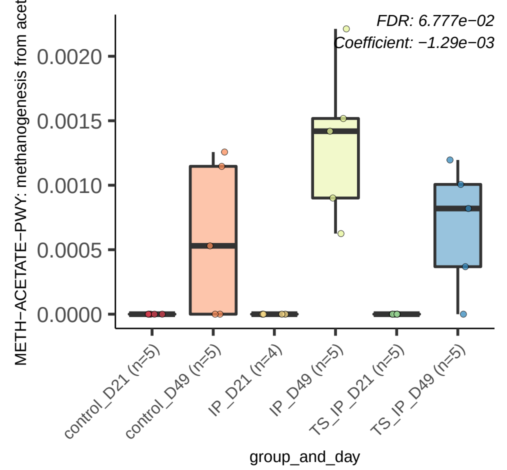

FDR: 6.777e−02
Coefficient: −1.29e−03
METH−ACETATE−PWY: methanogenesis from acet
0.0020
0.0015
0.0010
0.0005
0.0000
control_D21 (n=5)
control_D49 (n=5)
IP_D21 (n=4)
IP_D49 (n=5)
TS_IP_D21 (n=5)
TS_IP_D49 (n=5)
group_and_day

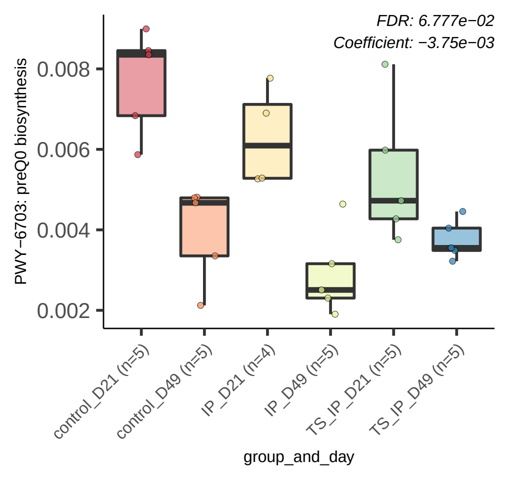
FDR: 6.777e−02
Coefficient: −3.75e−03
PWY−6703: preQ0 biosynthesis
0.008
0.006
0.004
0.002
control_D21 (n=5)
control_D49 (n=5)
IP_D21 (n=4)
IP_D49 (n=5)
TS_IP_D21 (n=5)
TS_IP_D49 (n=5)
group_and_day

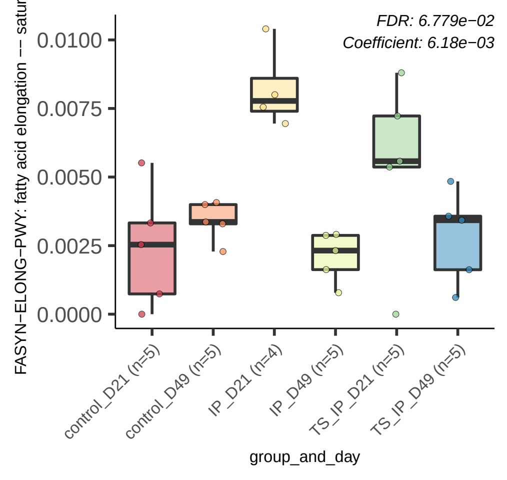
FDR: 6.779e−02
Coefficient: 6.18e−03
FASYN−ELONG−PWY: fatty acid elongation -- satur
0.0100
0.0075
0.0050
0.0025
0.0000
control_D21 (n=5)
control_D49 (n=5)
IP_D21 (n=4)
IP_D49 (n=5)
TS_IP_D21 (n=5)
TS_IP_D49 (n=5)
group_and_day

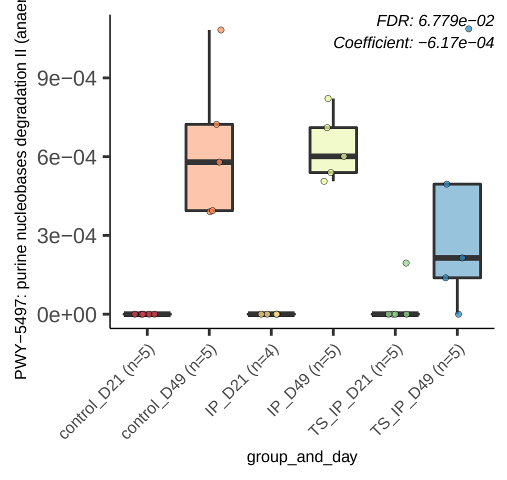

FDR: 6.779e−02
Coefficient: −6.17e−04
PWY−5497: purine nucleobases degradation II (anaer
9e−04
6e−04
3e−04
0e+00
control_D21 (n=5)
control_D49 (n=5)
IP_D21 (n=4)
IP_D49 (n=5)
TS_IP_D21 (n=5)
TS_IP_D49 (n=5)
group_and_day

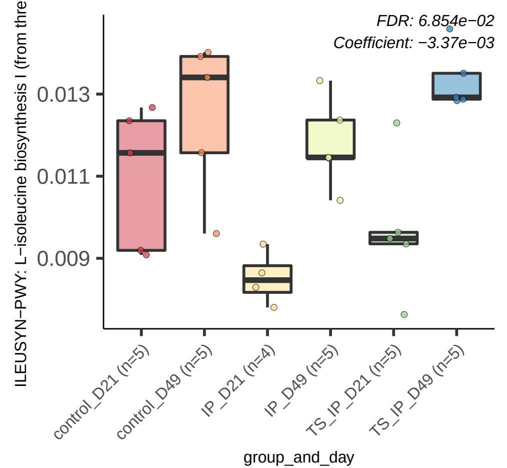

FDR: 6.854e−02
Coefficient: −3.37e−03
ILEUSYN−PWY: L−isoleucine biosynthesis I (from thre
0.013
0.011
0.009
control_D21 (n=5)
control_D49 (n=5)
IP_D21 (n=4)
IP_D49 (n=5)
TS_IP_D21 (n=5)
TS_IP_D49 (n=5)
group_and_day

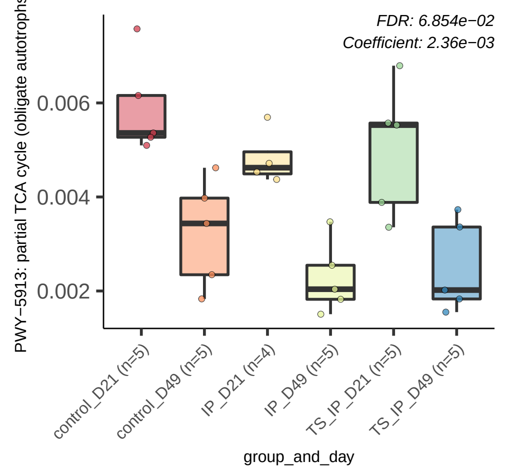
FDR: 6.854e−02
Coefficient: 2.36e−03
PWY−5913: partial TCA cycle (obligate autotrophs
0.006
0.004
0.002
control_D21 (n=5)
control_D49 (n=5)
IP_D21 (n=4)
IP_D49 (n=5)
TS_IP_D21 (n=5)
TS_IP_D49 (n=5)
group_and_day

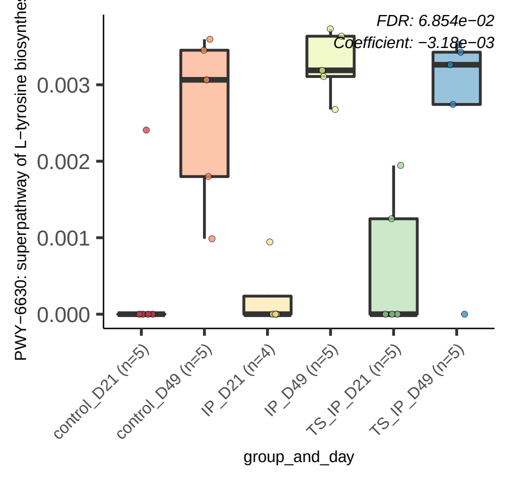

FDR: 6.854e−02
Coefficient: −3.18e−03
PWY−6630: superpathway of L−tyrosine biosynthes
0.003
0.002
0.001
0.000
control_D21 (n=5)
control_D49 (n=5)
IP_D21 (n=4)
IP_D49 (n=5)
TS_IP_D21 (n=5)
TS_IP_D49 (n=5)
group_and_day

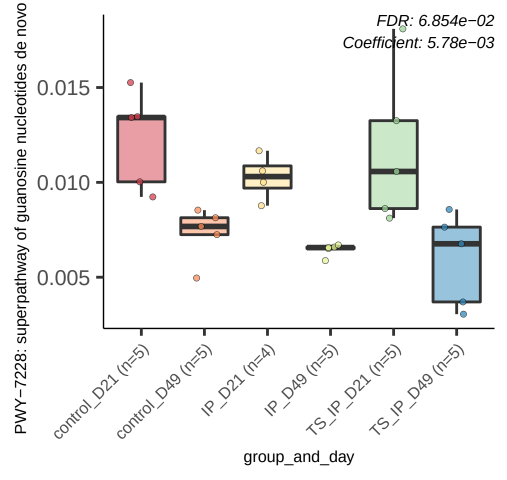
FDR: 6.854e−02
Coefficient: 5.78e−03
PWY−7228: superpathway of guanosine nucleotides de novo
0.015
0.010
0.005
control_D21 (n=5)
control_D49 (n=5)
IP_D21 (n=4)
IP_D49 (n=5)
TS_IP_D21 (n=5)
TS_IP_D49 (n=5)
group_and_day

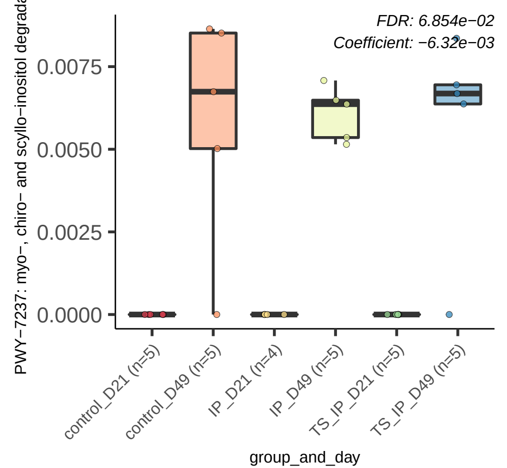
FDR: 6.854e−02
Coefficient: −6.32e−03
PWY−7237: myo−, chiro− and scyllo−inositol degrada
0.0075
0.0050
0.0025
0.0000
control_D21 (n=5)
control_D49 (n=5)
IP_D21 (n=4)
IP_D49 (n=5)
TS_IP_D21 (n=5)
TS_IP_D49 (n=5)
group_and_day

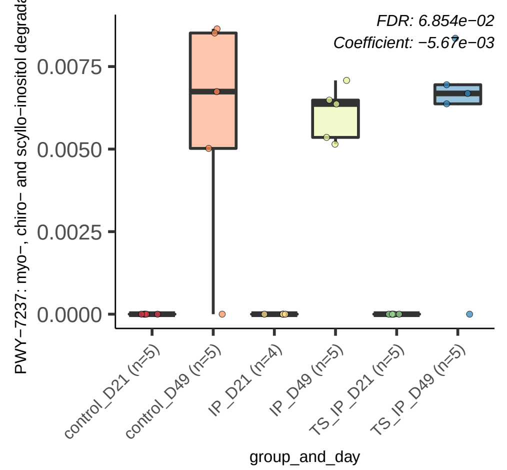
FDR: 6.854e−02
Coefficient: −5.67e−03
PWY−7237: myo−, chiro− and scyllo−inositol degrada
0.0075
0.0050
0.0025
0.0000
control_D21 (n=5)
control_D49 (n=5)
IP_D21 (n=4)
IP_D49 (n=5)
TS_IP_D21 (n=5)
TS_IP_D49 (n=5)
group_and_day

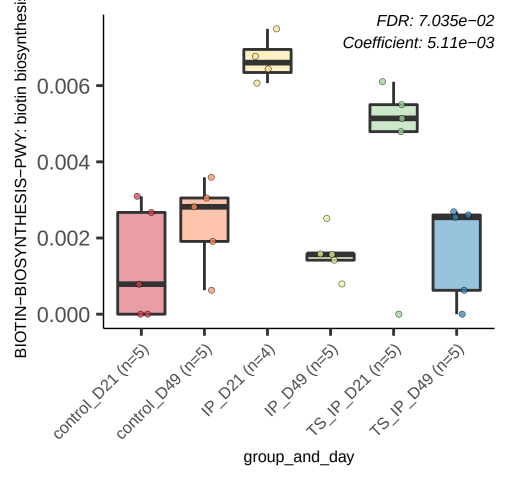
FDR: 7.035e−02
Coefficient: 5.11e−03
BIOTIN−BIOSYNTHESIS−PWY: biotin biosynthesis
0.000
0.002
0.004
0.006
control_D21 (n=5)
control_D49 (n=5)
IP_D21 (n=4)
IP_D49 (n=5)
TS_IP_D21 (n=5)
TS_IP_D49 (n=5)
group_and_day

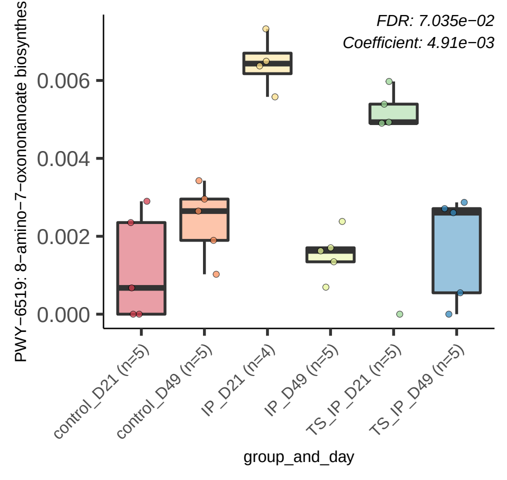
FDR: 7.035e−02
Coefficient: 4.91e−03
PWY−6519: 8−amino−7−oxononanoate biosynthes
0.000
0.002
0.004
0.006
control_D21 (n=5)
control_D49 (n=5)
IP_D21 (n=4)
IP_D49 (n=5)
TS_IP_D21 (n=5)
TS_IP_D49 (n=5)
group_and_day

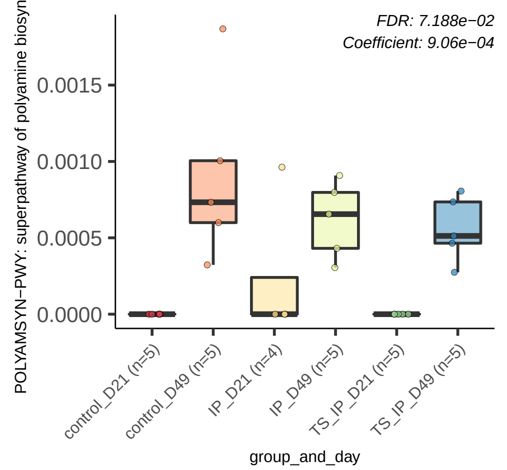

FDR: 7.188e−02
Coefficient: 9.06e−04
POLYAMSYN−PWY: superpathway of polyamine biosyn
0.0015
0.0010
0.0005
0.0000
control_D21 (n=5)
control_D49 (n=5)
IP_D21 (n=4)
IP_D49 (n=5)
TS_IP_D21 (n=5)
TS_IP_D49 (n=5)
group_and_day

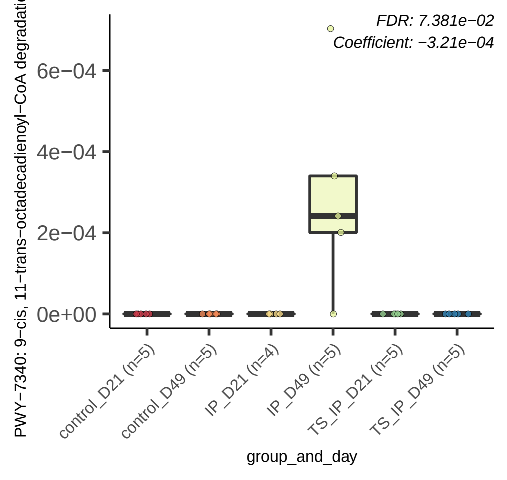
FDR: 7.381e−02
Coefficient: −3.21e−04
PWY−7340: 9−cis, 11−trans−octadecadienoyl−CoA degradatio
6e−04
4e−04
2e−04
0e+00
control_D21 (n=5)
control_D49 (n=5)
IP_D21 (n=4)
IP_D49 (n=5)
TS_IP_D21 (n=5)
TS_IP_D49 (n=5)
group_and_day

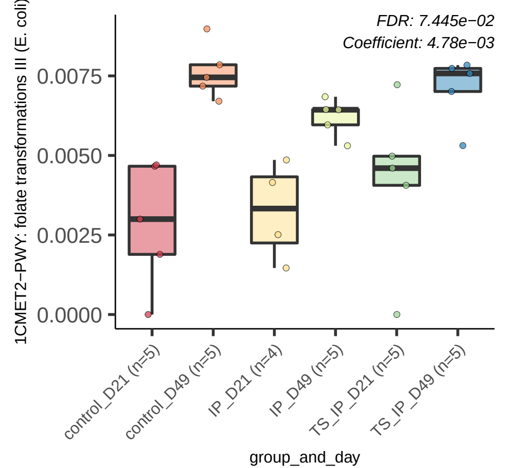
FDR: 7.445e−02
Coefficient: 4.78e−03
1CMET2−PWY: folate transformations III (E. coli)
0.0075
0.0050
0.0025
0.0000
control_D21 (n=5)
control_D49 (n=5)
IP_D21 (n=4)
IP_D49 (n=5)
TS_IP_D21 (n=5)
TS_IP_D49 (n=5)
group_and_day

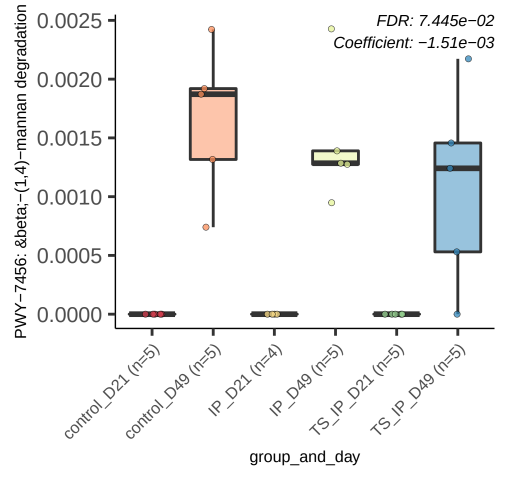
FDR: 7.445e−02
Coefficient: −1.51e−03
PWY−7456: β−(1,4)−mannan degradation
0.0025
0.0020
0.0015
0.0010
0.0005
0.0000
control_D21 (n=5)
control_D49 (n=5)
IP_D21 (n=4)
IP_D49 (n=5)
TS_IP_D21 (n=5)
TS_IP_D49 (n=5)
group_and_day

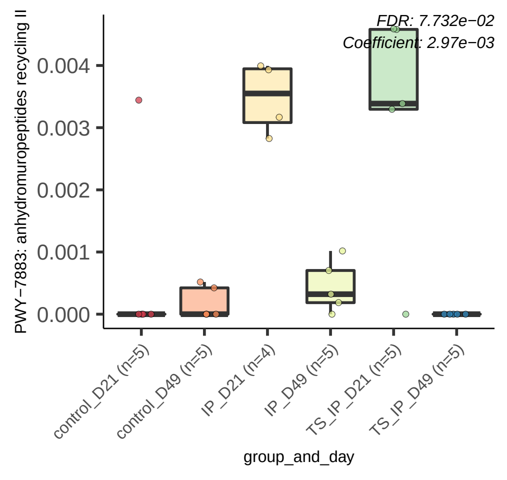

FDR: 7.732e−02
Coefficient: 2.97e−03
PWY−7883: anhydromuropeptides recycling II
0.004
0.003
0.002
0.001
0.000
control_D21 (n=5)
control_D49 (n=5)
IP_D21 (n=4)
IP_D49 (n=5)
TS_IP_D21 (n=5)
TS_IP_D49 (n=5)
group_and_day

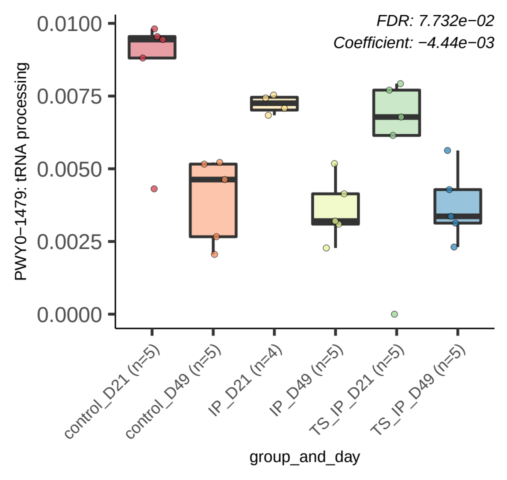
FDR: 7.732e−02
Coefficient: −4.44e−03
PWY0−1479: tRNA processing
0.0100
0.0075
0.0050
0.0025
0.0000
control_D21 (n=5)
control_D49 (n=5)
IP_D21 (n=4)
IP_D49 (n=5)
TS_IP_D21 (n=5)
TS_IP_D49 (n=5)
group_and_day

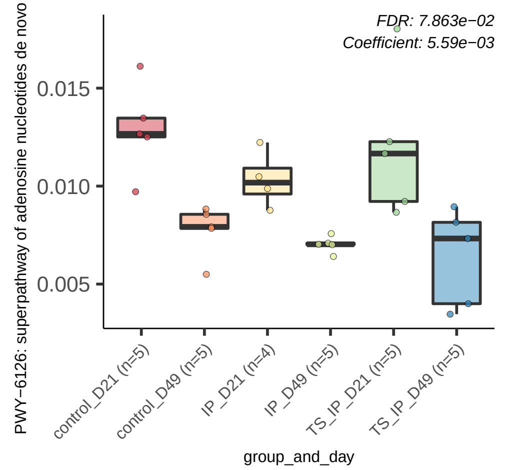
FDR: 7.863e−02
Coefficient: 5.59e−03
PWY−6126: superpathway of adenosine nucleotides de novo
0.015
0.010
0.005
control_D21 (n=5)
control_D49 (n=5)
IP_D21 (n=4)
IP_D49 (n=5)
TS_IP_D21 (n=5)
TS_IP_D49 (n=5)
group_and_day

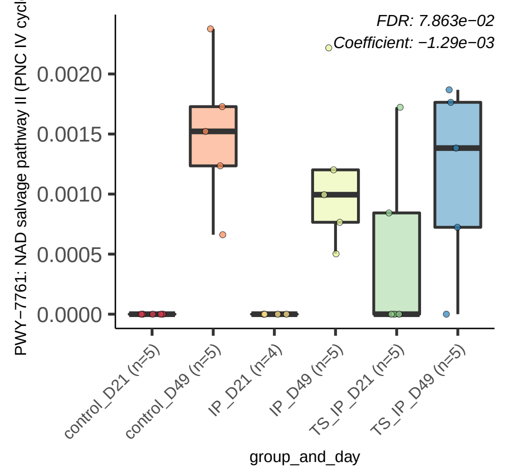

FDR: 7.863e−02
Coefficient: −1.29e−03
PWY−7761: NAD salvage pathway II (PNC IV cycl
0.0020
0.0015
0.0010
0.0005
0.0000
control_D21 (n=5)
control_D49 (n=5)
IP_D21 (n=4)
IP_D49 (n=5)
TS_IP_D21 (n=5)
TS_IP_D49 (n=5)
group_and_day

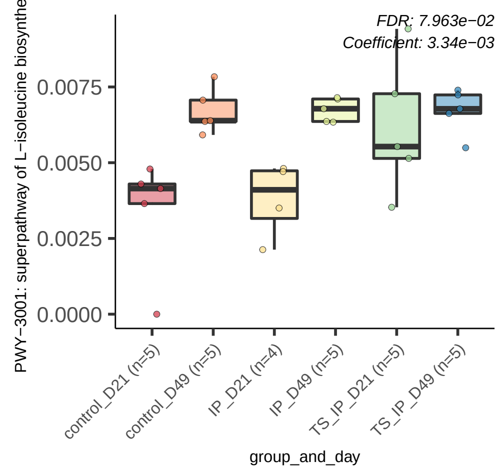
FDR: 7.963e−02
Coefficient: 3.34e−03
PWY−3001: superpathway of L−isoleucine biosynthe
0.0075
0.0050
0.0025
0.0000
control_D21 (n=5)
control_D49 (n=5)
IP_D21 (n=4)
IP_D49 (n=5)
TS_IP_D21 (n=5)
TS_IP_D49 (n=5)
group_and_day

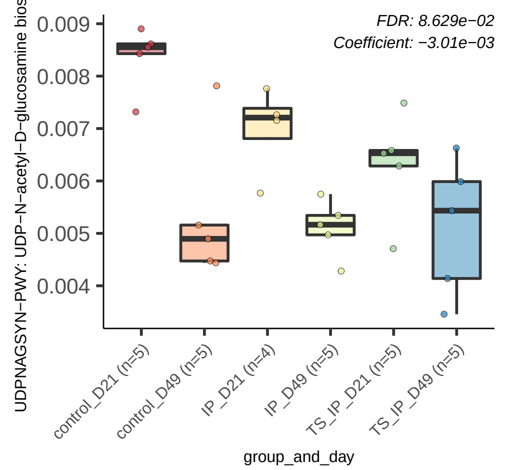
FDR: 8.629e−02
Coefficient: −3.01e−03
UDPNAGSYN−PWY: UDP−N−acetyl−D−glucosamine bios
0.009
0.008
0.007
0.006
0.005
0.004
control_D21 (n=5)
control_D49 (n=5)
IP_D21 (n=4)
IP_D49 (n=5)
TS_IP_D21 (n=5)
TS_IP_D49 (n=5)
group_and_day

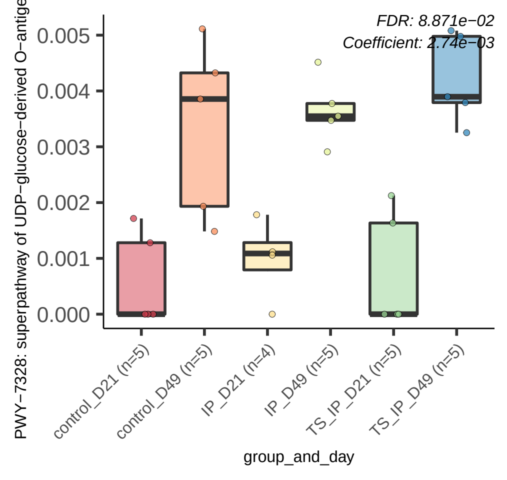

FDR: 8.871e−02
Coefficient: 2.74e−03
PWY−7328: superpathway of UDP−glucose−derived O−antige
0.005
0.004
0.003
0.002
0.001
0.000
control_D21 (n=5)
control_D49 (n=5)
IP_D21 (n=4)
IP_D49 (n=5)
TS_IP_D21 (n=5)
TS_IP_D49 (n=5)
group_and_day

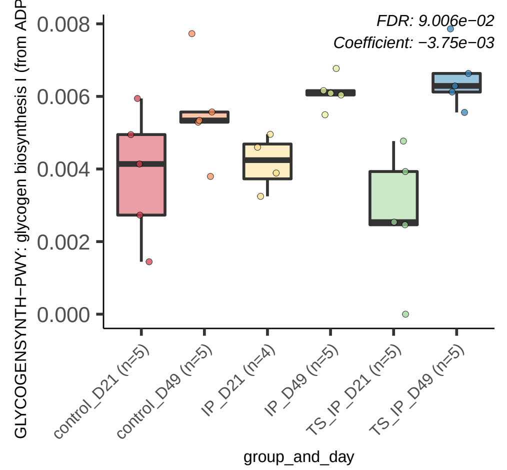

FDR: 9.006e−02
Coefficient: −3.75e−03
GLYCOGENSYNTH−PWY: glycogen biosynthesis I (from ADP
0.008
0.006
0.004
0.002
0.000
control_D21 (n=5)
control_D49 (n=5)
IP_D21 (n=4)
IP_D49 (n=5)
TS_IP_D21 (n=5)
TS_IP_D49 (n=5)
group_and_day

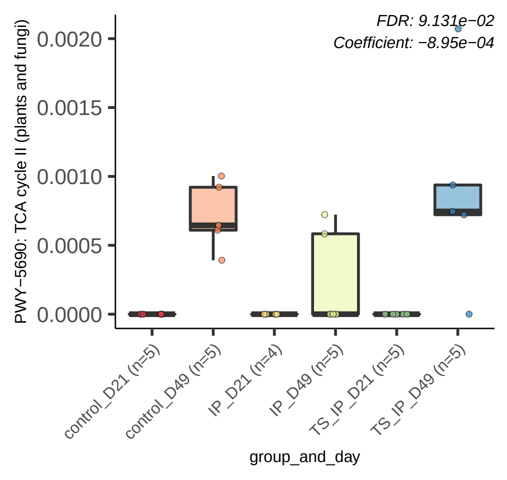

FDR: 9.131e−02
Coefficient: −8.95e−04
PWY−5690: TCA cycle II (plants and fungi)
0.0020
0.0015
0.0010
0.0005
0.0000
control_D21 (n=5)
control_D49 (n=5)
IP_D21 (n=4)
IP_D49 (n=5)
TS_IP_D21 (n=5)
TS_IP_D49 (n=5)
group_and_day

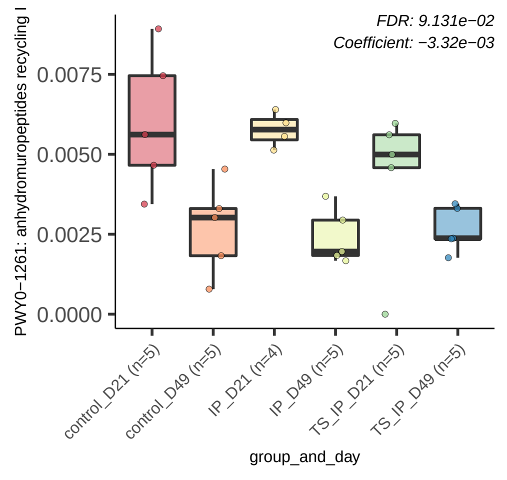
FDR: 9.131e−02
Coefficient: −3.32e−03
PWY0−1261: anhydromuropeptides recycling I
0.0075
0.0050
0.0025
0.0000
control_D21 (n=5)
control_D49 (n=5)
IP_D21 (n=4)
IP_D49 (n=5)
TS_IP_D21 (n=5)
TS_IP_D49 (n=5)
group_and_day

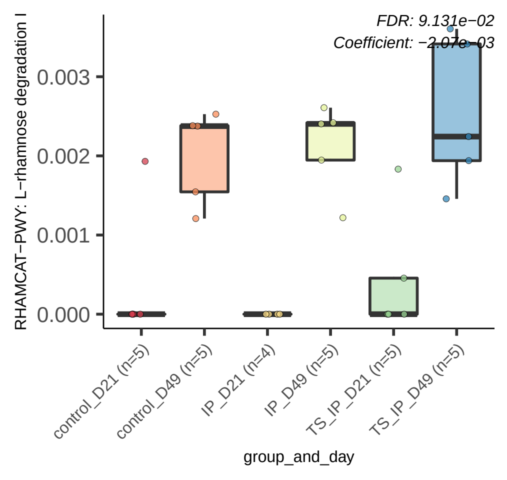
FDR: 9.131e−02
Coefficient: −2.07e−03
RHAMCAT−PWY: L−rhamnose degradation I
0.003
0.002
0.001
0.000
control_D21 (n=5)
control_D49 (n=5)
IP_D21 (n=4)
IP_D49 (n=5)
TS_IP_D21 (n=5)
TS_IP_D49 (n=5)
group_and_day

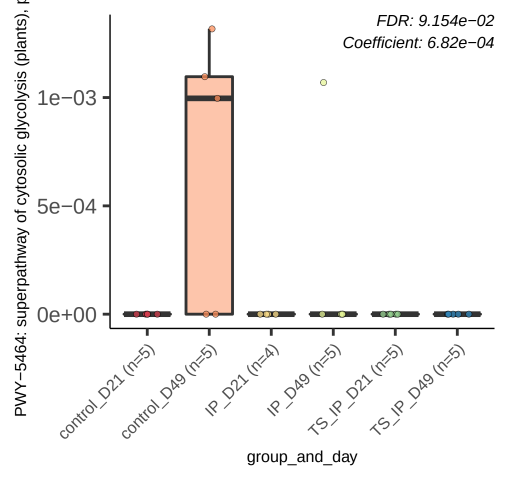
FDR: 9.154e−02
Coefficient: 6.82e−04
PWY−5464: superpathway of cytosolic glycolysis (plants), p
1e−03
5e−04
0e+00
control_D21 (n=5)
control_D49 (n=5)
IP_D21 (n=4)
IP_D49 (n=5)
TS_IP_D21 (n=5)
TS_IP_D49 (n=5)
group_and_day

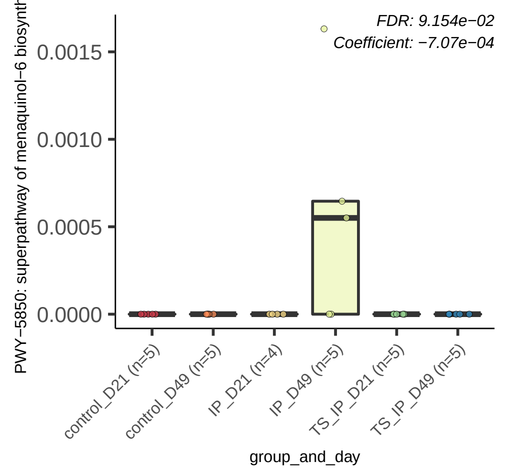
FDR: 9.154e−02
Coefficient: −7.07e−04
PWY−5850: superpathway of menaquinol−6 biosynth
0.0015
0.0010
0.0005
0.0000
control_D21 (n=5)
control_D49 (n=5)
IP_D21 (n=4)
IP_D49 (n=5)
TS_IP_D21 (n=5)
TS_IP_D49 (n=5)
group_and_day

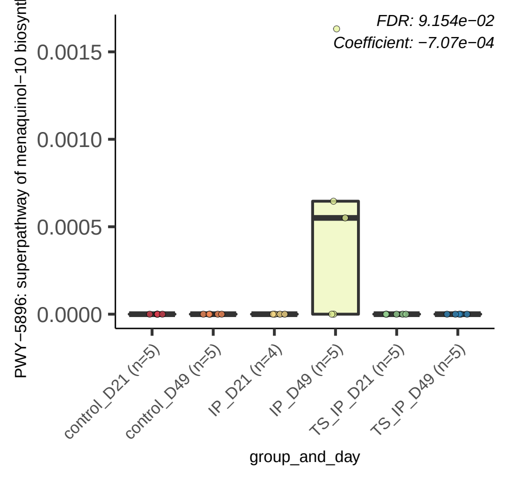

FDR: 9.154e−02
Coefficient: −7.07e−04
PWY−5896: superpathway of menaquinol−10 biosynt
0.0015
0.0010
0.0005
0.0000
control_D21 (n=5)
control_D49 (n=5)
IP_D21 (n=4)
IP_D49 (n=5)
TS_IP_D21 (n=5)
TS_IP_D49 (n=5)
group_and_day

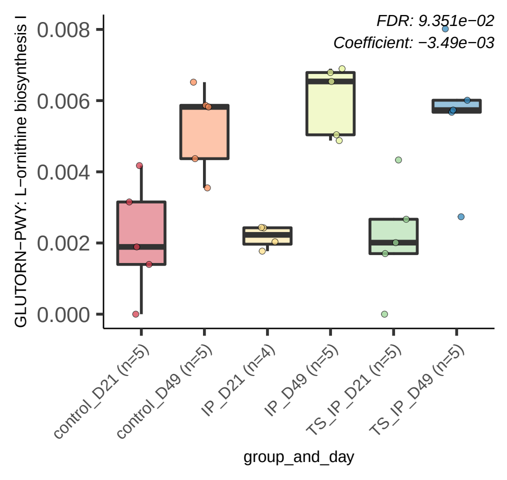
FDR: 9.351e−02
Coefficient: −3.49e−03
GLUTORN−PWY: L−ornithine biosynthesis I
0.008
0.006
0.004
0.002
0.000
control_D21 (n=5)
control_D49 (n=5)
IP_D21 (n=4)
IP_D49 (n=5)
TS_IP_D21 (n=5)
TS_IP_D49 (n=5)
group_and_day

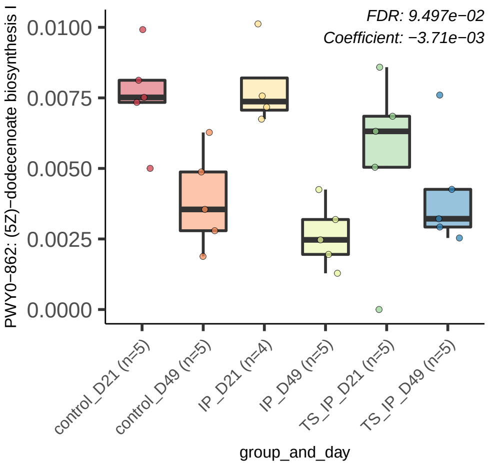

FDR: 9.497e−02
Coefficient: −3.71e−03
PWY0−862: (5Z)−dodecenoate biosynthesis I
0.0100
0.0075
0.0050
0.0025
0.0000
control_D21 (n=5)
control_D49 (n=5)
IP_D21 (n=4)
IP_D49 (n=5)
TS_IP_D21 (n=5)
TS_IP_D49 (n=5)
group_and_day

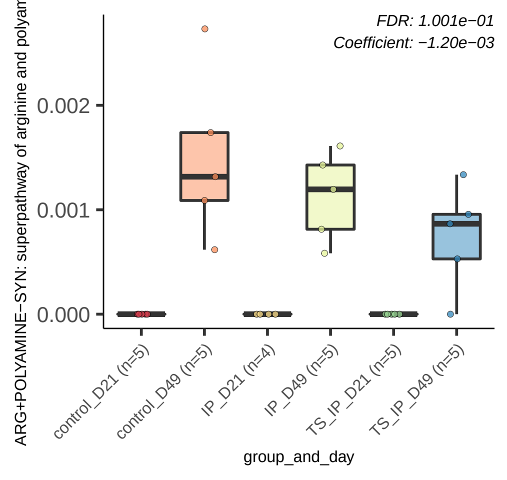

FDR: 1.001e−01
Coefficient: −1.20e−03
ARG+POLYAMINE−SYN: superpathway of arginine and polyam
0.002
0.001
0.000
control_D21 (n=5)
control_D49 (n=5)
IP_D21 (n=4)
IP_D49 (n=5)
TS_IP_D21 (n=5)
TS_IP_D49 (n=5)
group_and_day

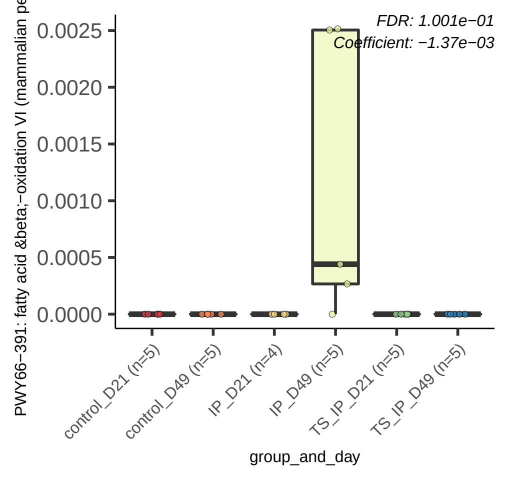
FDR: 1.001e−01
Coefficient: −1.37e−03
PWY66−391: fatty acid β−oxidation VI (mammalian pe
0.0025
0.0020
0.0015
0.0010
0.0005
0.0000
control_D21 (n=5)
control_D49 (n=5)
IP_D21 (n=4)
IP_D49 (n=5)
TS_IP_D21 (n=5)
TS_IP_D49 (n=5)
group_and_day

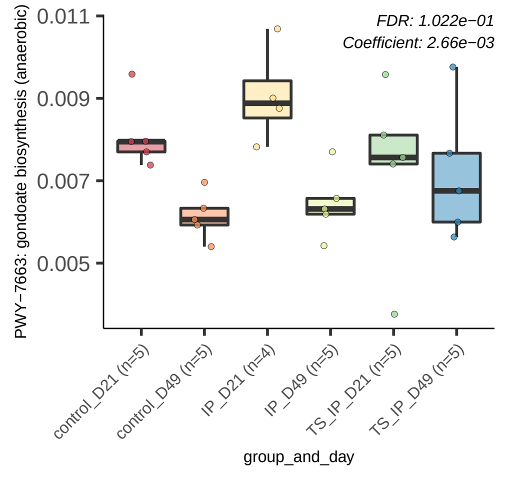

FDR: 1.022e−01
Coefficient: 2.66e−03
PWY−7663: gondoate biosynthesis (anaerobic)
0.011
0.009
0.007
0.005
control_D21 (n=5)
control_D49 (n=5)
IP_D21 (n=4)
IP_D49 (n=5)
TS_IP_D21 (n=5)
TS_IP_D49 (n=5)
group_and_day

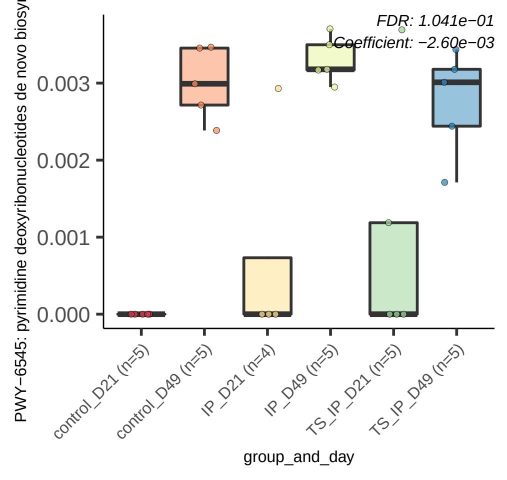

FDR: 1.041e−01
Coefficient: −2.60e−03
PWY−6545: pyrimidine deoxyribonucleotides de novo biosy
0.003
0.002
0.001
0.000
control_D21 (n=5)
control_D49 (n=5)
IP_D21 (n=4)
IP_D49 (n=5)
TS_IP_D21 (n=5)
TS_IP_D49 (n=5)
group_and_day

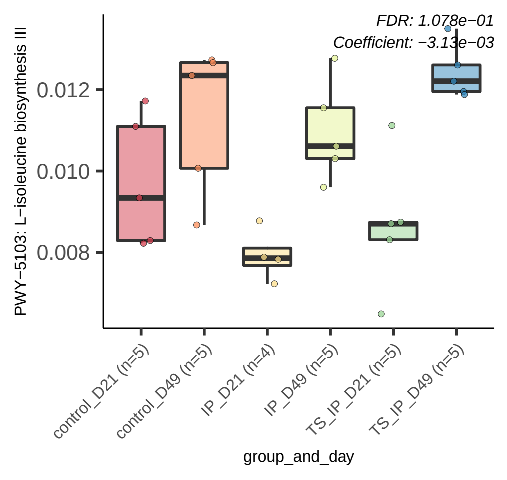
FDR: 1.078e−01
Coefficient: −3.13e−03
PWY−5103: L−isoleucine biosynthesis III
0.012
0.010
0.008
control_D21 (n=5)
control_D49 (n=5)
IP_D21 (n=4)
IP_D49 (n=5)
TS_IP_D21 (n=5)
TS_IP_D49 (n=5)
group_and_day

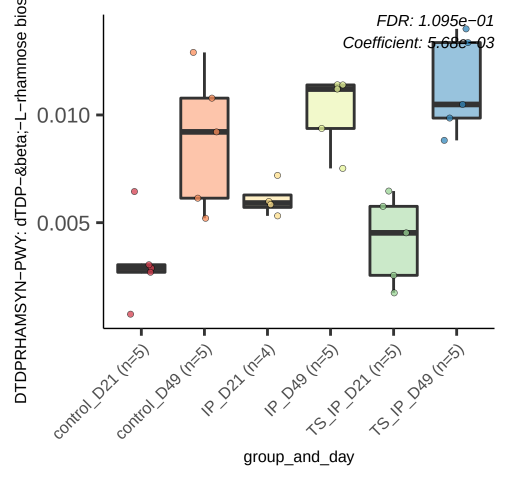
FDR: 1.095e−01
Coefficient: 5.68e−03
DTDPRHAMSYN−PWY: dTDP−β−L−rhamnose bios
0.010
0.005
control_D21 (n=5)
control_D49 (n=5)
IP_D21 (n=4)
IP_D49 (n=5)
TS_IP_D21 (n=5)
TS_IP_D49 (n=5)
group_and_day

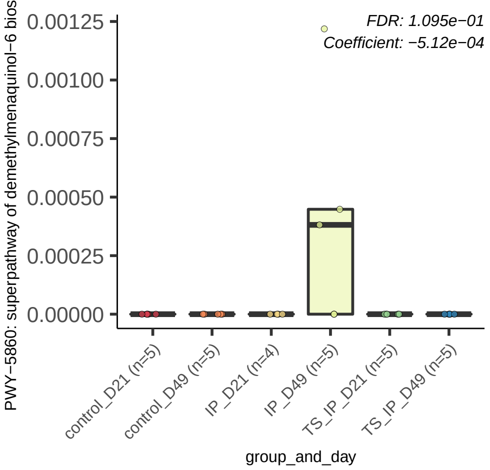

FDR: 1.095e−01
Coefficient: −5.12e−04
PWY−5860: superpathway of demethylmenaquinol−6 bios
0.00125
0.00100
0.00075
0.00050
0.00025
0.00000
control_D21 (n=5)
control_D49 (n=5)
IP_D21 (n=4)
IP_D49 (n=5)
TS_IP_D21 (n=5)
TS_IP_D49 (n=5)
group_and_day

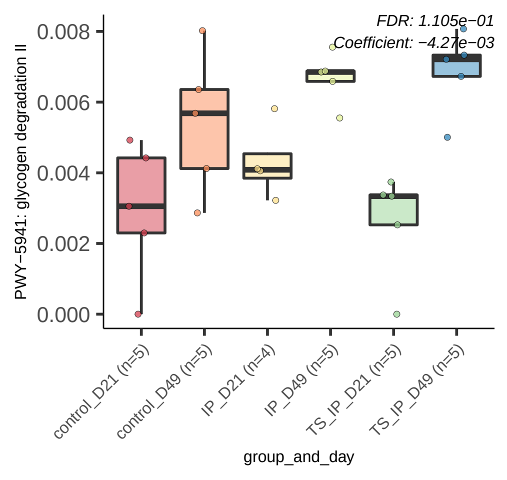
FDR: 1.105e−01
Coefficient: −4.27e−03
PWY−5941: glycogen degradation II
0.000
0.002
0.004
0.006
0.008
control_D21 (n=5)
control_D49 (n=5)
IP_D21 (n=4)
IP_D49 (n=5)
TS_IP_D21 (n=5)
TS_IP_D49 (n=5)
group_and_day

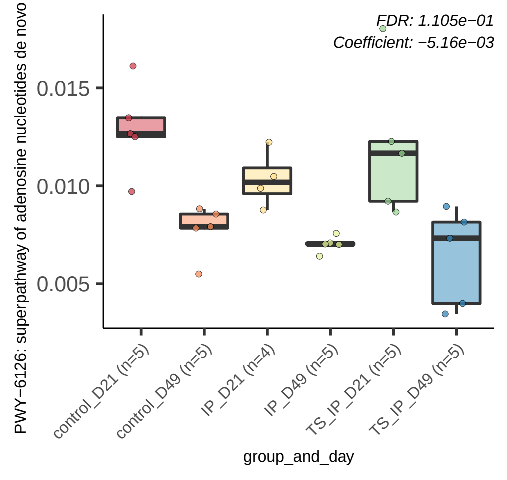

FDR: 1.105e−01
Coefficient: −5.16e−03
PWY−6126: superpathway of adenosine nucleotides de novo
0.015
0.010
0.005
control_D21 (n=5)
control_D49 (n=5)
IP_D21 (n=4)
IP_D49 (n=5)
TS_IP_D21 (n=5)
TS_IP_D49 (n=5)
group_and_day

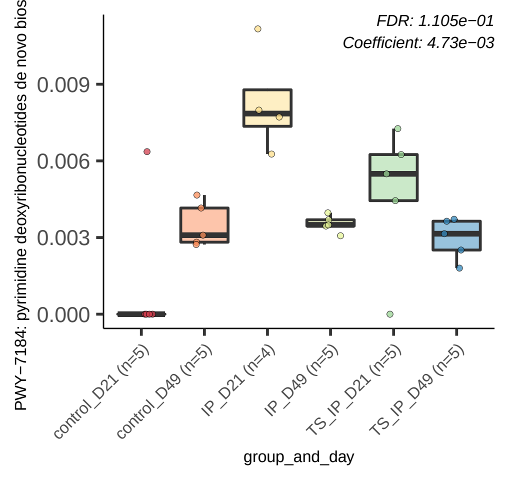
FDR: 1.105e−01
Coefficient: 4.73e−03
PWY−7184: pyrimidine deoxyribonucleotides de novo bios
0.009
0.006
0.003
0.000
control_D21 (n=5)
control_D49 (n=5)
IP_D21 (n=4)
IP_D49 (n=5)
TS_IP_D21 (n=5)
TS_IP_D49 (n=5)
group_and_day

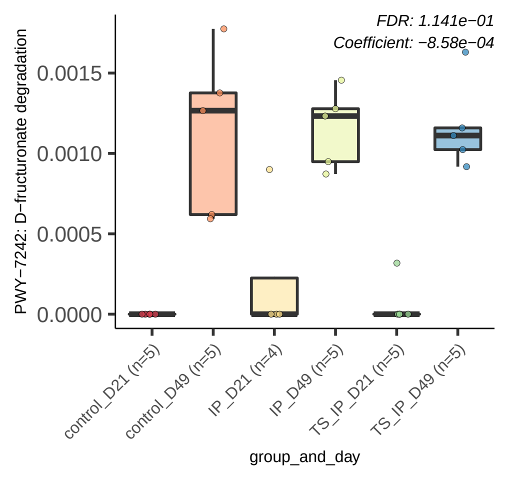
FDR: 1.141e−01
Coefficient: −8.58e−04
PWY−7242: D−fructuronate degradation
0.0015
0.0010
0.0005
0.0000
control_D21 (n=5)
control_D49 (n=5)
IP_D21 (n=4)
IP_D49 (n=5)
TS_IP_D21 (n=5)
TS_IP_D49 (n=5)
group_and_day

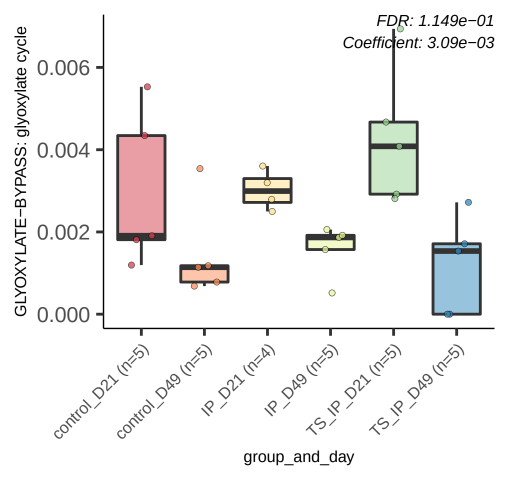
FDR: 1.149e−01
Coefficient: 3.09e−03
GLYOXYLATE−BYPASS: glyoxylate cycle
0.006
0.004
0.002
0.000
control_D21 (n=5)
control_D49 (n=5)
IP_D21 (n=4)
IP_D49 (n=5)
TS_IP_D21 (n=5)
TS_IP_D49 (n=5)
group_and_day

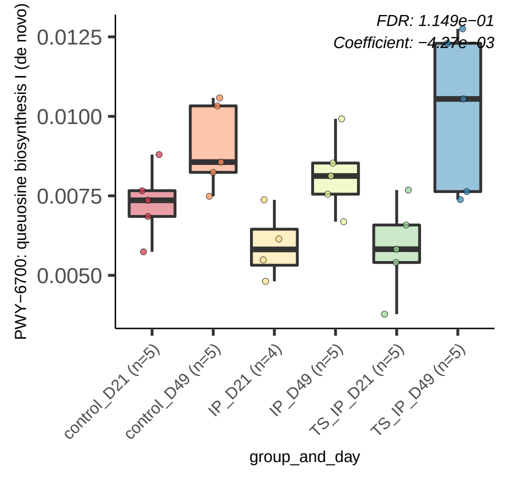

FDR: 1.149e−01
Coefficient: −4.27e−03
PWY−6700: queuosine biosynthesis I (de novo)
0.0125
0.0100
0.0075
0.0050
control_D21 (n=5)
control_D49 (n=5)
IP_D21 (n=4)
IP_D49 (n=5)
TS_IP_D21 (n=5)
TS_IP_D49 (n=5)
group_and_day

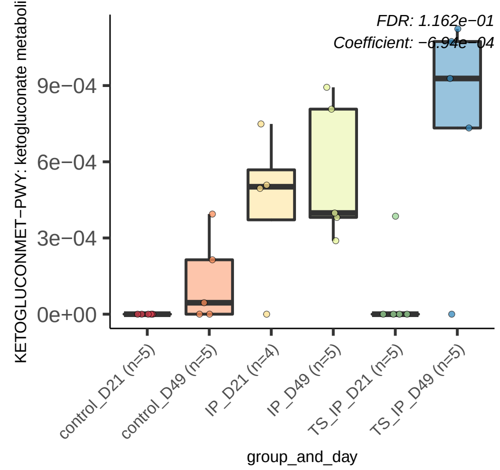

FDR: 1.162e−01
Coefficient: −6.94e−04
KETOGLUCONMET−PWY: ketogluconate metaboli
9e−04
6e−04
3e−04
0e+00
control_D21 (n=5)
control_D49 (n=5)
IP_D21 (n=4)
IP_D49 (n=5)
TS_IP_D21 (n=5)
TS_IP_D49 (n=5)
group_and_day

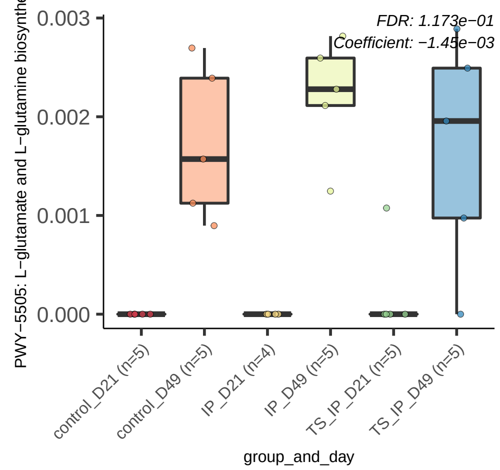

FDR: 1.173e−01
Coefficient: −1.45e−03
PWY−5505: L−glutamate and L−glutamine biosynthe
0.003
0.002
0.001
0.000
control_D21 (n=5)
control_D49 (n=5)
IP_D21 (n=4)
IP_D49 (n=5)
TS_IP_D21 (n=5)
TS_IP_D49 (n=5)
group_and_day

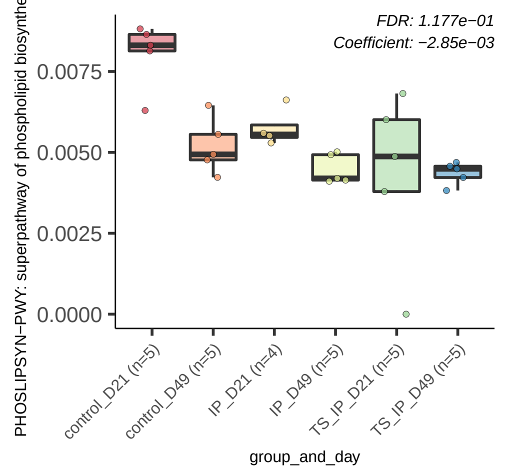
FDR: 1.177e−01
Coefficient: −2.85e−03
PHOSLIPSYN−PWY: superpathway of phospholipid biosynthe
0.0075
0.0050
0.0025
0.0000
control_D21 (n=5)
control_D49 (n=5)
IP_D21 (n=4)
IP_D49 (n=5)
TS_IP_D21 (n=5)
TS_IP_D49 (n=5)
group_and_day

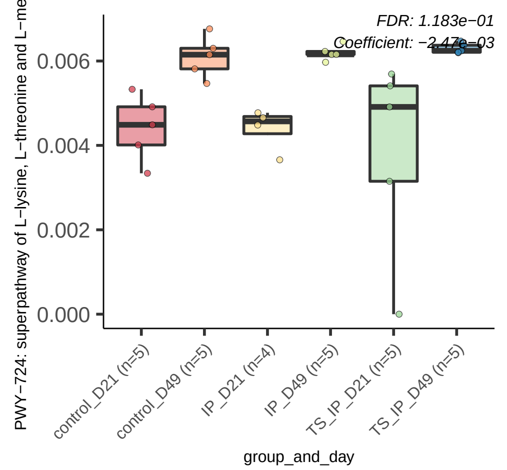
FDR: 1.183e−01
Coefficient: −2.47e−03
PWY−724: superpathway of L−lysine, L−threonine and L−me
0.000
0.002
0.004
0.006
control_D21 (n=5)
control_D49 (n=5)
IP_D21 (n=4)
IP_D49 (n=5)
TS_IP_D21 (n=5)
TS_IP_D49 (n=5)
group_and_day

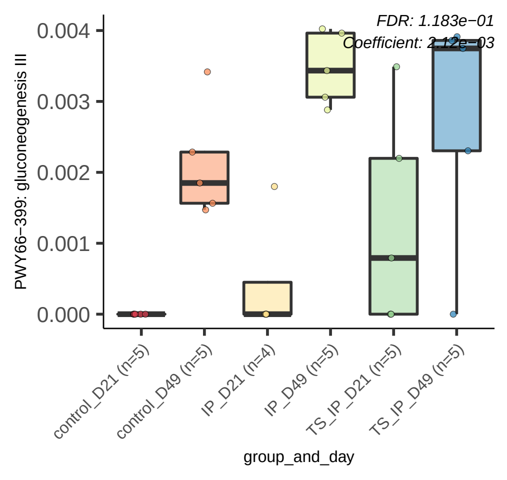

FDR: 1.183e−01
Coefficient: 2.12e−03
PWY66−399: gluconeogenesis III
0.004
0.003
0.002
0.001
0.000
control_D21 (n=5)
control_D49 (n=5)
IP_D21 (n=4)
IP_D49 (n=5)
TS_IP_D21 (n=5)
TS_IP_D49 (n=5)
group_and_day

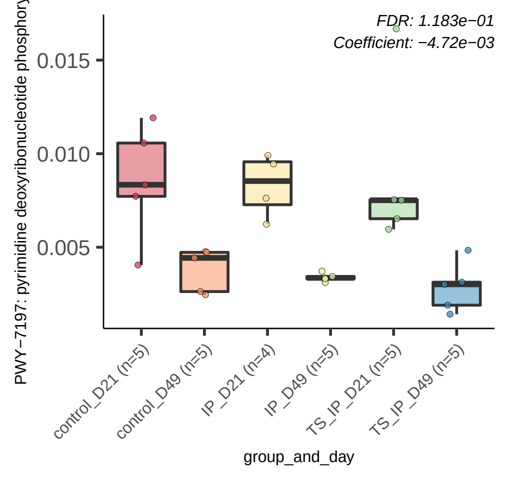

FDR: 1.183e−01
Coefficient: −4.72e−03
PWY−7197: pyrimidine deoxyribonucleotide phosphory
0.015
0.010
0.005
control_D21 (n=5)
control_D49 (n=5)
IP_D21 (n=4)
IP_D49 (n=5)
TS_IP_D21 (n=5)
TS_IP_D49 (n=5)
group_and_day

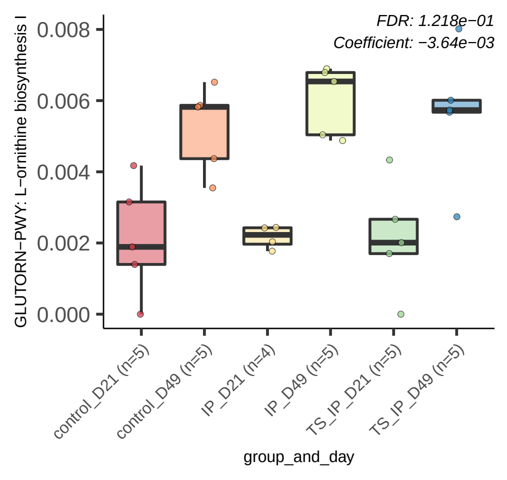
FDR: 1.218e−01
Coefficient: −3.64e−03
GLUTORN−PWY: L−ornithine biosynthesis I
0.008
0.006
0.004
0.002
0.000
control_D21 (n=5)
control_D49 (n=5)
IP_D21 (n=4)
IP_D49 (n=5)
TS_IP_D21 (n=5)
TS_IP_D49 (n=5)
group_and_day

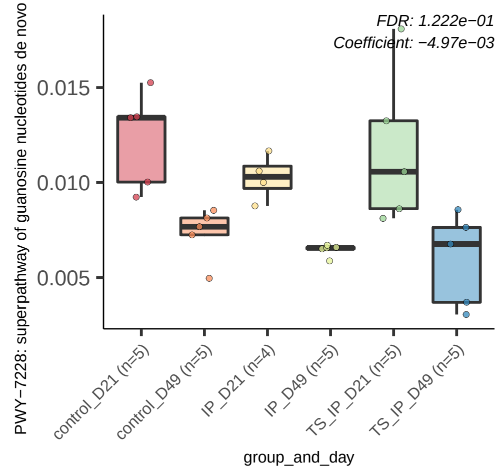

FDR: 1.222e−01
Coefficient: −4.97e−03
PWY−7228: superpathway of guanosine nucleotides de novo
0.015
0.010
0.005
control_D21 (n=5)
control_D49 (n=5)
IP_D21 (n=4)
IP_D49 (n=5)
TS_IP_D21 (n=5)
TS_IP_D49 (n=5)
group_and_day

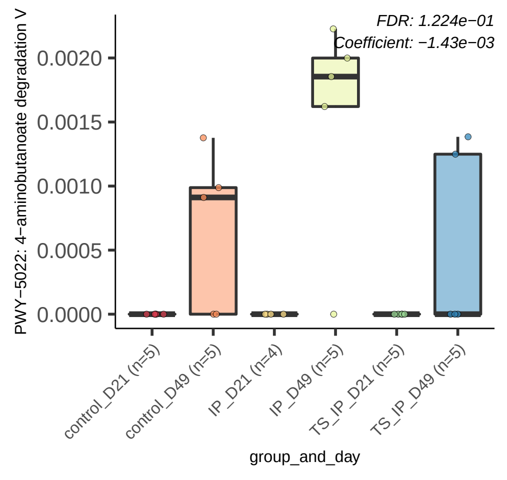
FDR: 1.224e−01
Coefficient: −1.43e−03
PWY−5022: 4−aminobutanoate degradation V
0.0020
0.0015
0.0010
0.0005
0.0000
control_D21 (n=5)
control_D49 (n=5)
IP_D21 (n=4)
IP_D49 (n=5)
TS_IP_D21 (n=5)
TS_IP_D49 (n=5)
group_and_day

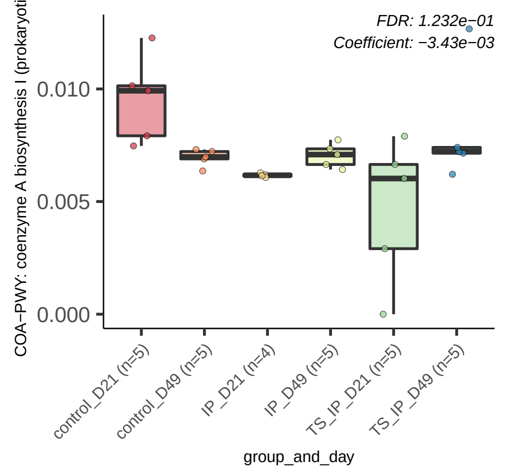
FDR: 1.232e−01
Coefficient: −3.43e−03
COA−PWY: coenzyme A biosynthesis I (prokaryoti
0.010
0.005
0.000
control_D21 (n=5)
control_D49 (n=5)
IP_D21 (n=4)
IP_D49 (n=5)
TS_IP_D21 (n=5)
TS_IP_D49 (n=5)
group_and_day

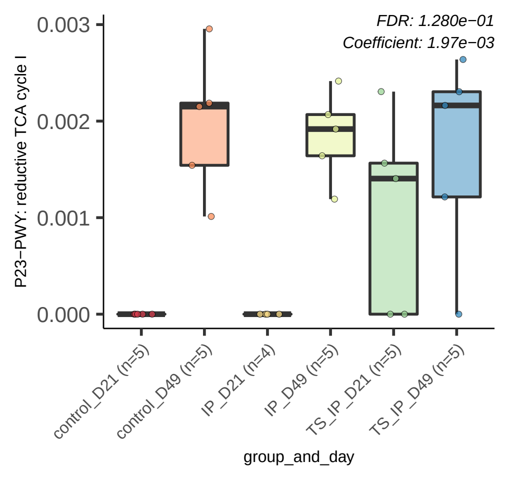
FDR: 1.280e−01
Coefficient: 1.97e−03
P23−PWY: reductive TCA cycle I
0.003
0.002
0.001
0.000
control_D21 (n=5)
control_D49 (n=5)
IP_D21 (n=4)
IP_D49 (n=5)
TS_IP_D21 (n=5)
TS_IP_D49 (n=5)
group_and_day

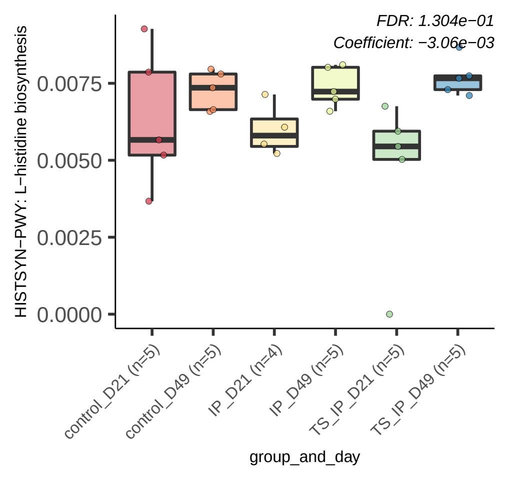

FDR: 1.304e−01
Coefficient: −3.06e−03
HISTSYN−PWY: L−histidine biosynthesis
0.0075
0.0050
0.0025
0.0000
control_D21 (n=5)
control_D49 (n=5)
IP_D21 (n=4)
IP_D49 (n=5)
TS_IP_D21 (n=5)
TS_IP_D49 (n=5)
group_and_day

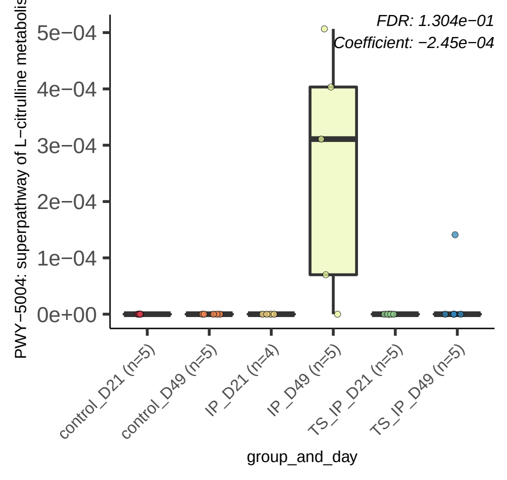

FDR: 1.304e−01
Coefficient: −2.45e−04
PWY−5004: superpathway of L−citrulline metabolis
5e−04
4e−04
3e−04
2e−04
1e−04
0e+00
control_D21 (n=5)
control_D49 (n=5)
IP_D21 (n=4)
IP_D49 (n=5)
TS_IP_D21 (n=5)
TS_IP_D49 (n=5)
group_and_day

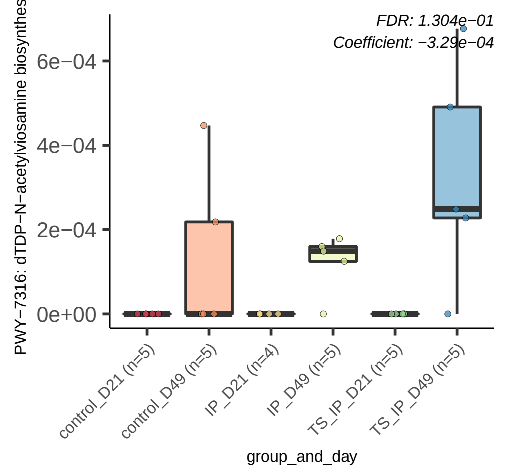

FDR: 1.304e−01
Coefficient: −3.29e−04
PWY−7316: dTDP−N−acetylviosamine biosynthes
6e−04
4e−04
2e−04
0e+00
control_D21 (n=5)
control_D49 (n=5)
IP_D21 (n=4)
IP_D49 (n=5)
TS_IP_D21 (n=5)
TS_IP_D49 (n=5)
group_and_day

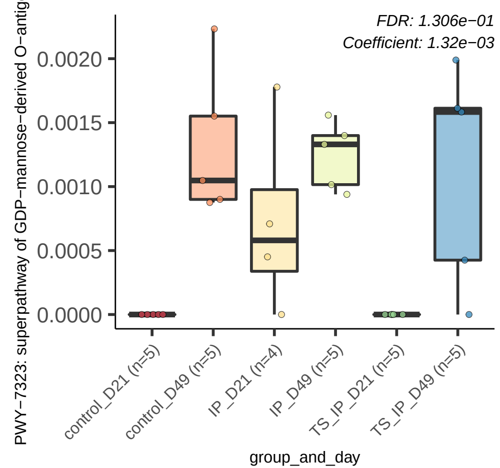
FDR: 1.306e−01
Coefficient: 1.32e−03
0.0020
0.0015
0.0010
0.0005
0.0000
PWY−7323: superpathway of GDP−mannose−derived O−antig
control_D21 (n=5)
control_D49 (n=5)
IP_D21 (n=4)
IP_D49 (n=5)
TS_IP_D21 (n=5)
TS_IP_D49 (n=5)
group_and_day

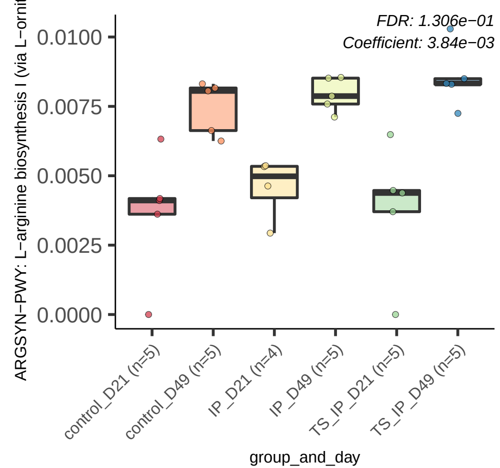

FDR: 1.306e−01
Coefficient: 3.84e−03
ARGSYN−PWY: L−arginine biosynthesis I (via L−ornit
0.0100
0.0075
0.0050
0.0025
0.0000
control_D21 (n=5)
control_D49 (n=5)
IP_D21 (n=4)
IP_D49 (n=5)
TS_IP_D21 (n=5)
TS_IP_D49 (n=5)
group_and_day

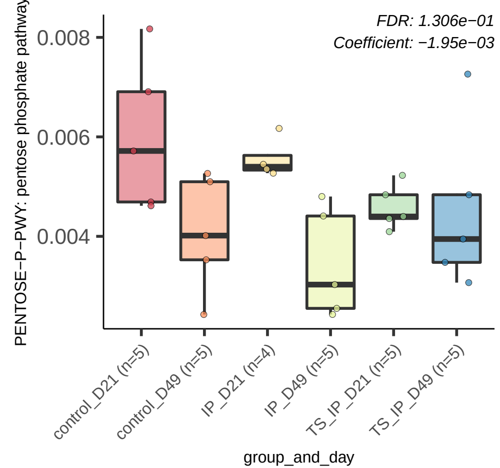

FDR: 1.306e−01
Coefficient: −1.95e−03
PENTOSE−P−PWY: pentose phosphate pathway
0.008
0.006
0.004
control_D21 (n=5)
control_D49 (n=5)
IP_D21 (n=4)
IP_D49 (n=5)
TS_IP_D21 (n=5)
TS_IP_D49 (n=5)
group_and_day

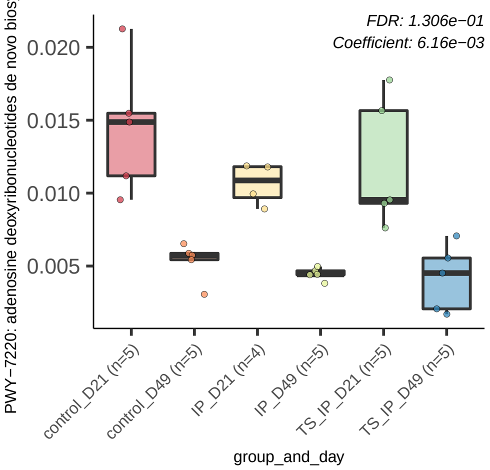

FDR: 1.306e−01
Coefficient: 6.16e−03
PWY−7220: adenosine deoxyribonucleotides de novo bios
0.020
0.015
0.010
0.005
control_D21 (n=5)
control_D49 (n=5)
IP_D21 (n=4)
IP_D49 (n=5)
TS_IP_D21 (n=5)
TS_IP_D49 (n=5)
group_and_day

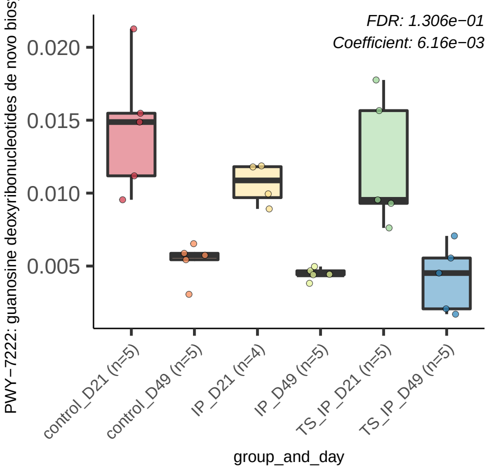
FDR: 1.306e−01
Coefficient: 6.16e−03
PWY−7222: guanosine deoxyribonucleotides de novo bios
0.020
0.015
0.010
0.005
control_D21 (n=5)
control_D49 (n=5)
IP_D21 (n=4)
IP_D49 (n=5)
TS_IP_D21 (n=5)
TS_IP_D49 (n=5)
group_and_day

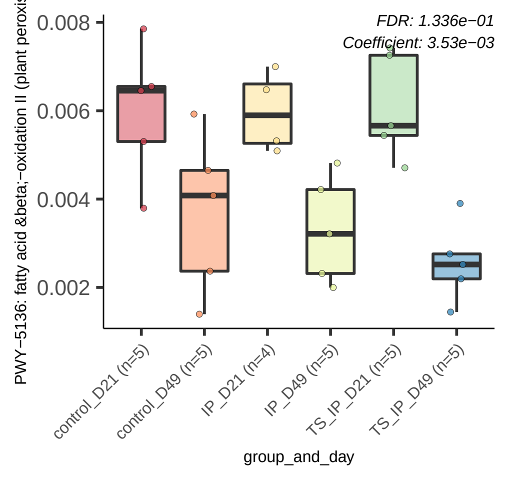

FDR: 1.336e−01
Coefficient: 3.53e−03
PWY−5136: fatty acid β−oxidation II (plant peroxis
0.008
0.006
0.004
0.002
control_D21 (n=5)
control_D49 (n=5)
IP_D21 (n=4)
IP_D49 (n=5)
TS_IP_D21 (n=5)
TS_IP_D49 (n=5)
group_and_day

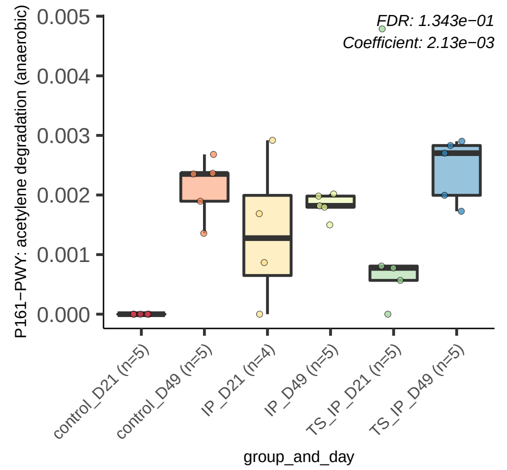

FDR: 1.343e−01
Coefficient: 2.13e−03
P161−PWY: acetylene degradation (anaerobic)
0.005
0.004
0.003
0.002
0.001
0.000
control_D21 (n=5)
control_D49 (n=5)
IP_D21 (n=4)
IP_D49 (n=5)
TS_IP_D21 (n=5)
TS_IP_D49 (n=5)
group_and_day

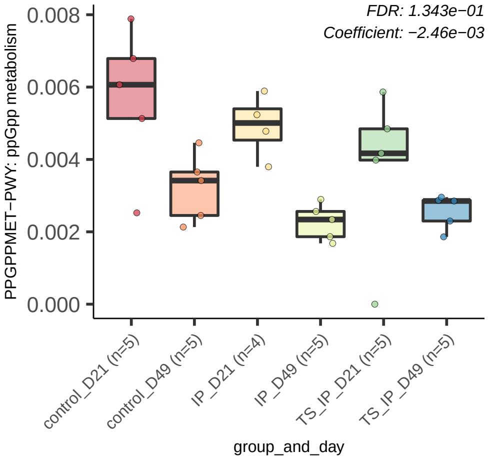

FDR: 1.343e−01
Coefficient: −2.46e−03
PPGPPMET−PWY: ppGpp metabolism
0.008
0.006
0.004
0.002
0.000
control_D21 (n=5)
control_D49 (n=5)
IP_D21 (n=4)
IP_D49 (n=5)
TS_IP_D21 (n=5)
TS_IP_D49 (n=5)
group_and_day

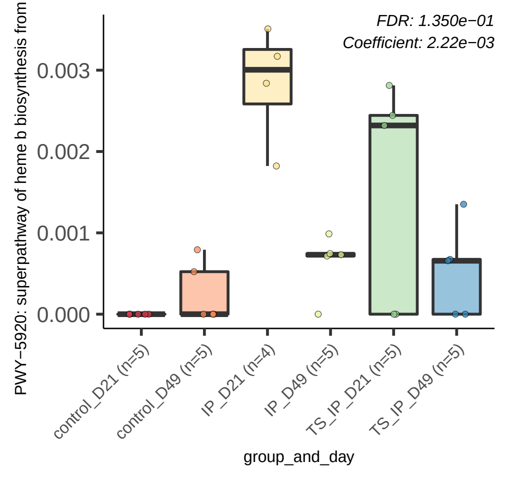

FDR: 1.350e−01
Coefficient: 2.22e−03
PWY−5920: superpathway of heme b biosynthesis from
0.000
0.001
0.002
0.003
control_D21 (n=5)
control_D49 (n=5)
IP_D21 (n=4)
IP_D49 (n=5)
TS_IP_D21 (n=5)
TS_IP_D49 (n=5)
group_and_day

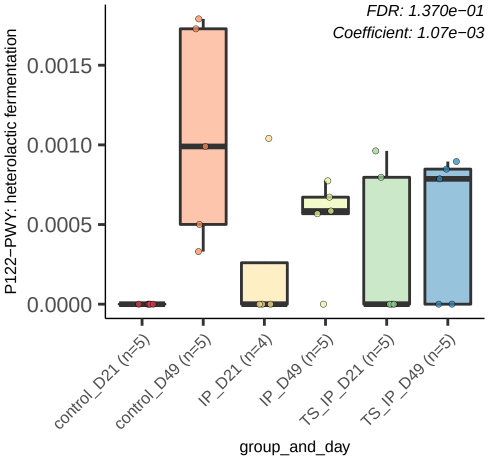
FDR: 1.370e−01
Coefficient: 1.07e−03
P122−PWY: heterolactic fermentation
0.0015
0.0010
0.0005
0.0000
control_D21 (n=5)
control_D49 (n=5)
IP_D21 (n=4)
IP_D49 (n=5)
TS_IP_D21 (n=5)
TS_IP_D49 (n=5)
group_and_day

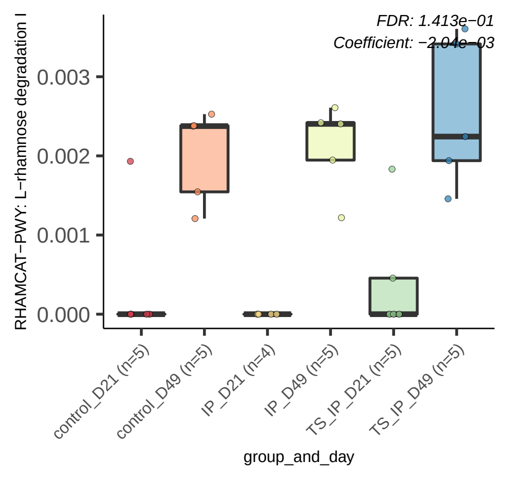
FDR: 1.413e−01
Coefficient: −2.04e−03
RHAMCAT−PWY: L−rhamnose degradation I
0.000
0.001
0.002
0.003
control_D21 (n=5)
control_D49 (n=5)
IP_D21 (n=4)
IP_D49 (n=5)
TS_IP_D21 (n=5)
TS_IP_D49 (n=5)
group_and_day

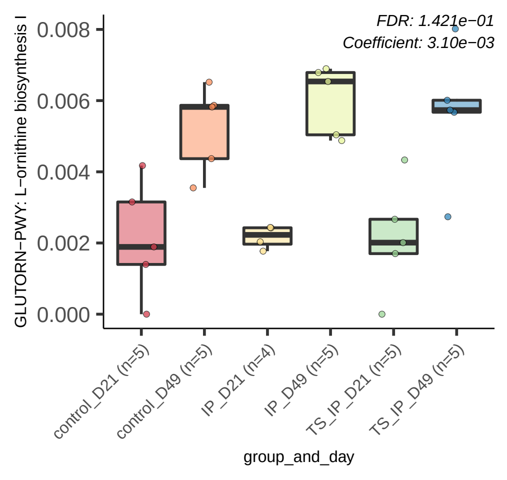

FDR: 1.421e−01
Coefficient: 3.10e−03
GLUTORN−PWY: L−ornithine biosynthesis I
0.008
0.006
0.004
0.002
0.000
control_D21 (n=5)
control_D49 (n=5)
IP_D21 (n=4)
IP_D49 (n=5)
TS_IP_D21 (n=5)
TS_IP_D49 (n=5)
group_and_day

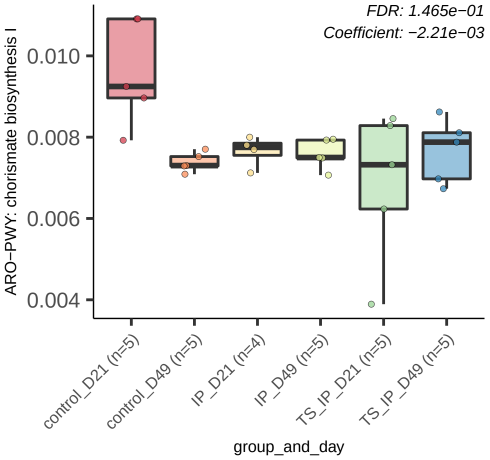
FDR: 1.465e−01
Coefficient: −2.21e−03
ARO−PWY: chorismate biosynthesis I
0.010
0.008
0.006
0.004
control_D21 (n=5)
control_D49 (n=5)
IP_D21 (n=4)
IP_D49 (n=5)
TS_IP_D21 (n=5)
TS_IP_D49 (n=5)
group_and_day

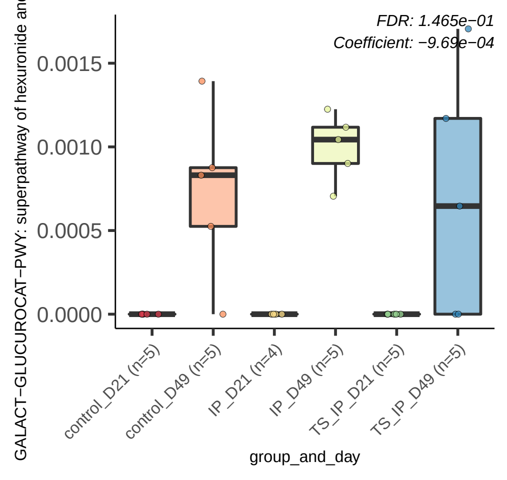

FDR: 1.465e−01
Coefficient: −9.69e−04
GALACT−GLUCUROCAT−PWY: superpathway of hexuronide an
0.0015
0.0010
0.0005
0.0000
control_D21 (n=5)
control_D49 (n=5)
IP_D21 (n=4)
IP_D49 (n=5)
TS_IP_D21 (n=5)
TS_IP_D49 (n=5)
group_and_day

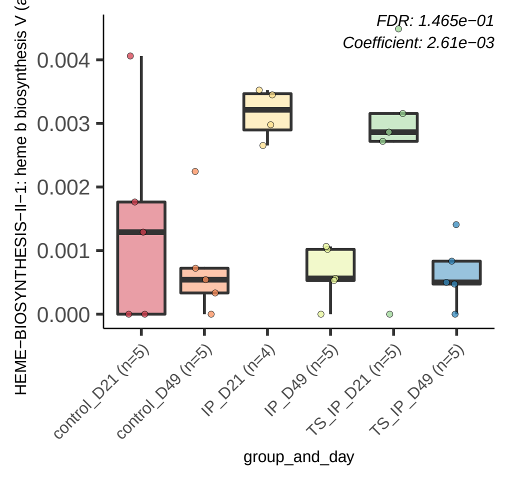

FDR: 1.465e−01
Coefficient: 2.61e−03
HEME−BIOSYNTHESIS−II−1: heme b biosynthesis V (a
0.004
0.003
0.002
0.001
0.000
control_D21 (n=5)
control_D49 (n=5)
IP_D21 (n=4)
IP_D49 (n=5)
TS_IP_D21 (n=5)
TS_IP_D49 (n=5)
group_and_day

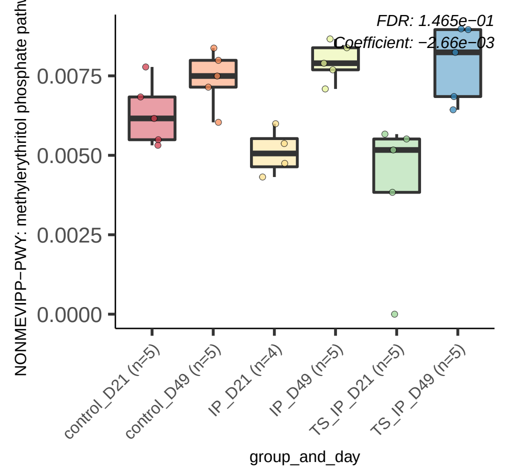

FDR: 1.465e−01
Coefficient: −2.66e−03
NONMEVIPP−PWY: methylerythritol phosphate pathw
0.0075
0.0050
0.0025
0.0000
control_D21 (n=5)
control_D49 (n=5)
IP_D21 (n=4)
IP_D49 (n=5)
TS_IP_D21 (n=5)
TS_IP_D49 (n=5)
group_and_day

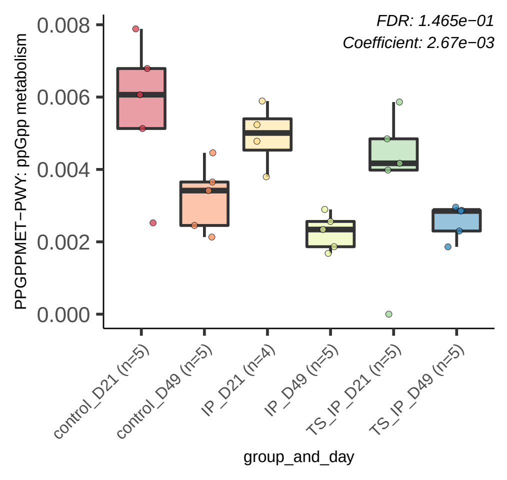

FDR: 1.465e−01
Coefficient: 2.67e−03
PPGPPMET−PWY: ppGpp metabolism
0.008
0.006
0.004
0.002
0.000
control_D21 (n=5)
control_D49 (n=5)
IP_D21 (n=4)
IP_D49 (n=5)
TS_IP_D21 (n=5)
TS_IP_D49 (n=5)
group_and_day

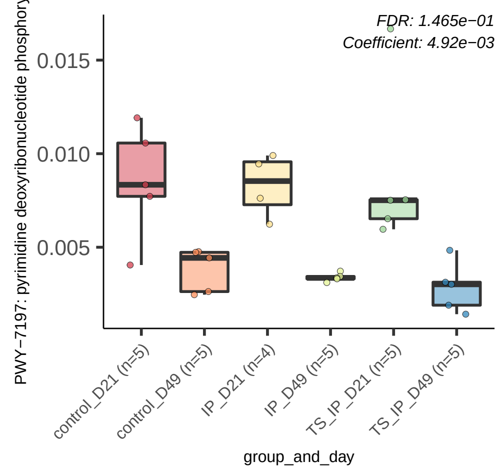

FDR: 1.465e−01
Coefficient: 4.92e−03
PWY−7197: pyrimidine deoxyribonucleotide phosphory
0.015
0.010
0.005
control_D21 (n=5)
control_D49 (n=5)
IP_D21 (n=4)
IP_D49 (n=5)
TS_IP_D21 (n=5)
TS_IP_D49 (n=5)
group_and_day

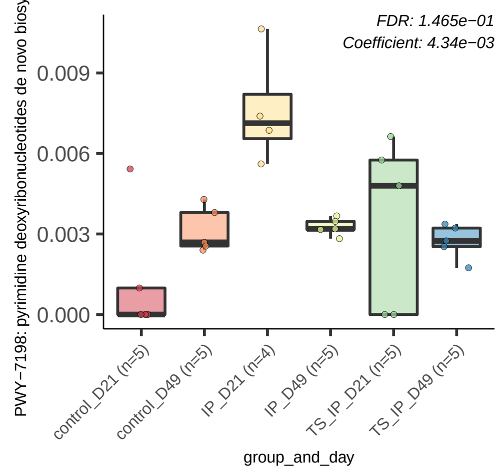

FDR: 1.465e−01
Coefficient: 4.34e−03
PWY−7198: pyrimidine deoxyribonucleotides de novo biosy
0.009
0.006
0.003
0.000
control_D21 (n=5)
control_D49 (n=5)
IP_D21 (n=4)
IP_D49 (n=5)
TS_IP_D21 (n=5)
TS_IP_D49 (n=5)
group_and_day

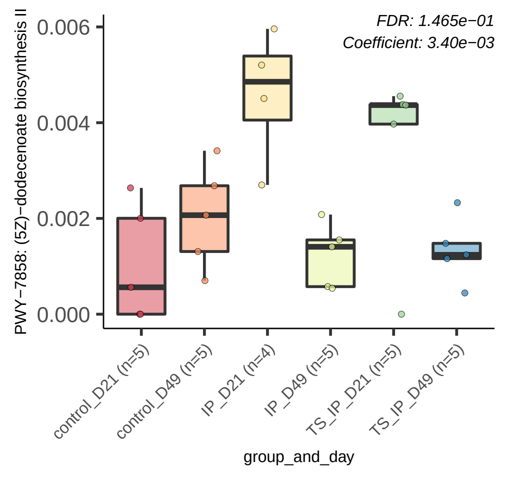

FDR: 1.465e−01
Coefficient: 3.40e−03
PWY−7858: (5Z)−dodecenoate biosynthesis II
0.000
0.002
0.004
0.006
control_D21 (n=5)
control_D49 (n=5)
IP_D21 (n=4)
IP_D49 (n=5)
TS_IP_D21 (n=5)
TS_IP_D49 (n=5)
group_and_day

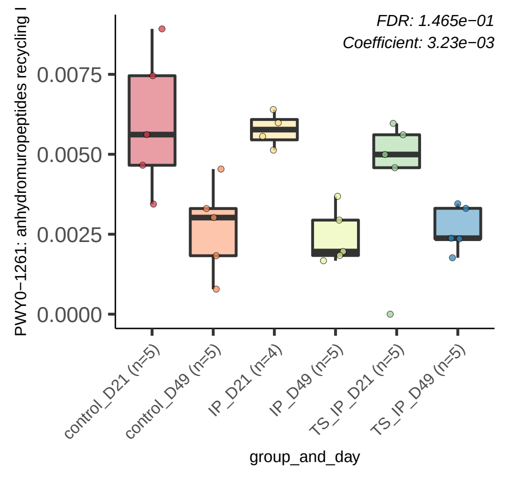
FDR: 1.465e−01
Coefficient: 3.23e−03
PWY0−1261: anhydromuropeptides recycling I
0.0075
0.0050
0.0025
0.0000
control_D21 (n=5)
control_D49 (n=5)
IP_D21 (n=4)
IP_D49 (n=5)
TS_IP_D21 (n=5)
TS_IP_D49 (n=5)
group_and_day

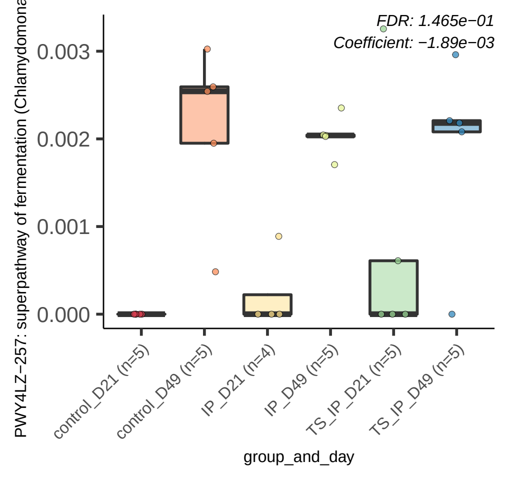
FDR: 1.465e−01
Coefficient: −1.89e−03
PWY4LZ−257: superpathway of fermentation (Chlamydomona
0.003
0.002
0.001
0.000
control_D21 (n=5)
control_D49 (n=5)
IP_D21 (n=4)
IP_D49 (n=5)
TS_IP_D21 (n=5)
TS_IP_D49 (n=5)
group_and_day

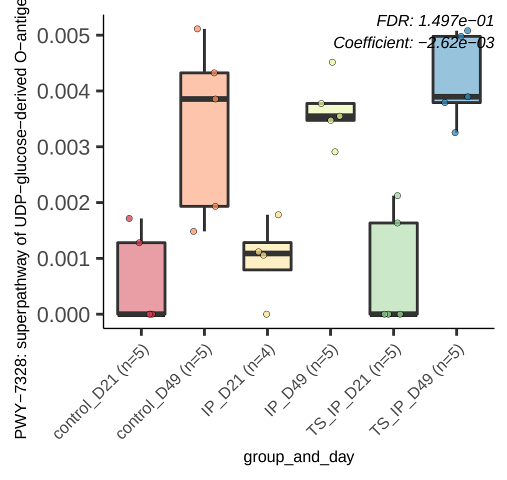
FDR: 1.497e−01
Coefficient: −2.62e−03
PWY−7328: superpathway of UDP−glucose−derived O−antige
0.005
0.004
0.003
0.002
0.001
0.000
control_D21 (n=5)
control_D49 (n=5)
IP_D21 (n=4)
IP_D49 (n=5)
TS_IP_D21 (n=5)
TS_IP_D49 (n=5)
group_and_day

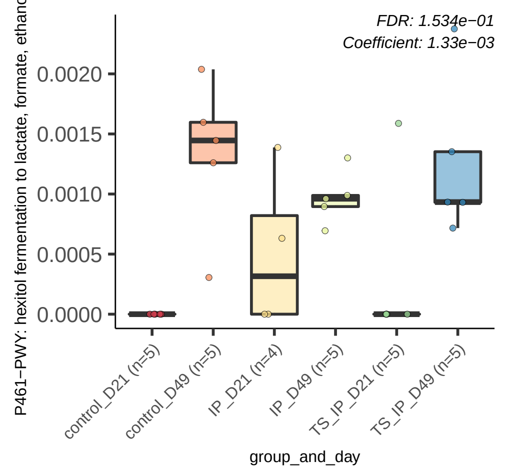
FDR: 1.534e−01
Coefficient: 1.33e−03
P461−PWY: hexitol fermentation to lactate, formate, ethano
0.0020
0.0015
0.0010
0.0005
0.0000
control_D21 (n=5)
control_D49 (n=5)
IP_D21 (n=4)
IP_D49 (n=5)
TS_IP_D21 (n=5)
TS_IP_D49 (n=5)
group_and_day

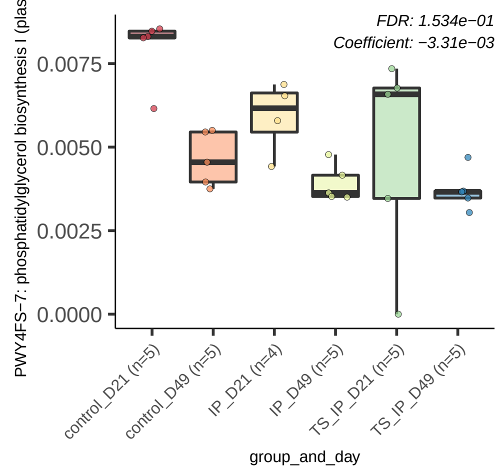

FDR: 1.534e−01
Coefficient: −3.31e−03
PWY4FS−7: phosphatidylglycerol biosynthesis I (plas
0.0075
0.0050
0.0025
0.0000
control_D21 (n=5)
control_D49 (n=5)
IP_D21 (n=4)
IP_D49 (n=5)
TS_IP_D21 (n=5)
TS_IP_D49 (n=5)
group_and_day

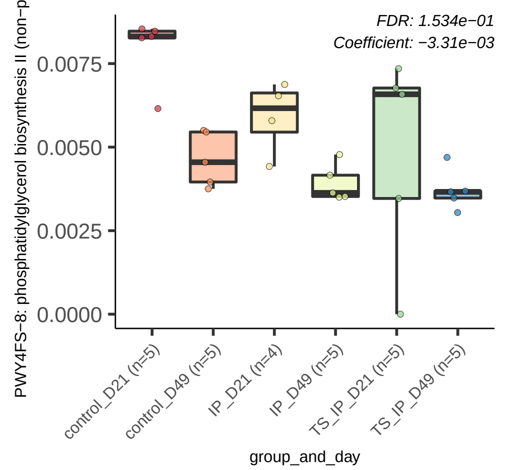

FDR: 1.534e−01
Coefficient: −3.31e−03
PWY4FS−8: phosphatidylglycerol biosynthesis II (non−p
0.0075
0.0050
0.0025
0.0000
control_D21 (n=5)
control_D49 (n=5)
IP_D21 (n=4)
IP_D49 (n=5)
TS_IP_D21 (n=5)
TS_IP_D49 (n=5)
group_and_day

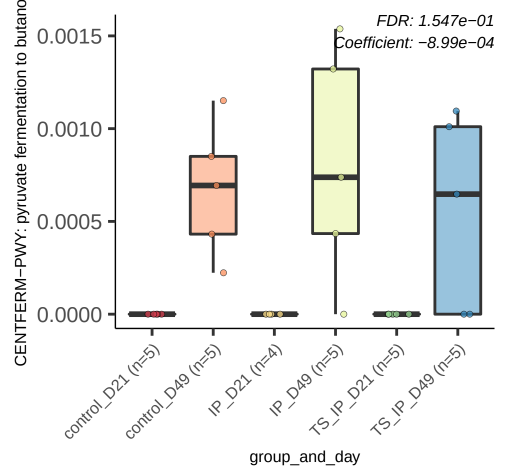

FDR: 1.547e−01
Coefficient: −8.99e−04
CENTFERM−PWY: pyruvate fermentation to butano
0.0015
0.0010
0.0005
0.0000
control_D21 (n=5)
control_D49 (n=5)
IP_D21 (n=4)
IP_D49 (n=5)
TS_IP_D21 (n=5)
TS_IP_D49 (n=5)
group_and_day

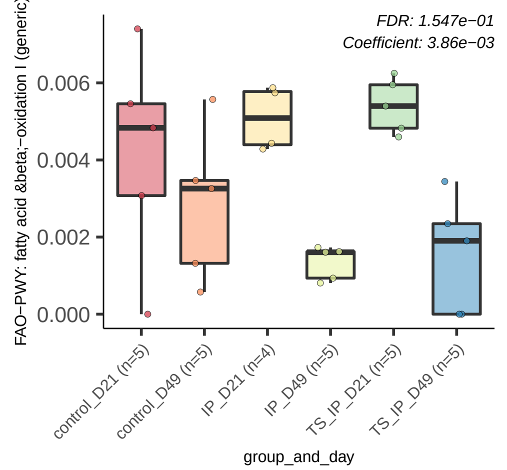
FDR: 1.547e−01
Coefficient: 3.86e−03
FAO−PWY: fatty acid β−oxidation I (generic)
0.006
0.004
0.002
0.000
control_D21 (n=5)
control_D49 (n=5)
IP_D21 (n=4)
IP_D49 (n=5)
TS_IP_D21 (n=5)
TS_IP_D49 (n=5)
group_and_day

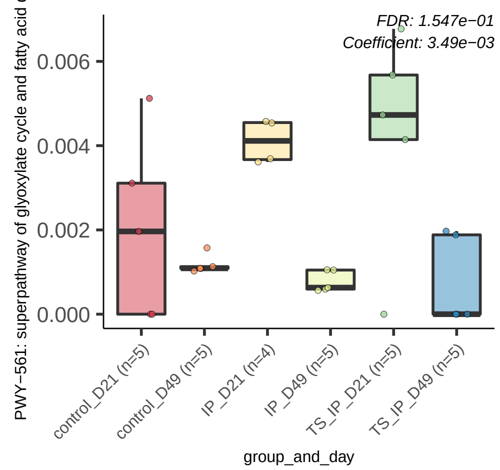
FDR: 1.547e−01
Coefficient: 3.49e−03
PWY−561: superpathway of glyoxylate cycle and fatty acid d
0.006
0.004
0.002
0.000
control_D21 (n=5)
control_D49 (n=5)
IP_D21 (n=4)
IP_D49 (n=5)
TS_IP_D21 (n=5)
TS_IP_D49 (n=5)
group_and_day

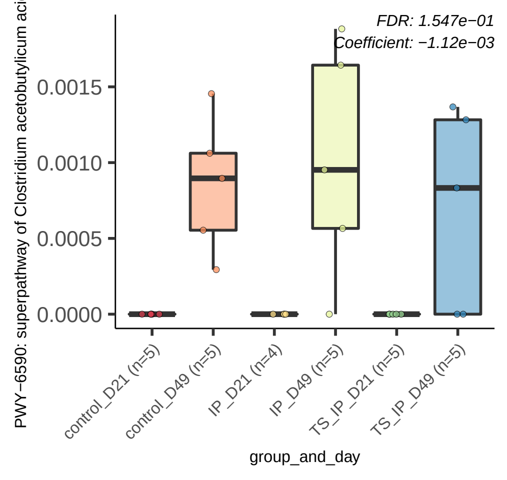
FDR: 1.547e−01
Coefficient: −1.12e−03
PWY−6590: superpathway of Clostridium acetobutylicum aci
0.0015
0.0010
0.0005
0.0000
control_D21 (n=5)
control_D49 (n=5)
IP_D21 (n=4)
IP_D49 (n=5)
TS_IP_D21 (n=5)
TS_IP_D49 (n=5)
group_and_day

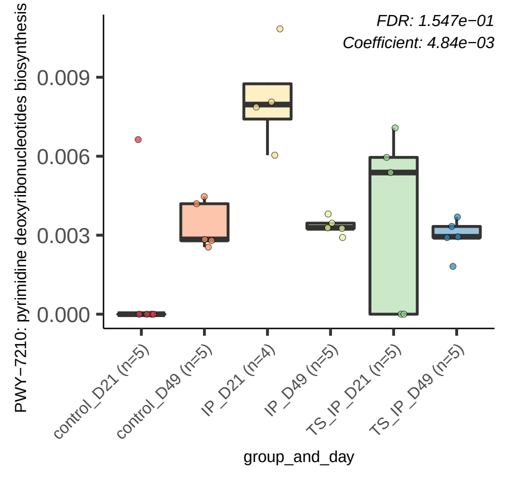
FDR: 1.547e−01
Coefficient: 4.84e−03
PWY−7210: pyrimidine deoxyribonucleotides biosynthesis
0.009
0.006
0.003
0.000
control_D21 (n=5)
control_D49 (n=5)
IP_D21 (n=4)
IP_D49 (n=5)
TS_IP_D21 (n=5)
TS_IP_D49 (n=5)
group_and_day

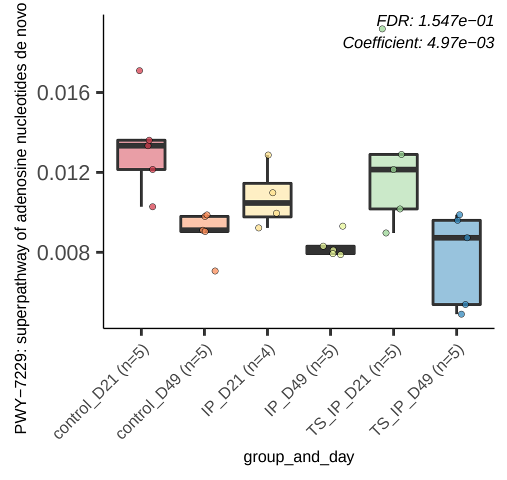
FDR: 1.547e−01
Coefficient: 4.97e−03
PWY−7229: superpathway of adenosine nucleotides de novo
0.016
0.012
0.008
control_D21 (n=5)
control_D49 (n=5)
IP_D21 (n=4)
IP_D49 (n=5)
TS_IP_D21 (n=5)
TS_IP_D49 (n=5)
group_and_day

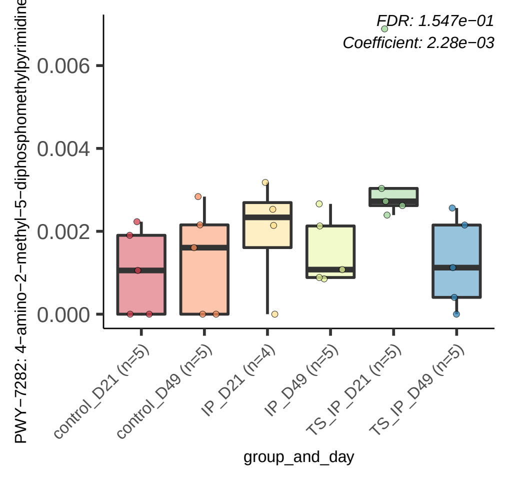

FDR: 1.547e−01
Coefficient: 2.28e−03
PWY−7282: 4−amino−2−methyl−5−diphosphomethylpyrimidine
0.006
0.004
0.002
0.000
control_D21 (n=5)
control_D49 (n=5)
IP_D21 (n=4)
IP_D49 (n=5)
TS_IP_D21 (n=5)
TS_IP_D49 (n=5)
group_and_day

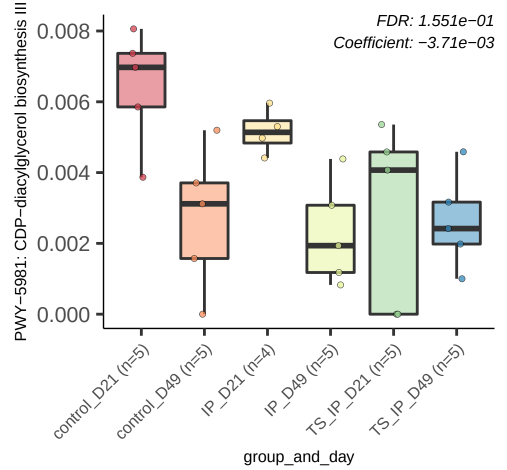

FDR: 1.551e−01
Coefficient: −3.71e−03
PWY−5981: CDP−diacylglycerol biosynthesis III
0.008
0.006
0.004
0.002
0.000
control_D21 (n=5)
control_D49 (n=5)
IP_D21 (n=4)
IP_D49 (n=5)
TS_IP_D21 (n=5)
TS_IP_D49 (n=5)
group_and_day

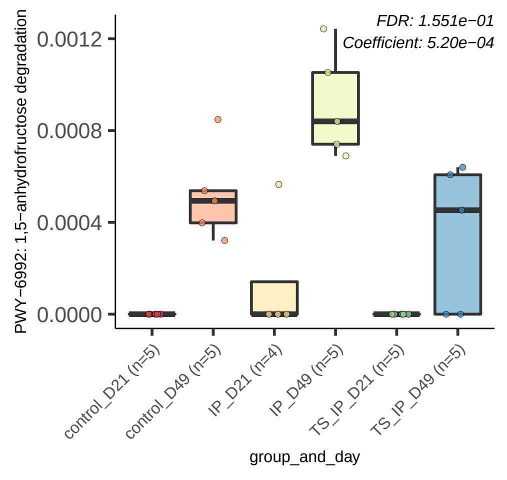

FDR: 1.551e−01
Coefficient: 5.20e−04
PWY−6992: 1,5−anhydrofructose degradation
0.0012
0.0008
0.0004
0.0000
control_D21 (n=5)
control_D49 (n=5)
IP_D21 (n=4)
IP_D49 (n=5)
TS_IP_D21 (n=5)
TS_IP_D49 (n=5)
group_and_day

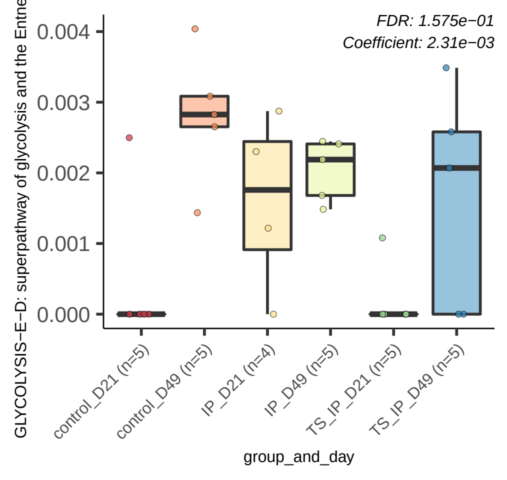
FDR: 1.575e−01
Coefficient: 2.31e−03
GLYCOLYSIS−E−D: superpathway of glycolysis and the Entne
0.004
0.003
0.002
0.001
0.000
control_D21 (n=5)
control_D49 (n=5)
IP_D21 (n=4)
IP_D49 (n=5)
TS_IP_D21 (n=5)
TS_IP_D49 (n=5)
group_and_day

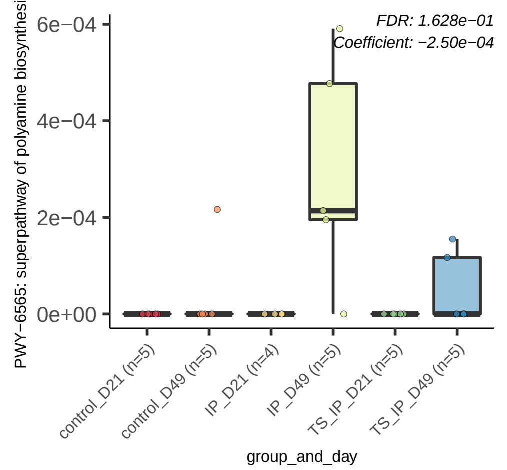
FDR: 1.628e−01
Coefficient: −2.50e−04
PWY−6565: superpathway of polyamine biosynthesi
6e−04
4e−04
2e−04
0e+00
control_D21 (n=5)
control_D49 (n=5)
IP_D21 (n=4)
IP_D49 (n=5)
TS_IP_D21 (n=5)
TS_IP_D49 (n=5)
group_and_day

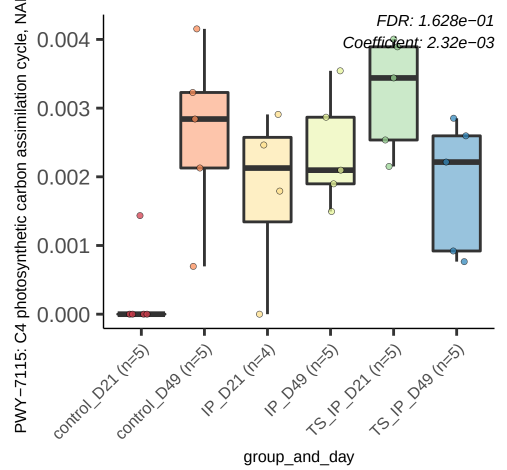

FDR: 1.628e−01
Coefficient: 2.32e−03
PWY−7115: C4 photosynthetic carbon assimilation cycle, NAD
0.004
0.003
0.002
0.001
0.000
control_D21 (n=5)
control_D49 (n=5)
IP_D21 (n=4)
IP_D49 (n=5)
TS_IP_D21 (n=5)
TS_IP_D49 (n=5)
group_and_day

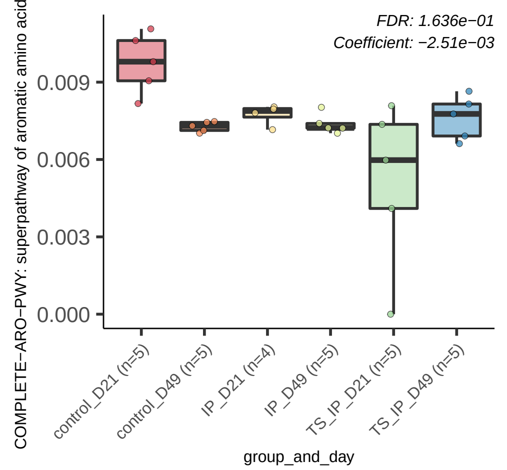

FDR: 1.636e−01
Coefficient: −2.51e−03
COMPLETE−ARO−PWY: superpathway of aromatic amino acid
0.009
0.006
0.003
0.000
control_D21 (n=5)
control_D49 (n=5)
IP_D21 (n=4)
IP_D49 (n=5)
TS_IP_D21 (n=5)
TS_IP_D49 (n=5)
group_and_day

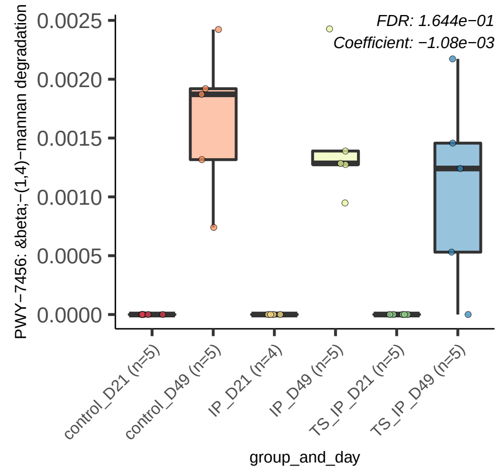

FDR: 1.644e−01
Coefficient: −1.08e−03
PWY−7456: β−(1,4)−mannan degradation
0.0025
0.0020
0.0015
0.0010
0.0005
0.0000
control_D21 (n=5)
control_D49 (n=5)
IP_D21 (n=4)
IP_D49 (n=5)
TS_IP_D21 (n=5)
TS_IP_D49 (n=5)
group_and_day

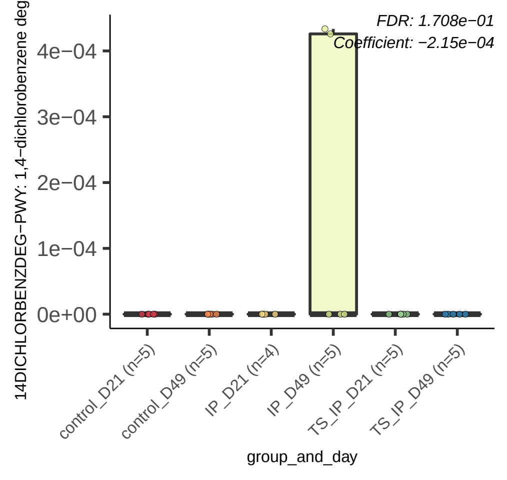
FDR: 1.708e−01
Coefficient: −2.15e−04
14DICHLORBENZDEG−PWY: 1,4−dichlorobenzene deg
4e−04
3e−04
2e−04
1e−04
0e+00
control_D21 (n=5)
control_D49 (n=5)
IP_D21 (n=4)
IP_D49 (n=5)
TS_IP_D21 (n=5)
TS_IP_D49 (n=5)
group_and_day

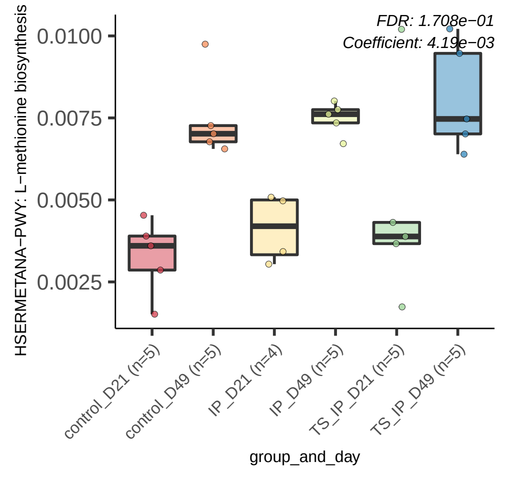

FDR: 1.708e−01
Coefficient: 4.19e−03
HSERMETANA−PWY: L−methionine biosynthesis
0.0100
0.0075
0.0050
0.0025
control_D21 (n=5)
control_D49 (n=5)
IP_D21 (n=4)
IP_D49 (n=5)
TS_IP_D21 (n=5)
TS_IP_D49 (n=5)
group_and_day

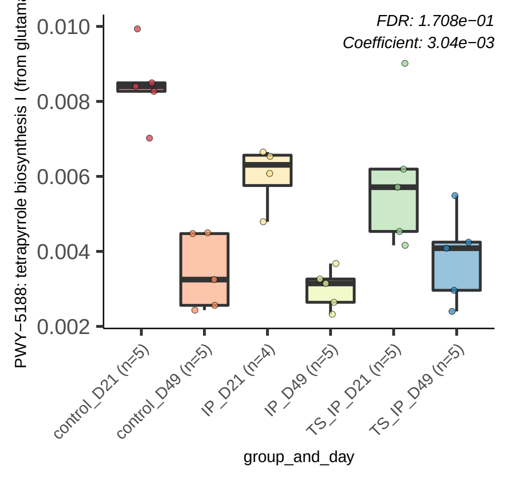
FDR: 1.708e−01
Coefficient: 3.04e−03
PWY−5188: tetrapyrrole biosynthesis I (from glutama
0.010
0.008
0.006
0.004
0.002
control_D21 (n=5)
control_D49 (n=5)
IP_D21 (n=4)
IP_D49 (n=5)
TS_IP_D21 (n=5)
TS_IP_D49 (n=5)
group_and_day

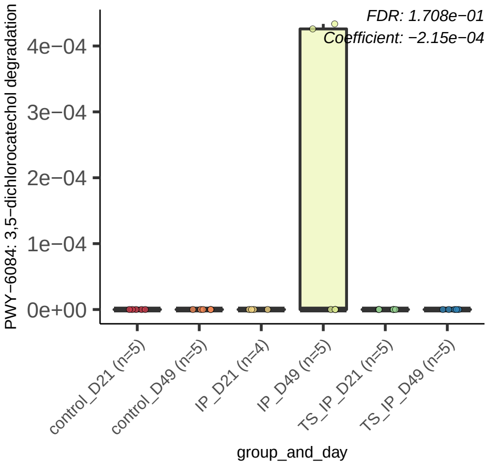
FDR: 1.708e−01
Coefficient: −2.15e−04
PWY−6084: 3,5−dichlorocatechol degradation
4e−04
3e−04
2e−04
1e−04
0e+00
control_D21 (n=5)
control_D49 (n=5)
IP_D21 (n=4)
IP_D49 (n=5)
TS_IP_D21 (n=5)
TS_IP_D49 (n=5)
group_and_day

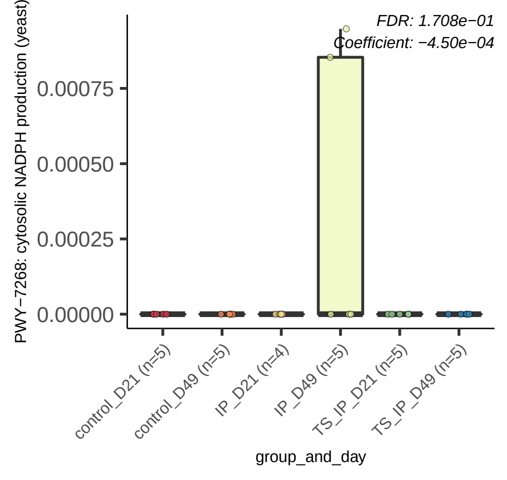

FDR: 1.708e−01
Coefficient: −4.50e−04
PWY−7268: cytosolic NADPH production (yeast)
0.00075
0.00050
0.00025
0.00000
control_D21 (n=5)
control_D49 (n=5)
IP_D21 (n=4)
IP_D49 (n=5)
TS_IP_D21 (n=5)
TS_IP_D49 (n=5)
group_and_day

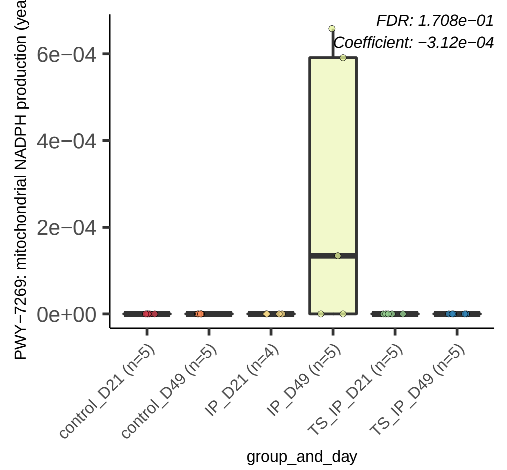

FDR: 1.708e−01
Coefficient: −3.12e−04
PWY−7269: mitochondrial NADPH production (yea
6e−04
4e−04
2e−04
0e+00
control_D21 (n=5)
control_D49 (n=5)
IP_D21 (n=4)
IP_D49 (n=5)
TS_IP_D21 (n=5)
TS_IP_D49 (n=5)
group_and_day

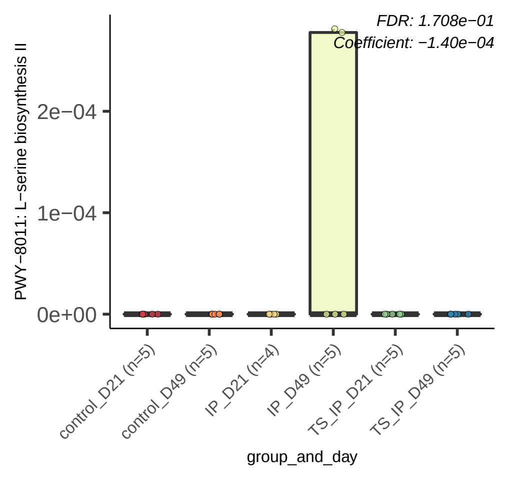

FDR: 1.708e−01
Coefficient: −1.40e−04
PWY−8011: L−serine biosynthesis II
2e−04
1e−04
0e+00
control_D21 (n=5)
control_D49 (n=5)
IP_D21 (n=4)
IP_D49 (n=5)
TS_IP_D21 (n=5)
TS_IP_D49 (n=5)
group_and_day

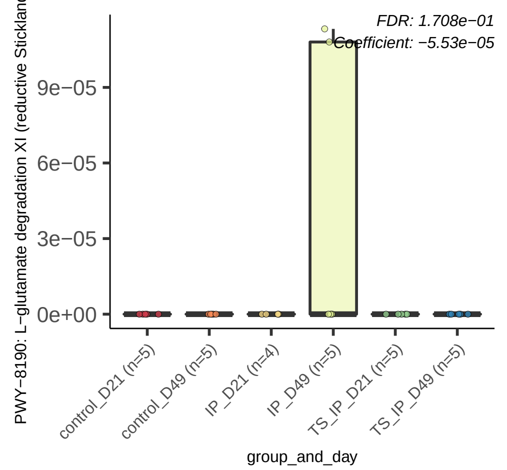
FDR: 1.708e−01
Coefficient: −5.53e−05
PWY−8190: L−glutamate degradation XI (reductive Stickland
9e−05
6e−05
3e−05
0e+00
control_D21 (n=5)
control_D49 (n=5)
IP_D21 (n=4)
IP_D49 (n=5)
TS_IP_D21 (n=5)
TS_IP_D49 (n=5)
group_and_day

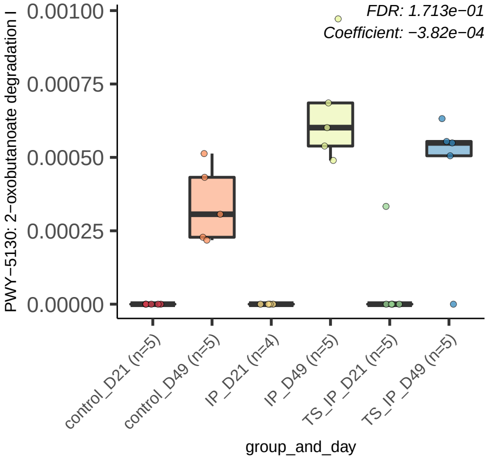

FDR: 1.713e−01
Coefficient: −3.82e−04
PWY−5130: 2−oxobutanoate degradation I
0.00100
0.00075
0.00050
0.00025
0.00000
control_D21 (n=5)
control_D49 (n=5)
IP_D21 (n=4)
IP_D49 (n=5)
TS_IP_D21 (n=5)
TS_IP_D49 (n=5)
group_and_day

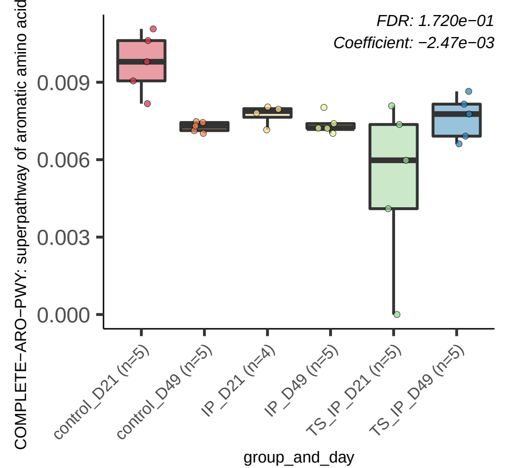
FDR: 1.720e−01
Coefficient: −2.47e−03
COMPLETE−ARO−PWY: superpathway of aromatic amino acid
0.009
0.006
0.003
0.000
control_D21 (n=5)
control_D49 (n=5)
IP_D21 (n=4)
IP_D49 (n=5)
TS_IP_D21 (n=5)
TS_IP_D49 (n=5)
group_and_day

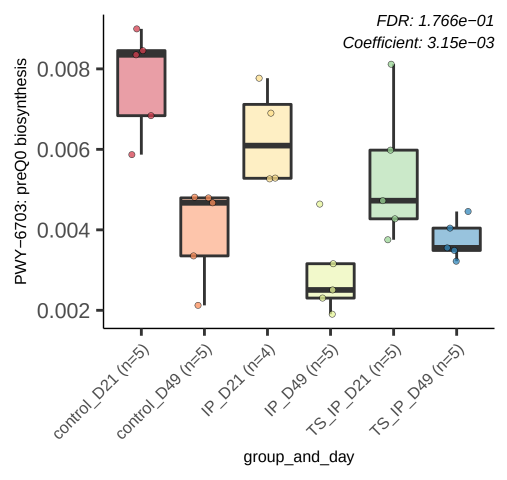
FDR: 1.766e−01
Coefficient: 3.15e−03
PWY−6703: preQ0 biosynthesis
0.008
0.006
0.004
0.002
control_D21 (n=5)
control_D49 (n=5)
IP_D21 (n=4)
IP_D49 (n=5)
TS_IP_D21 (n=5)
TS_IP_D49 (n=5)
group_and_day

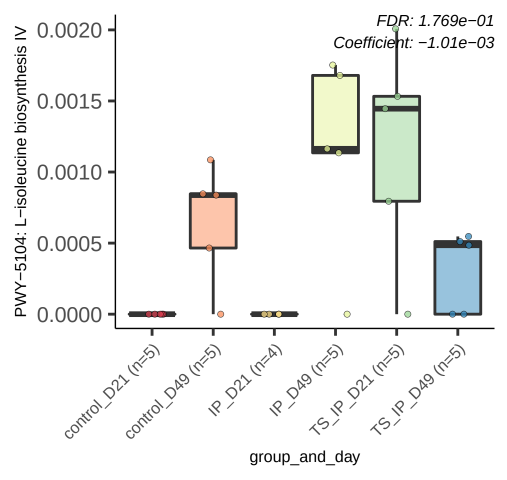
FDR: 1.769e−01
Coefficient: −1.01e−03
PWY−5104: L−isoleucine biosynthesis IV
0.0020
0.0015
0.0010
0.0005
0.0000
control_D21 (n=5)
control_D49 (n=5)
IP_D21 (n=4)
IP_D49 (n=5)
TS_IP_D21 (n=5)
TS_IP_D49 (n=5)
group_and_day

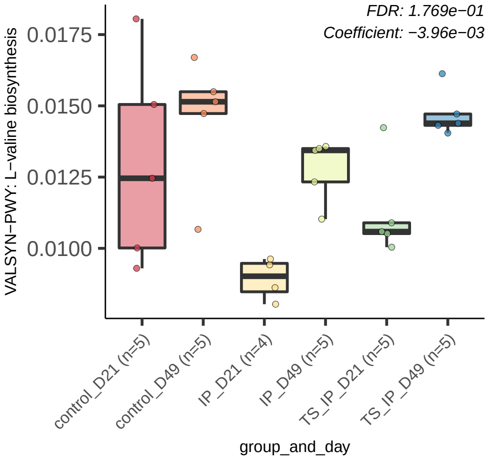

FDR: 1.769e−01
Coefficient: −3.96e−03
VALSYN−PWY: L−valine biosynthesis
0.0175
0.0150
0.0125
0.0100
control_D21 (n=5)
control_D49 (n=5)
IP_D21 (n=4)
IP_D49 (n=5)
TS_IP_D21 (n=5)
TS_IP_D49 (n=5)
group_and_day

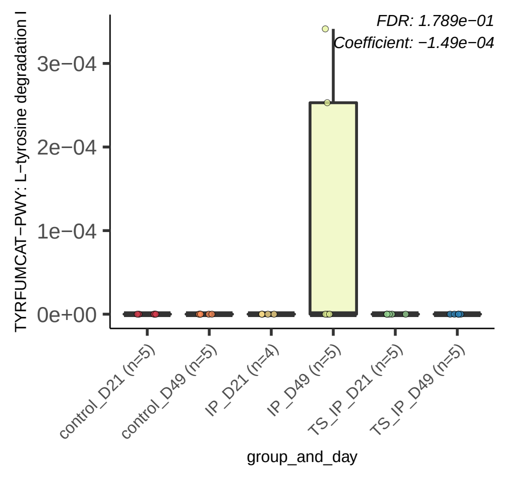

FDR: 1.789e−01
Coefficient: −1.49e−04
TYRFUMCAT−PWY: L−tyrosine degradation I
3e−04
2e−04
1e−04
0e+00
control_D21 (n=5)
control_D49 (n=5)
IP_D21 (n=4)
IP_D49 (n=5)
TS_IP_D21 (n=5)
TS_IP_D49 (n=5)
group_and_day

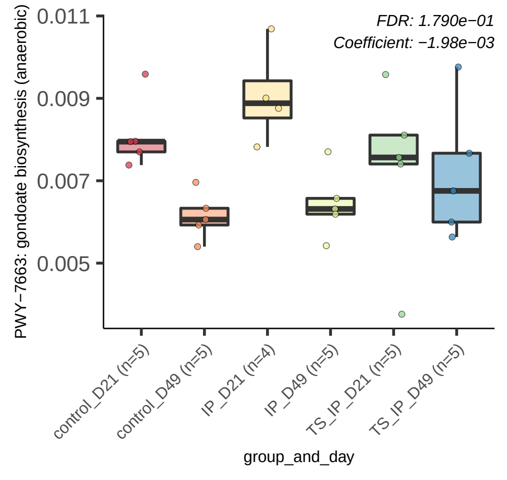
FDR: 1.790e−01
Coefficient: −1.98e−03
PWY−7663: gondoate biosynthesis (anaerobic)
0.011
0.009
0.007
0.005
control_D21 (n=5)
control_D49 (n=5)
IP_D21 (n=4)
IP_D49 (n=5)
TS_IP_D21 (n=5)
TS_IP_D49 (n=5)
group_and_day

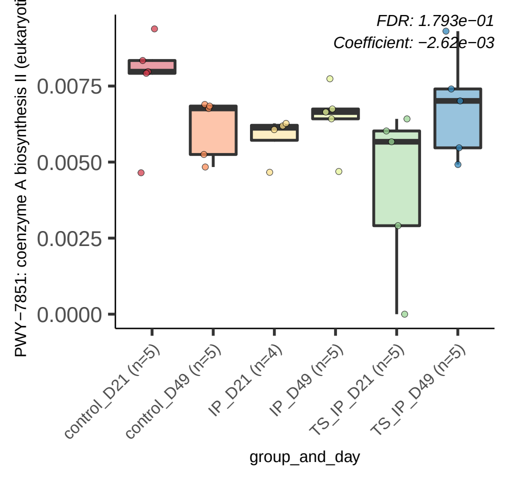

FDR: 1.793e−01
Coefficient: −2.62e−03
PWY−7851: coenzyme A biosynthesis II (eukaryoti
0.0075
0.0050
0.0025
0.0000
control_D21 (n=5)
control_D49 (n=5)
IP_D21 (n=4)
IP_D49 (n=5)
TS_IP_D21 (n=5)
TS_IP_D49 (n=5)
group_and_day

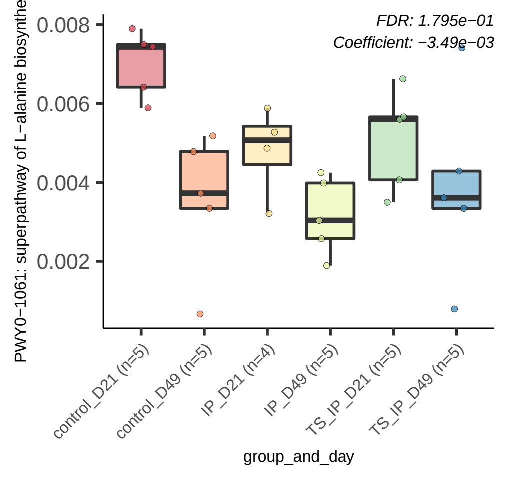
FDR: 1.795e−01
Coefficient: −3.49e−03
PWY0−1061: superpathway of L−alanine biosynthe
0.008
0.006
0.004
0.002
control_D21 (n=5)
control_D49 (n=5)
IP_D21 (n=4)
IP_D49 (n=5)
TS_IP_D21 (n=5)
TS_IP_D49 (n=5)
group_and_day

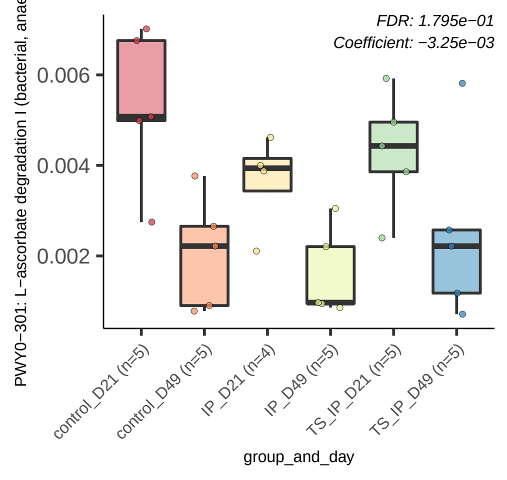
FDR: 1.795e−01
Coefficient: −3.25e−03
PWY0−301: L−ascorbate degradation I (bacterial, anae
0.006
0.004
0.002
control_D21 (n=5)
control_D49 (n=5)
IP_D21 (n=4)
IP_D49 (n=5)
TS_IP_D21 (n=5)
TS_IP_D49 (n=5)
group_and_day

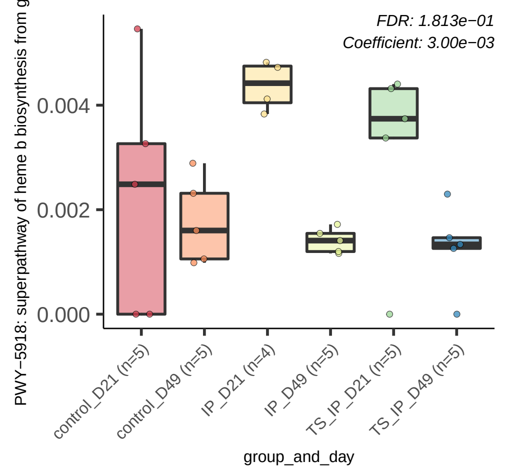

FDR: 1.813e−01
Coefficient: 3.00e−03
PWY−5918: superpathway of heme b biosynthesis from g
0.004
0.002
0.000
control_D21 (n=5)
control_D49 (n=5)
IP_D21 (n=4)
IP_D49 (n=5)
TS_IP_D21 (n=5)
TS_IP_D49 (n=5)
group_and_day

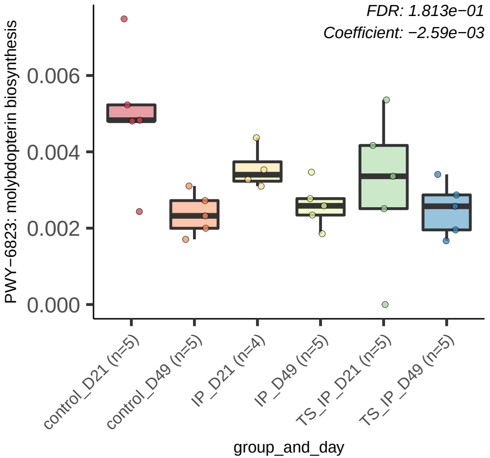
FDR: 1.813e−01
Coefficient: −2.59e−03
PWY−6823: molybdopterin biosynthesis
0.006
0.004
0.002
0.000
control_D21 (n=5)
control_D49 (n=5)
IP_D21 (n=4)
IP_D49 (n=5)
TS_IP_D21 (n=5)
TS_IP_D49 (n=5)
group_and_day

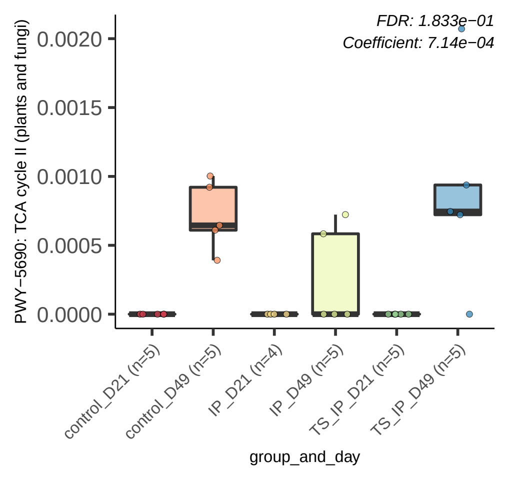
FDR: 1.833e−01
Coefficient: 7.14e−04
PWY−5690: TCA cycle II (plants and fungi)
0.0020
0.0015
0.0010
0.0005
0.0000
control_D21 (n=5)
control_D49 (n=5)
IP_D21 (n=4)
IP_D49 (n=5)
TS_IP_D21 (n=5)
TS_IP_D49 (n=5)
group_and_day

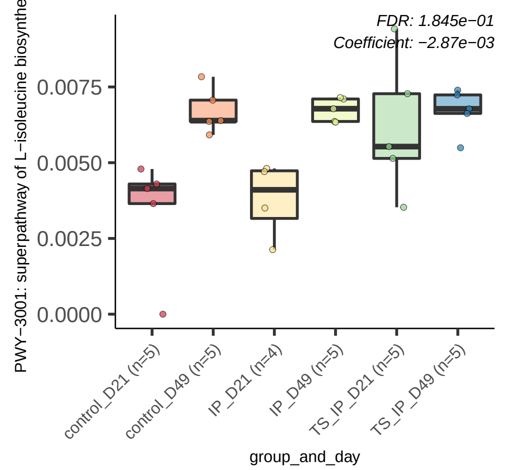
FDR: 1.845e−01
Coefficient: −2.87e−03
PWY−3001: superpathway of L−isoleucine biosynthe
0.0075
0.0050
0.0025
0.0000
control_D21 (n=5)
control_D49 (n=5)
IP_D21 (n=4)
IP_D49 (n=5)
TS_IP_D21 (n=5)
TS_IP_D49 (n=5)
group_and_day

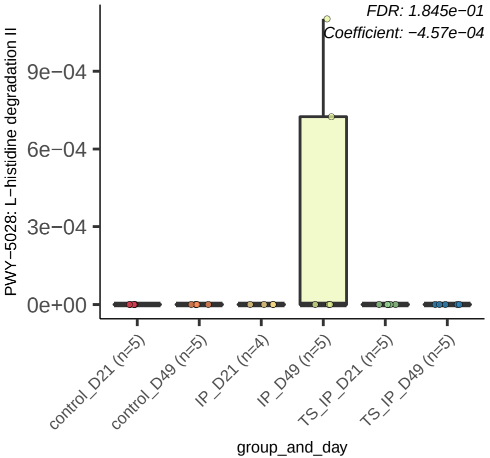
FDR: 1.845e−01
Coefficient: −4.57e−04
PWY−5028: L−histidine degradation II
9e−04
6e−04
3e−04
0e+00
control_D21 (n=5)
control_D49 (n=5)
IP_D21 (n=4)
IP_D49 (n=5)
TS_IP_D21 (n=5)
TS_IP_D49 (n=5)
group_and_day

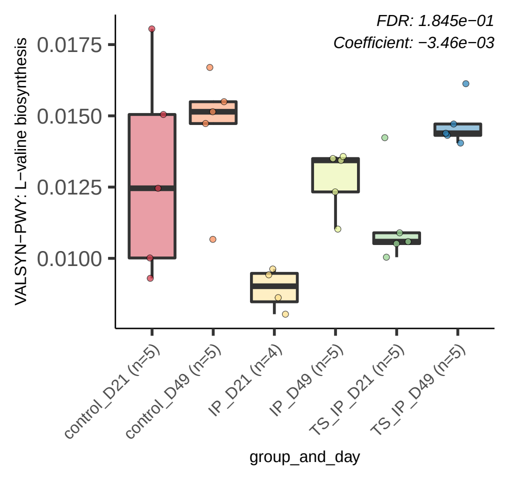
FDR: 1.845e−01
Coefficient: −3.46e−03
VALSYN−PWY: L−valine biosynthesis
0.0175
0.0150
0.0125
0.0100
control_D21 (n=5)
control_D49 (n=5)
IP_D21 (n=4)
IP_D49 (n=5)
TS_IP_D21 (n=5)
TS_IP_D49 (n=5)
group_and_day

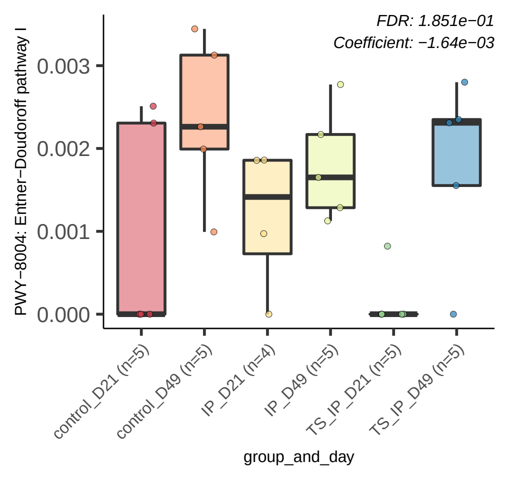

FDR: 1.851e−01
Coefficient: −1.64e−03
PWY−8004: Entner−Doudoroff pathway I
0.000
0.001
0.002
0.003
control_D21 (n=5)
control_D49 (n=5)
IP_D21 (n=4)
IP_D49 (n=5)
TS_IP_D21 (n=5)
TS_IP_D49 (n=5)
group_and_day

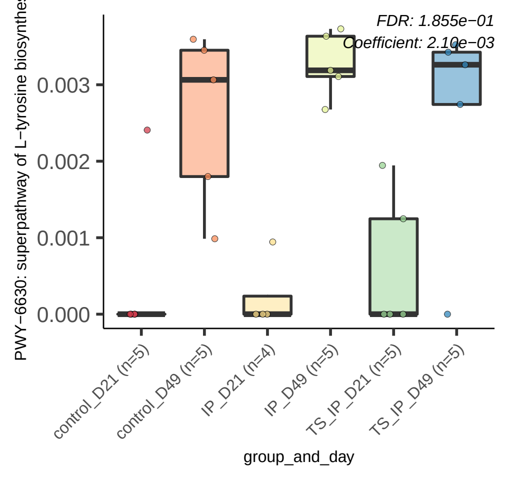

FDR: 1.855e−01
Coefficient: 2.10e−03
PWY−6630: superpathway of L−tyrosine biosynthes
0.000
0.001
0.002
0.003
control_D21 (n=5)
control_D49 (n=5)
IP_D21 (n=4)
IP_D49 (n=5)
TS_IP_D21 (n=5)
TS_IP_D49 (n=5)
group_and_day

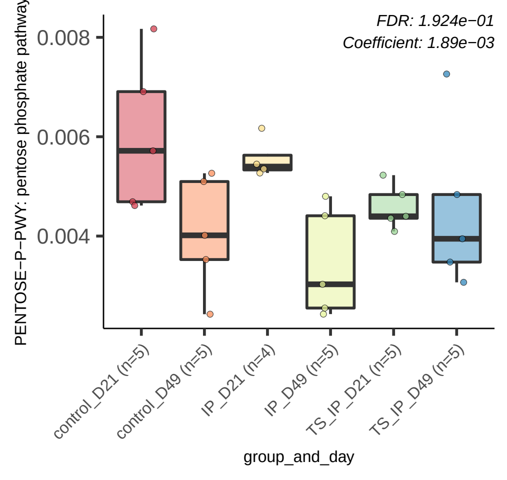

FDR: 1.924e−01
Coefficient: 1.89e−03
PENTOSE−P−PWY: pentose phosphate pathway
0.008
0.006
0.004
control_D21 (n=5)
control_D49 (n=5)
IP_D21 (n=4)
IP_D49 (n=5)
TS_IP_D21 (n=5)
TS_IP_D49 (n=5)
group_and_day

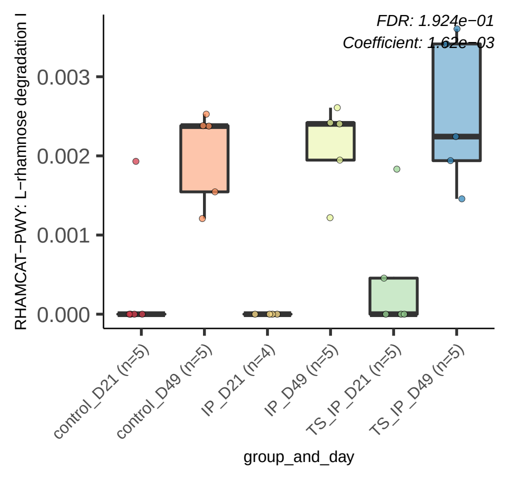
FDR: 1.924e−01
Coefficient: 1.62e−03
RHAMCAT−PWY: L−rhamnose degradation I
0.003
0.002
0.001
0.000
control_D21 (n=5)
control_D49 (n=5)
IP_D21 (n=4)
IP_D49 (n=5)
TS_IP_D21 (n=5)
TS_IP_D49 (n=5)
group_and_day

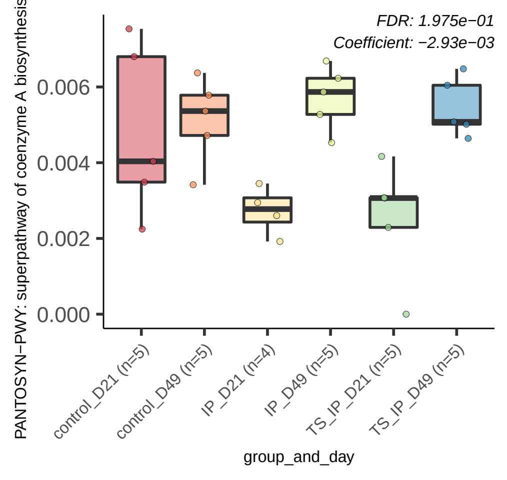

FDR: 1.975e−01
Coefficient: −2.93e−03
PANTOSYN−PWY: superpathway of coenzyme A biosynthesis
0.000
0.002
0.004
0.006
control_D21 (n=5)
control_D49 (n=5)
IP_D21 (n=4)
IP_D49 (n=5)
TS_IP_D21 (n=5)
TS_IP_D49 (n=5)
group_and_day

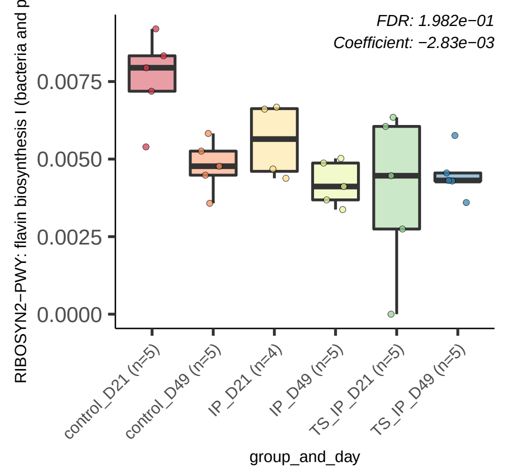
FDR: 1.982e−01
Coefficient: −2.83e−03
RIBOSYN2−PWY: flavin biosynthesis I (bacteria and p
0.0075
0.0050
0.0025
0.0000
control_D21 (n=5)
control_D49 (n=5)
IP_D21 (n=4)
IP_D49 (n=5)
TS_IP_D21 (n=5)
TS_IP_D49 (n=5)
group_and_day

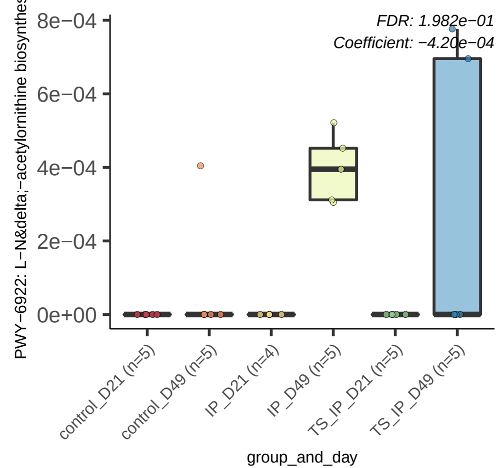

FDR: 1.982e−01
Coefficient: −4.20e−04
PWY−6922: L−Nδ−acetylornithine biosynthes
8e−04
6e−04
4e−04
2e−04
0e+00
control_D21 (n=5)
control_D49 (n=5)
IP_D21 (n=4)
IP_D49 (n=5)
TS_IP_D21 (n=5)
TS_IP_D49 (n=5)
group_and_day

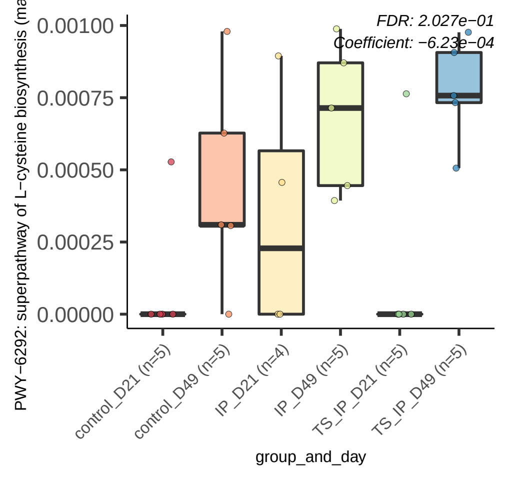

FDR: 2.027e−01
Coefficient: −6.23e−04
PWY−6292: superpathway of L−cysteine biosynthesis (ma
0.00100
0.00075
0.00050
0.00025
0.00000
control_D21 (n=5)
control_D49 (n=5)
IP_D21 (n=4)
IP_D49 (n=5)
TS_IP_D21 (n=5)
TS_IP_D49 (n=5)
group_and_day

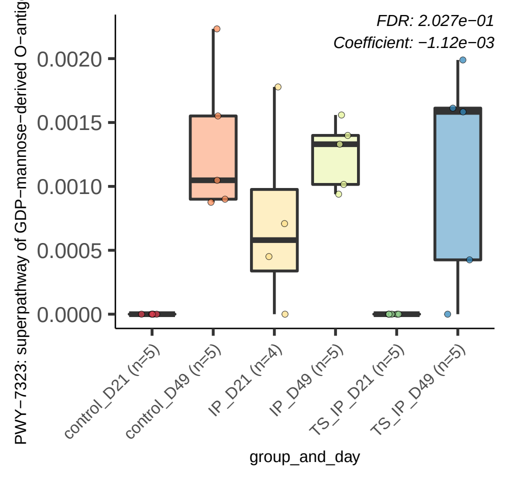
FDR: 2.027e−01
Coefficient: −1.12e−03
PWY−7323: superpathway of GDP−mannose−derived O−antig
0.0020
0.0015
0.0010
0.0005
0.0000
control_D21 (n=5)
control_D49 (n=5)
IP_D21 (n=4)
IP_D49 (n=5)
TS_IP_D21 (n=5)
TS_IP_D49 (n=5)
group_and_day

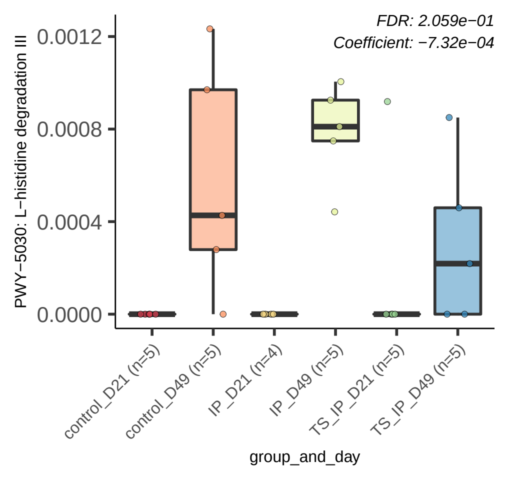

FDR: 2.059e−01
Coefficient: −7.32e−04
PWY−5030: L−histidine degradation III
0.0012
0.0008
0.0004
0.0000
control_D21 (n=5)
control_D49 (n=5)
IP_D21 (n=4)
IP_D49 (n=5)
TS_IP_D21 (n=5)
TS_IP_D49 (n=5)
group_and_day

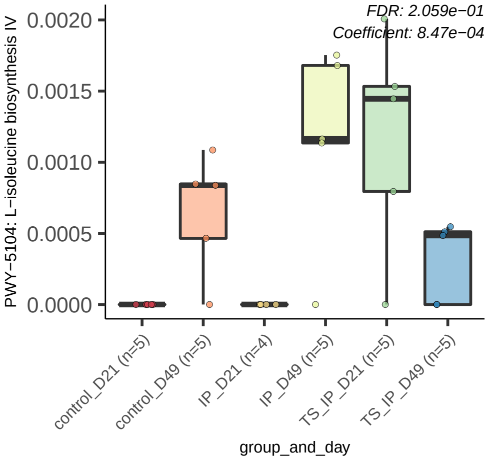
FDR: 2.059e−01
Coefficient: 8.47e−04
PWY−5104: L−isoleucine biosynthesis IV
0.0020
0.0015
0.0010
0.0005
0.0000
control_D21 (n=5)
control_D49 (n=5)
IP_D21 (n=4)
IP_D49 (n=5)
TS_IP_D21 (n=5)
TS_IP_D49 (n=5)
group_and_day

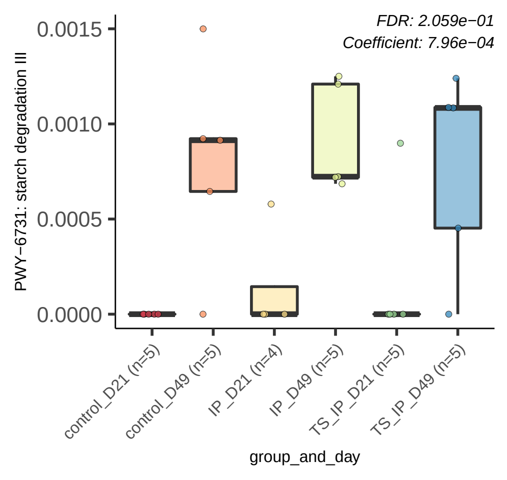

FDR: 2.059e−01
Coefficient: 7.96e−04
PWY−6731: starch degradation III
0.0015
0.0010
0.0005
0.0000
control_D21 (n=5)
control_D49 (n=5)
IP_D21 (n=4)
IP_D49 (n=5)
TS_IP_D21 (n=5)
TS_IP_D49 (n=5)
group_and_day

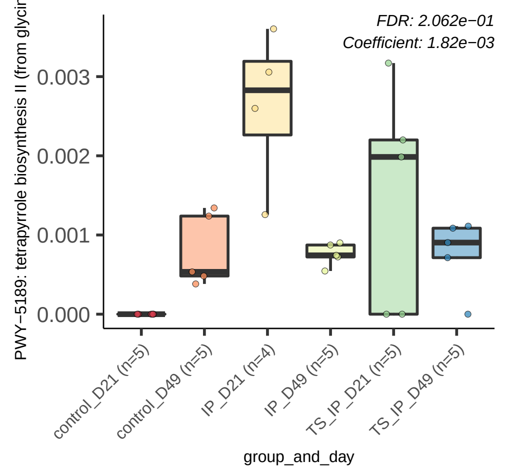
FDR: 2.062e−01
Coefficient: 1.82e−03
PWY−5189: tetrapyrrole biosynthesis II (from glyci
0.003
0.002
0.001
0.000
control_D21 (n=5)
control_D49 (n=5)
IP_D21 (n=4)
IP_D49 (n=5)
TS_IP_D21 (n=5)
TS_IP_D49 (n=5)
group_and_day

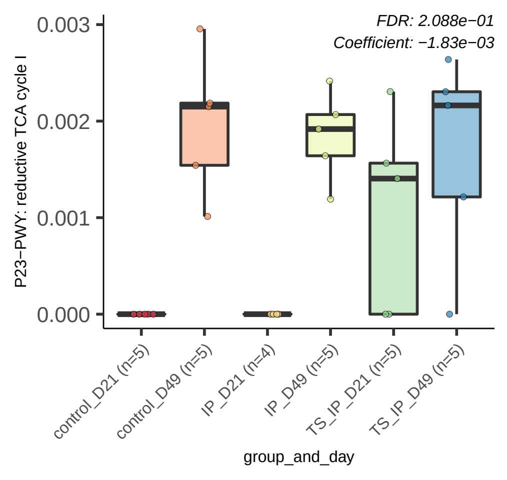

FDR: 2.088e−01
Coefficient: −1.83e−03
P23−PWY: reductive TCA cycle I
0.003
0.002
0.001
0.000
control_D21 (n=5)
control_D49 (n=5)
IP_D21 (n=4)
IP_D49 (n=5)
TS_IP_D21 (n=5)
TS_IP_D49 (n=5)
group_and_day

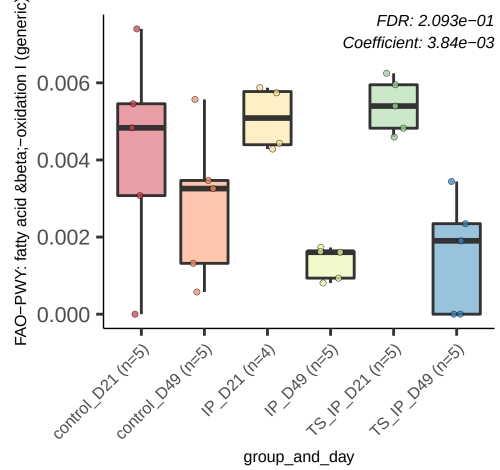
FDR: 2.093e−01
Coefficient: 3.84e−03
FAO−PWY: fatty acid β−oxidation I (generic)
0.006
0.004
0.002
0.000
control_D21 (n=5)
control_D49 (n=5)
IP_D21 (n=4)
IP_D49 (n=5)
TS_IP_D21 (n=5)
TS_IP_D49 (n=5)
group_and_day

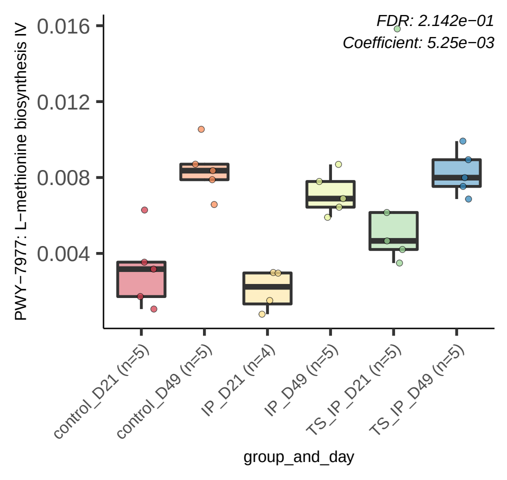

FDR: 2.142e−01
Coefficient: 5.25e−03
PWY−7977: L−methionine biosynthesis IV
0.016
0.012
0.008
0.004
control_D21 (n=5)
control_D49 (n=5)
IP_D21 (n=4)
IP_D49 (n=5)
TS_IP_D21 (n=5)
TS_IP_D49 (n=5)
group_and_day

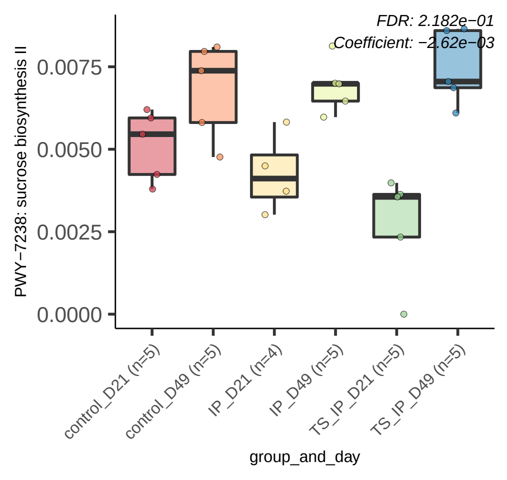

FDR: 2.182e−01
Coefficient: −2.62e−03
PWY−7238: sucrose biosynthesis II
0.0075
0.0050
0.0025
0.0000
control_D21 (n=5)
control_D49 (n=5)
IP_D21 (n=4)
IP_D49 (n=5)
TS_IP_D21 (n=5)
TS_IP_D49 (n=5)
group_and_day

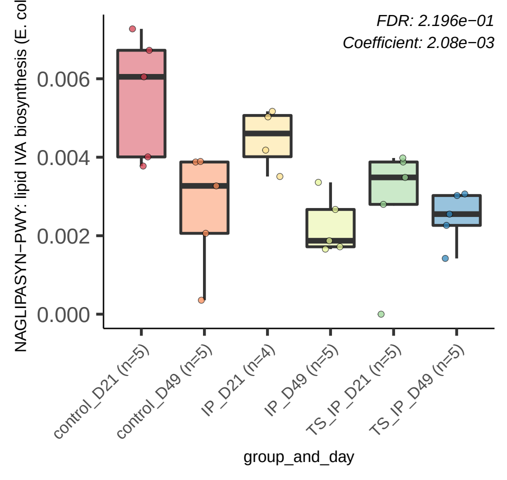

FDR: 2.196e−01
Coefficient: 2.08e−03
NAGLIPASYN−PWY: lipid IVA biosynthesis (E. col
0.006
0.004
0.002
0.000
control_D21 (n=5)
control_D49 (n=5)
IP_D21 (n=4)
IP_D49 (n=5)
TS_IP_D21 (n=5)
TS_IP_D49 (n=5)
group_and_day

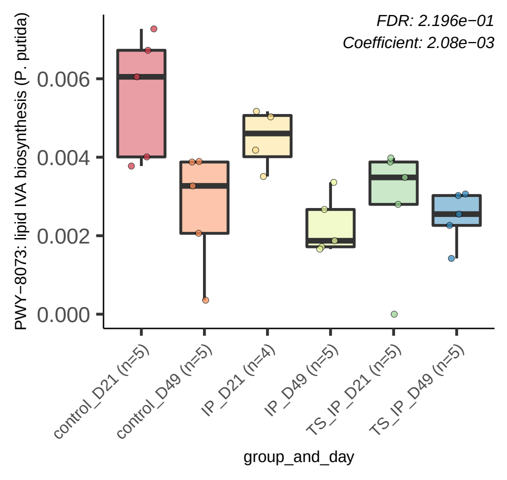
FDR: 2.196e−01
Coefficient: 2.08e−03
PWY−8073: lipid IVA biosynthesis (P. putida)
0.006
0.004
0.002
0.000
control_D21 (n=5)
control_D49 (n=5)
IP_D21 (n=4)
IP_D49 (n=5)
TS_IP_D21 (n=5)
TS_IP_D49 (n=5)
group_and_day

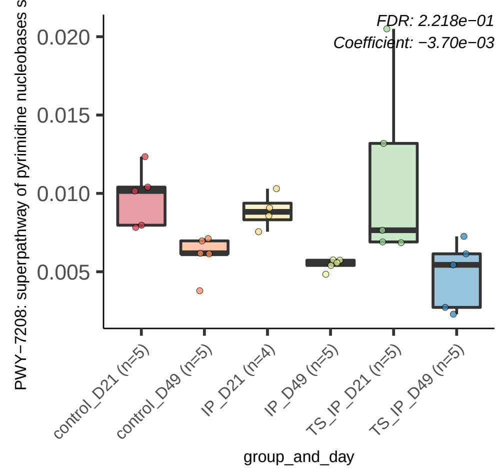

FDR: 2.218e−01
Coefficient: −3.70e−03
PWY−7208: superpathway of pyrimidine nucleobases s
0.020
0.015
0.010
0.005
control_D21 (n=5)
control_D49 (n=5)
IP_D21 (n=4)
IP_D49 (n=5)
TS_IP_D21 (n=5)
TS_IP_D49 (n=5)
group_and_day

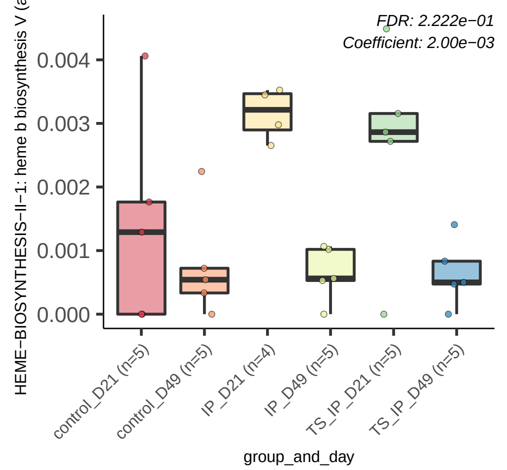
FDR: 2.222e−01
Coefficient: 2.00e−03
HEME−BIOSYNTHESIS−II−1: heme b biosynthesis V (a
0.004
0.003
0.002
0.001
0.000
control_D21 (n=5)
control_D49 (n=5)
IP_D21 (n=4)
IP_D49 (n=5)
TS_IP_D21 (n=5)
TS_IP_D49 (n=5)
group_and_day

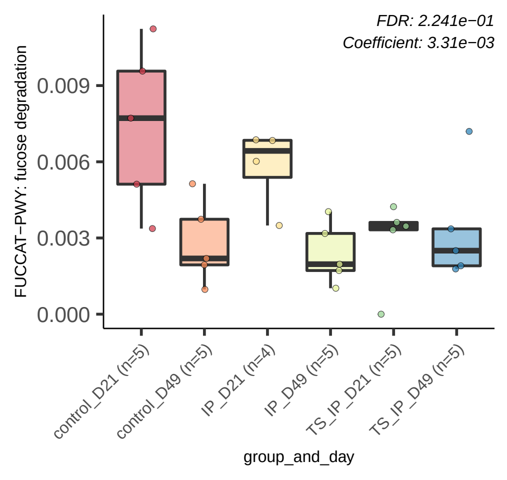
FDR: 2.241e−01
Coefficient: 3.31e−03
FUCCAT−PWY: fucose degradation
0.009
0.006
0.003
0.000
control_D21 (n=5)
control_D49 (n=5)
IP_D21 (n=4)
IP_D49 (n=5)
TS_IP_D21 (n=5)
TS_IP_D49 (n=5)
group_and_day

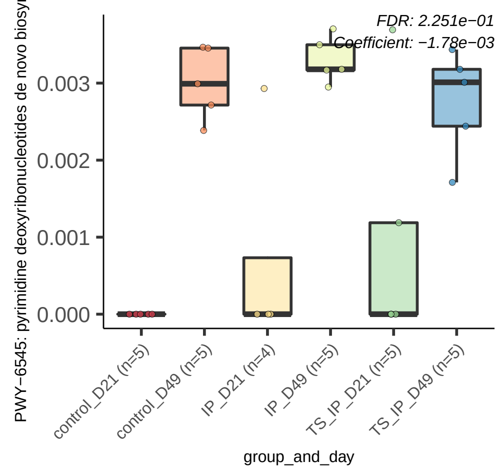
FDR: 2.251e−01
Coefficient: −1.78e−03
PWY−6545: pyrimidine deoxyribonucleotides de novo biosyn
0.000
0.001
0.002
0.003
control_D21 (n=5)
control_D49 (n=5)
IP_D21 (n=4)
IP_D49 (n=5)
TS_IP_D21 (n=5)
TS_IP_D49 (n=5)
group_and_day

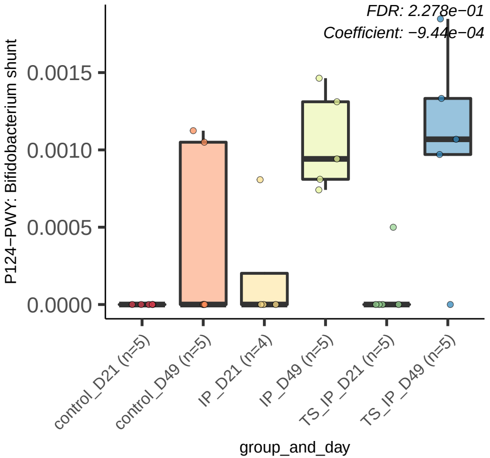
FDR: 2.278e−01
Coefficient: −9.44e−04
P124−PWY: Bifidobacterium shunt
0.0015
0.0010
0.0005
0.0000
control_D21 (n=5)
control_D49 (n=5)
IP_D21 (n=4)
IP_D49 (n=5)
TS_IP_D21 (n=5)
TS_IP_D49 (n=5)
group_and_day

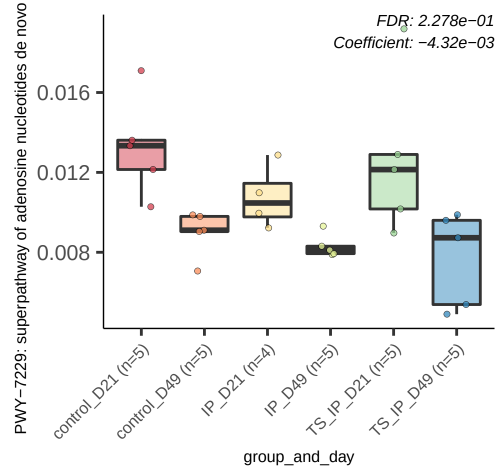
FDR: 2.278e−01
Coefficient: −4.32e−03
PWY−7229: superpathway of adenosine nucleotides de novo
0.016
0.012
0.008
control_D21 (n=5)
control_D49 (n=5)
IP_D21 (n=4)
IP_D49 (n=5)
TS_IP_D21 (n=5)
TS_IP_D49 (n=5)
group_and_day

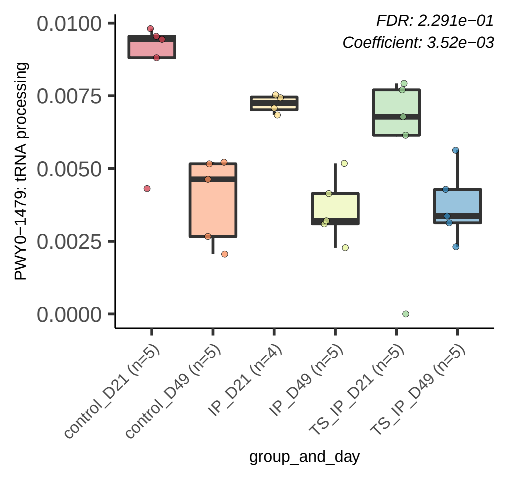

FDR: 2.291e−01
Coefficient: 3.52e−03
PWY0−1479: tRNA processing
0.0100
0.0075
0.0050
0.0025
0.0000
control_D21 (n=5)
control_D49 (n=5)
IP_D21 (n=4)
IP_D49 (n=5)
TS_IP_D21 (n=5)
TS_IP_D49 (n=5)
group_and_day

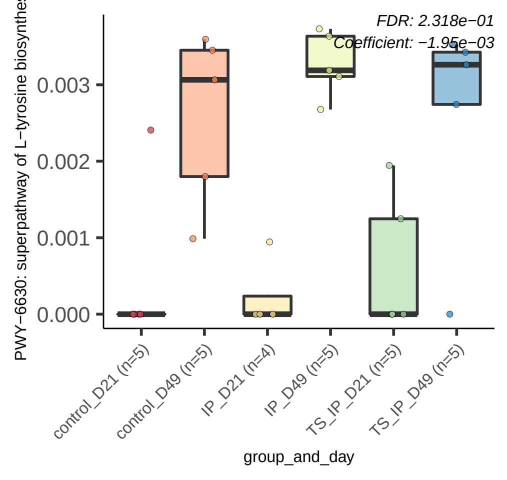
FDR: 2.318e−01
Coefficient: −1.95e−03
PWY−6630: superpathway of L−tyrosine biosynthes
0.003
0.002
0.001
0.000
control_D21 (n=5)
control_D49 (n=5)
IP_D21 (n=4)
IP_D49 (n=5)
TS_IP_D21 (n=5)
TS_IP_D49 (n=5)
group_and_day

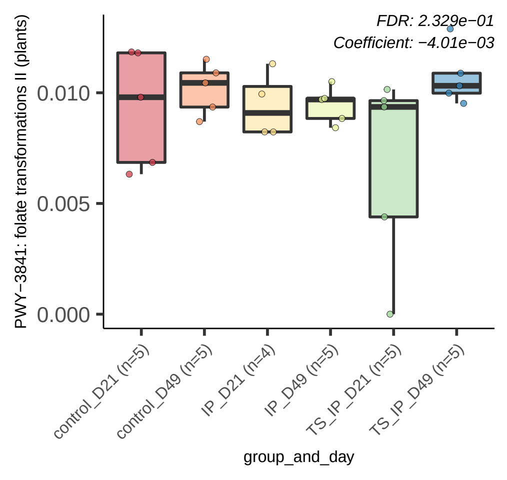

FDR: 2.329e−01
Coefficient: −4.01e−03
PWY−3841: folate transformations II (plants)
0.000
0.005
0.010
control_D21 (n=5)
control_D49 (n=5)
IP_D21 (n=4)
IP_D49 (n=5)
TS_IP_D21 (n=5)
TS_IP_D49 (n=5)
group_and_day

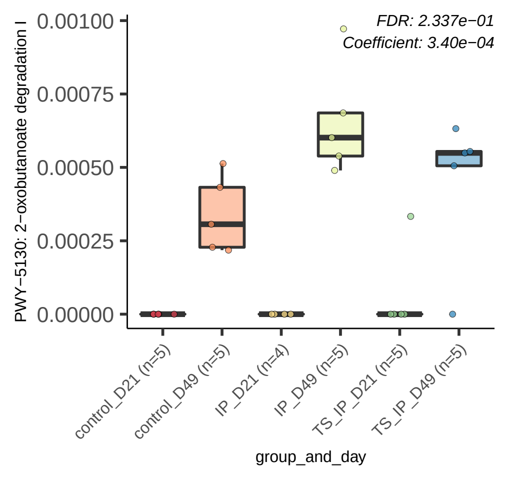
FDR: 2.337e−01
Coefficient: 3.40e−04
PWY−5130: 2−oxobutanoate degradation I
0.00100
0.00075
0.00050
0.00025
0.00000
control_D21 (n=5)
control_D49 (n=5)
IP_D21 (n=4)
IP_D49 (n=5)
TS_IP_D21 (n=5)
TS_IP_D49 (n=5)
group_and_day

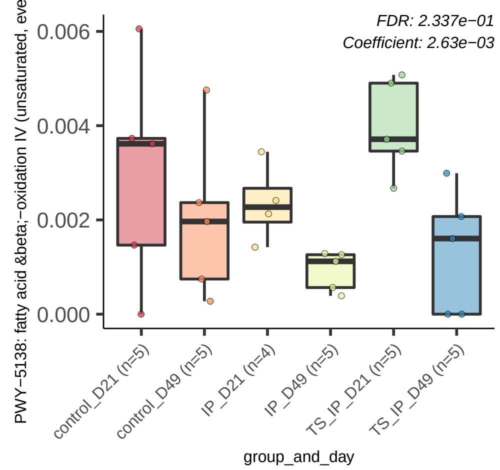
FDR: 2.337e−01
Coefficient: 2.63e−03
PWY−5138: fatty acid β−oxidation IV (unsaturated, eve
0.006
0.004
0.002
0.000
control_D21 (n=5)
control_D49 (n=5)
IP_D21 (n=4)
IP_D49 (n=5)
TS_IP_D21 (n=5)
TS_IP_D49 (n=5)
group_and_day

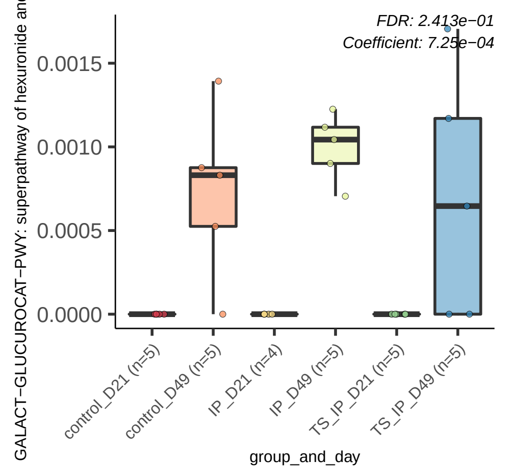
FDR: 2.413e−01
Coefficient: 7.25e−04
GALACT−GLUCUROCAT−PWY: superpathway of hexuronide and
0.0000
0.0005
0.0010
0.0015
control_D21 (n=5)
control_D49 (n=5)
IP_D21 (n=4)
IP_D49 (n=5)
TS_IP_D21 (n=5)
TS_IP_D49 (n=5)
group_and_day

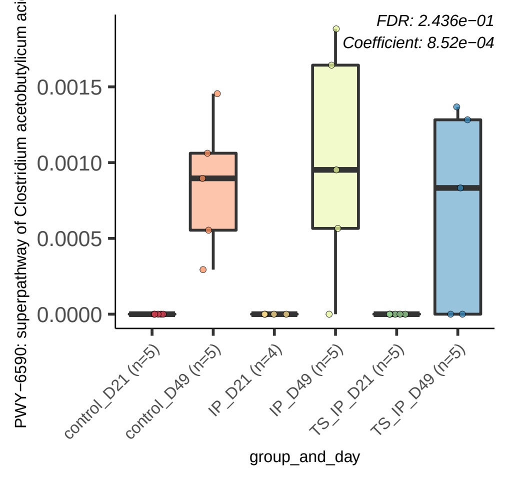
FDR: 2.436e−01
Coefficient: 8.52e−04
PWY−6590: superpathway of Clostridium acetobutylicum aci
0.0015
0.0010
0.0005
0.0000
control_D21 (n=5)
control_D49 (n=5)
IP_D21 (n=4)
IP_D49 (n=5)
TS_IP_D21 (n=5)
TS_IP_D49 (n=5)
group_and_day
